# The MAPSTROKE analysis of the access to stroke reperfusion treatment and stroke units in Italy

**DOI:** 10.1093/esj/aakaf030

**Published:** 2026-02-09

**Authors:** Ettore Nicolini, Antonio Ciacciarelli, Enrica Franchini, André da Silva Frainer, Leonardo da Luz Dorneles, Mateus Boiani, Marcio Dorn, Paola Santalucia, Valeria Caso, Danilo Toni, Leonardo Augusto Carbonera

**Affiliations:** Department of Human Neuroscience, Sapienza University of Rome, Rome, Italy; Stroke Unit, Policlinico Umberto I, Sapienza University of Rome, Rome, Italy; Neurologia-Stroke Unit, Ospedale Provinciale di Bolzano (SABES-ASDAA), Bolzano-Bozen, Italy; Institute of Informatics and Centre for Biotechnology, Federal University of Rio Grande do Sul, Porto Alegre, Brazil; Institute of Informatics and Centre for Biotechnology, Federal University of Rio Grande do Sul, Porto Alegre, Brazil; Institute of Informatics and Centre for Biotechnology, Federal University of Rio Grande do Sul, Porto Alegre, Brazil; Institute of Informatics and Centre for Biotechnology, Federal University of Rio Grande do Sul, Porto Alegre, Brazil; National Centre for Clinical Governance and Excellence of Care, Italian National Institute of Health, Rome, Italy; Stroke Unit, Department of Neurology, Saronno Hospital, ASST Valle Olona, Saronno, Italy; Department of Human Neuroscience, Sapienza University of Rome, Rome, Italy; Department of Neurology and Neurosurgery, Hospital Moinhos de Vento, Porto Alegre, Rio Grande do Sul, Brazil; Pós-Graduação em Medicina: Ciências Médicas, Universidade Federal do Rio Grande do Sul, Porto Alegre, Rio Grande do Sul, Brazil

**Keywords:** algorithms, geographic information systems, health services accessibility, healthcare planning, medical informatics, stroke

## Abstract

**Background:**

Access to reperfusion therapies and stroke unit (SU) admission remains heterogeneous across Europe. Mapping tools can reveal service gaps and guide implementation strategies. MAPSTROKE provides regional mapping of existing stroke centres and identifies potential new sites in underserved areas.

**Aims:**

To apply a computational strategy to the Italian stroke care system to estimate national coverage for reperfusion therapies and quantify SU bed capacity under current constraints.

**Methods:**

Using MAPSTROKE geospatial modelling, we assessed (1) 45-min access to a hospital providing reperfusion treatment and (2) SU bed coverage limited by capacity. Population and stroke incidence data for 2023 were mapped on a hexagonal grid combining sources from the Italian Ministry of Health and the Kontur Dataset. Hospitals were classified as Comprehensive (CSC), Primary (PSC), Acute Stroke-Ready (ASRH) or Potential Acute Stroke Centres (PASC). Isochrones of 45 min were generated for hospitals performing reperfusion. Regional coverage was estimated, and a Partial Set Covering identified the minimal number of PASCs required to achieve ≥ 90% coverage. Stroke unit capacity was estimated using bed counts and mean length of stay (LOS).

**Results:**

Among 535 hospitals (80 CSCs, 132 PSCs, 22 ASRHs, 301 PASCs), 91.7% of strokes were within 45 min of a hospital providing reperfusion treatment. Seven regions were below 90%, 6 achieved this target after optimisation. National SU capacity covered 79.2% of annual incidence, with a gap of 255 beds (158 with ideal LOS).

**Conclusions:**

The MAPSTROKE project reveals adequate reperfusion access but critical SU capacity disparities, underscoring the need for coordinated national strategies.

## Graphical Abstract

**Graphical Abstract:**
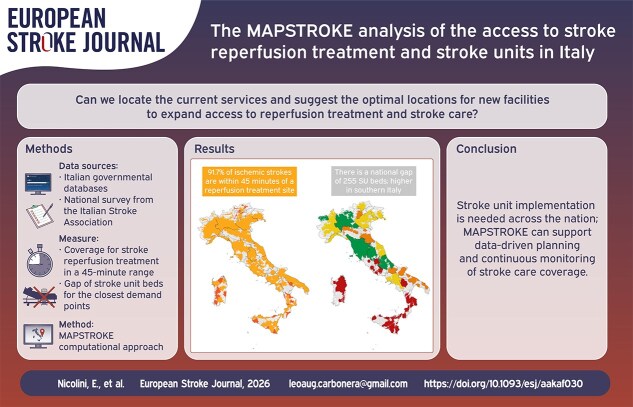

## Introduction

Over the past 3 decades, intravenous thrombolysis (IVT) and EVT have reduced stroke-related disability and mortality.^1,2^ Yet stroke remains the leading cause of adult disability and the second cause of death, and its burden is expected to rise by 2050 with population ageing.^3,4^

Specialised stroke unit (SU) care decreases poor outcomes in ischemic and hemorrhagic stroke.^5^ However, access to treatment is highly variable. In Europe, about 17% of ischemic stroke patients receive IVT and 6.9% undergo EVT, with EVT available to fewer than 1% of patients in 11 countries; reperfusion rates correlate with gross domestic product (GDP).^6^

Europe has 4 SU per million inhabitants, but access ranges from fewer than 1 to 11 per million, and admission is influenced by hospital bed occupancy.^6,7^

The Stroke Action Plan for Europe (SAP-E) recommends that 90% of stroke patients be treated in a dedicated SU.^8^ Technological tools may help reduce regional disparities and optimise resource allocation.

The MAPSTROKE project applies a strategy to map stroke centres, catchment areas, underserved regions and potential centres. A pilot in 9 Latin American countries/regions supported its usefulness for organising stroke networks.^9^ The present study is the first application of MAPSTROKE in Europe.

## Aims

This study applies a CS to the Italian stroke network (1) to estimate the coverage for access to hospitals providing acute reperfusion treatments, and (2) to determine the capacity-constrained coverage of dedicated SU beds at the regional level.

## Patients and methods

We conducted regional geospatial modelling of Italy using the MAPSTROKE project tools to assess coverage of access to hospitals providing acute reperfusion treatments and access to SU beds.

### Data sources

The population grid containing demographic density data was obtained from the Kontur Population Dataset.^10^ We utilised the dataset’s finest available resolution (H3 hexagons at Resolution 8), corresponding to a hexagonal grid with an average cell area of approximately 0.74 km^2^ and edge lengths of approximately 400 m.^11^

Annual stroke incidence by region was extracted from Italian Ministry of Health sources for 2023,^12^ and used to estimate the yearly absolute number of strokes per hexagon.

A census of Italian hospitals was compiled using annual reports from the National Agency for Regional Health Services of the Italian Ministry of Health^12^ and a dedicated survey conducted by the Italian Stroke Association (ISA), presented at the 2024 annual Italian Stroke Conference (unpublished data). From the survey, we extracted information regarding the services available at each hospital.

Hospitals were classified in 4 categories:

- Comprehensive Stroke Centre (CSC): performs both IVT and EVT and admits patients for in-hospital acute and post-acute SU care.
- Primary Stroke Centre (PSC): performs IVT and admits the patient for in-hospital acute and post-acute SU care.
- Acute Stroke-Ready Hospital (ASRH): performs IVT and transfers the patient to other facilities for additional EVT treatment and/or hospitalisation.
- Potential Acute Stroke Centre (PASC): hospitals with an emergency department (ED), neuroimaging and laboratory services, but that do not perform either IVT or EVT and lack established acute stroke care protocols. This definition is based on a previously published MAPSTROKE analysis.^9^

For PSCs and CSCs, information on SU bed counts and the mean length of stay (LOS) was collected.

Hospital geolocations were obtained from OpenStreetMap,^13^ and road networks were derived using OpenRouteService.^14^

#### Analysis 1: 45-min access to hospitals providing reperfusion treatments

For each hospital providing reperfusion treatments (ASRH, PSC and CSC), we generated 45-min road-network isochrones using the hospital location as the seed, based on the previously described methodology.^9^ Isochrones are polygonal boundaries representing areas reachable within the established time frame. This time range was based on previously published benchmarks.^15,16^

The 45-min hospital access coverage was defined as the proportion of ischemic strokes occurring within a 45-min travel-time isochrone to a centre capable of delivering reperfusion treatment. For each hexagonal grid cell, we calculated travel time to the nearest centre using the national road network. Within each Italian region, the sum of incident acute ischemic strokes (AIS) inside the isochrones was divided by the total number of AIS to determine the proportion of coverage.

We modelled the placement of new stroke centres as a Partial Set Covering (PSCLP).^17^ Partial Set Covering determines the minimum number of facilities needed to cover a predefined share of demand; here, we minimised PASC conversions to reach 90% incidence coverage. The optimisation model was solved with the Gurobi Optimizer^18^ in regions with coverage < 90% to identify the minimum number of PASCs requiring conversion. Gurobi is a mathematical programming solver that uses exact algorithms to search the solution space and determine optimal solutions. We also performed a sensitivity analysis using travel-time thresholds of 30 and 60 min. Supplementary Methods 1 provide further details on the mathematical model.

#### Analysis 2: Capacity-constrained SU coverage

Capacity constraint is a definition from the theory of economics of a circumstance in which a service unit (ie, a facility) is incapable of attending a specific demand due to upper limits of resources.^19^ We calculated the capacity of each stroke centre to assist the demand of stroke patients within a geographical territory.

Initially, we estimated the annual admission capacity for hospitals with an SU (PSC and CSC) based on the number of beds and the mean LOS:


$$ Ci= Bi\times \frac{365}{LOSi} $$

where *i* is a given PSC or CSC, *C* is the capacity, *B* is the number of SU beds and *LOS* is the mean length of stay in days. For example, in a hospital with LOS = 3.65 days, 100 strokes can be admitted per bed per year; while longer LOS proportionally will reduce the capacity to admit patients in the SU.

We modelled catchment areas as a Fixed-Centre Districting Problem,^18^ with each PSC/CSC as the centre of a district comprising its assigned demand units. The model promoted geographically compact regions and equilibrium in hospitals’ occupancy rates.

**Figure 1:**
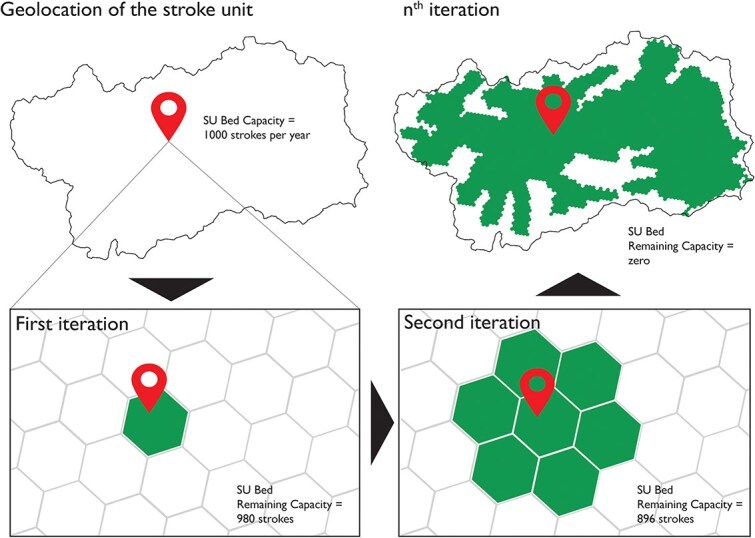
Capacity-constrained catchment area estimation for stroke unit beds in Italy. For each stroke unit (SU), a polygon was iteratively expanded on a hexagonal grid. After each iteration, the number of strokes within the newly added hexagons was subtracted from the hospital’s remaining capacity. The expansion process continued until the hospital’s capacity was fully reached. The resulting polygon defined the capacity-limited catchment area of each hospital—numbers used solely for illustrative purposes.

Because enforcing strict geographic contiguity is computationally demanding, we used a 2-step strategy. First, Gurobi solved an optimisation model prioritising compactness and balanced occupancy without contiguity constraints. Second, because this solution could yield disconnected regions, we applied a heuristic refinement method.^20^ We removed assignments lacking a contiguous path to their hospital and, for each PSC/CSC, iteratively expanded a polygon by adding adjacent hexagons, provided no other hospital had taken them and capacity could accommodate the additional strokes. Stroke counts in added hexagons were subtracted from the remaining capacity until exhaustion (Figure 1). Supplementary Methods 2 detail the mathematical model and solving strategy.

Finally, we produced the regional SU beds coverage. We then produced regional SU bed coverage, defined as the proportion of incident strokes that SU beds could accommodate. For each region, we estimated required capacity, compared it with available beds, calculated the SU bed gap and repeated the analysis assuming an ideal LOS of 3.65 days, derived from the European Stroke Organisation benchmark of 100 strokes per SU bed per year (365/100 = 3.65).^21^

### Validation

The algorithms were developed at the Structural Bioinformatics and Computational Biology Laboratory (SBCB Lab), Universidade Federal do Rio Grande do Sul, Brazil. Hospital lists, service classifications (ASRH/PSC/CSC), bed numbers, LOS information and the geospatial outputs were validated by Italian neurologists affiliated with the Italian Stroke Society (E.N., A.C., E.F.). Software environment and tools used for the geospatial analyses are detailed in Supplementary Methods 3.

### Ethical considerations

MAPSTROKE global was approved by the Research Ethics Committee of Hospital Moinhos de Vento (CAAE 70912123.2.0000.5330). Informed consent was waived.

## Results

### Stroke incidence and hospital characteristics

In 2023, a total of 108,753 stroke cases occurred in Italy, corresponding to an incidence rate of 184.6 cases per 100,000 people per year. Liguria reported the highest incidence (278.3/100,000), nearly double that of Campania, which had the lowest incidence rate (140.8/100,000).

The survey identified 535 hospitals across Italy, including 80 CSCs, 132 PSCs, 22 ASRHs and 301 PASCs. The regional distribution of the centres is shown in Table 1.

**Table 1:** Regional and national demographics and types of hospitals in Italy.

**Region**	**Population**	**Stroke incidence rate (cases per 100,000 population)**	**No. of PASCs**	**No. of ASRHs**	**No. of PSCs**	**No. of CSCs**
Abruzzo	1,268,168	212.6	4	0	4	3
Alto-Adige	513,474	190.7	6	0	0	1
Basilicata	575,278	178.5	1	0	1	0
Calabria	1,833,170	163.5	12	0	2	3
Campania	5,511,802	140.8	31	3	5	7
Emilia-Romagna	4,439,634	219.3	28	0	7	5
Friuli Venezia Giulia	1,185,222	233.5	6	0	2	2
Lazio	5,572,696	185.3	22	9	8	6
Liguria	1,495,709	278.3	6	0	6	2
Lombardia	9,715,944	170.1	48	2	20	16
Marche	1,517,374	193.4	7	0	6	1
Molise	306,018	153.6	2	0	1	0
Piemonte	4,287,392	198.2	13	2	18	5
Puglia	4,087,687	145.8	18	0	6	6
Sardegna	1,619,709	177.3	17	0	0	4
Sicilia	4,906,676	167.4	34	1	8	6
Toscana	3,619,160	223.6	13	1	19	3
Trentino	543,957	190.8	5	1	0	1
Umbria	892,656	230.5	7	0	5	2
Valle d’Aosta	131,643	195.2	0	0	0	1
Veneto	4,890,419	191.8	21	3	14	6
**Total**	58,913,788	184.6	301	22	132	80

Abbreviations: ASRH = Acute Stroke Ready Hospital; CSC = Comprehensive Stroke Centre; PASC = Potential Acute Stroke Centre; PSC = Primary Stroke Centre.

All PSCs reported having a dedicated SU. The only CSC without a dedicated SU transfers all patients immediately after the EVT. Full-time ED services, plain CT scan and laboratory services were nearly universal across hospital categories, except for 7 PASCs (limited ED hours and laboratory services, open 12 h daily) and 16 PASCs lacking 24-h daily service for plain CT scan (limited CT h, open 12 h daily).

Further characteristics are detailed in Table S1.

#### Analysis 1: The 45-min access to acute reperfusion treatments

Considering the ground distance to any ASRH, PSC or CSC and respecting regional borders, 91.7% of incident AIS in Italy can reach a hospital providing reperfusion treatment within 45 min. This coverage varies by region, ranging from 33.9% in Basilicata to 98.4% in Liguria. The national coverage of CSC alone was 75.7%, with an uneven interregional distribution, spanning from 93.2% in Lombardia to no coverage in Basilicata and Molise (Table 2). The summary of the national and regional 45-minute coverage of the distinct types of hospitals is detailed in Table S2. The list of hospitals that contributed to the regional coverage for access to reperfusion treatment is available in Table S3. When applying the 30- and 60-min thresholds, the national coverage was altered to 79.6% and 96%, respectively. Regional variation across the different time thresholds is available in Table S4.

**Table 2:** Absolute and relative access to reperfusion treatments for ischemic stroke in Italy, grouped by hospital types.

**Region**	**No. of AIS cases per year**	**Stroke cases within 45 min of a CSC (%)**	**Stroke cases within 45 min of an ASRH, PSC or CSC (%)**	**Stroke cases within 45 min of a PASC, ASRH, PSC or CSC—after optimisation (%)**
Abruzzo	1,553	1,014 (65.3)	1,464 (94.3)	NA
Alto-Adige	688	432 (62.8)	432 (62.8)	652 (94.7)
Basilicata	564	0 (0.0)	191 (33.9)	292 (51.8)
Calabria	1,772	893 (50.4)	1,190 (67.2)	1,600 (90.3)
Campania	4,700	3,899 (83.0)	4,458 (94.9)	NA
Emilia-Romagna	6,120	5,032 (82.2)	5,671 (92.7)	NA
Friuli-Venezia Giulia	1,672	1,165 (69.7)	1,586 (94.9)	NA
Lazio	5,883	4,821 (81.9)	5,680 (96.6)	NA
Liguria	2,746	1,992 (72.6)	2,702 (98.4)	NA
Lombardia	10,798	10,063 (93.2)	10,567 (97.9)	NA
Marche	1,686	593 (35.2)	1,554 (92.2)	NA
Molise	342	0 (0.0)	136 (39.7)	316 (91.4)
Piemonte	5,459	4,373 (80.1)	5,330 (97.7)	NA
Puglia	3,755	3,191 (85.0)	3,504 (93.3)	NA
Sardegna	1,811	1,151 (63.5)	1,151 (63.5)	1,637 (90.4)
Sicilia	5,359	3,147 (58.7)	4,399 (82.1)	4,832 (90.2)
Toscana	4,834	3,235 (66.9)	4,535 (93.8)	NA
Trentino	588	405 (68.9)	436 (74.2)	552 (93.8)
Umbria	1,312	942 (71.8)	1,289 (98.2)	NA
Valle d’Aosta	144	130 (90.2)	130 (90.2)	NA
Veneto	6,098	5,073 (83.2)	5,861 (96.1)	NA
**Italy**	**67,884**	**51,551 (75.7)**	**62,266 (91.7)**	**64,212 (94.6)**

The last column shows the coverage achieved after the optimisation strategy, which involves converting PASC to ASRH in the regions where the coverage of ASRH, PSC and CSC is below 90%.

Abbreviations: AIS = acute ischemic stroke; ASRH = Acute Stroke Ready Hospital; CSC = Comprehensive Stroke Centre; NA = not applicable (optimisation not performed as current coverage is 90% or higher); PASC = Potential Acute Stroke Centre; PSC = Primary Stroke Centre.

The optimisation strategy was applied to the 7 regions with coverage below 90%. After the conversion of 23 PASC (1 in Basilicata, 2 in Molise, 3 in Alto Adige, Trentino and Sicilia, 5 in Sardegna and 6 in Calabria), all regions except Basilicata had sufficient PASCs that could be converted to ASRHs to reach the 90% target. Figure 2 shows the map with 45-min coverage before and after the optimisation. Maps at the regional level are provided in Figures S1–S21.

**Figure 2:**
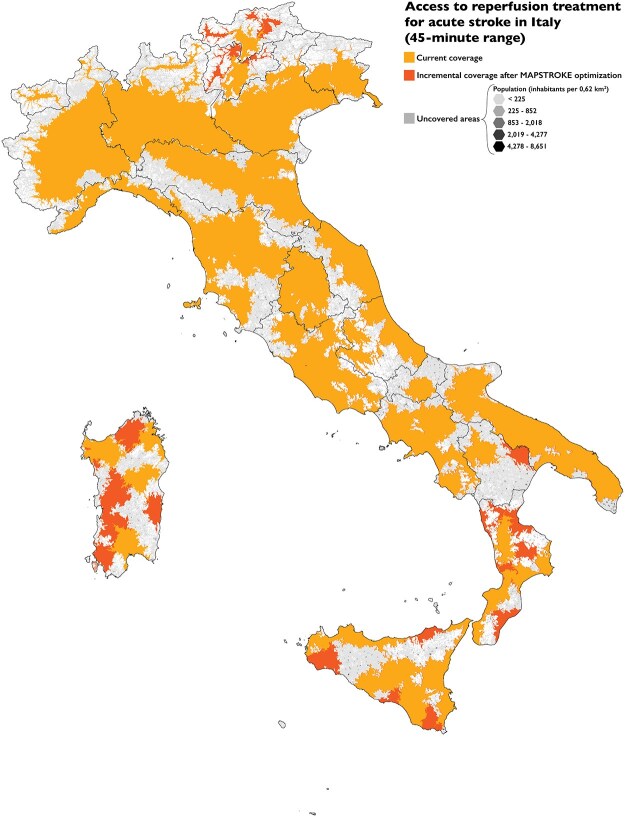
Acute stroke reperfusion treatment coverage in Italy based on a 45-min travel time. Isochrones were merged respecting regional borders. In the background, the hexagonal grid is painted according to the population density, and is visible in uncovered areas. Darker hexagons represent regions with a higher number of stroke cases.

#### Analysis 2: Capacity-constrained SU coverage

Primary Stroke Centres reported a median of 4 beds (IQR, 4–6; range, 1–14), while CSCs had 8 (IQR, 6–8; range, 2–14). The median length of stay (LOS) in the SU was 5 days for both PSCs (IQR 4–7) and CSCs (IQR 4–6). Based on these data, the national absolute SU capacity was calculated to be 86,090 patients/year, corresponding to 79.2% of the annual national stroke incidence. As shown in Table 3, only 5 regions had sufficient SU capacity to admit at least 90% of their regional incidence: Abruzzo (92.3%), Lombardia (94.1%), Toscana (97.3%), Umbria (91.2%) and Valle d’Aosta (99.4%). Maximum coverage time was consistently below 180 min. The coverage of stroke unit beds, by hospital and region, is detailed in Table S5.

**Table 3:** Description of the capacity-constrained SU coverage per Italian region.

**Region**	**No. of stroke cases per year**	**SU capacity (%)**	**Distance to SU bed (median in minutes)** **[IQR; max]**	**Gap of SU beds for 90% coverage** **(Current LOS), *n***	**Gap of SU beds for 90% coverage** **(Ideal LOS), *n***
Abruzzo	2,696	2,489 (92.3)	39 (26–56; 155)	–	–
Alto-Adige	979	730 (74.5)	53 (40–66; 104)	2	2
Basilicata	1,027	417 (40.6)	42 (33–51; 93)	10	5
Calabria	2,997	1,616 (53.9)	40 (28–61; 139)	20	11
Campania	7,759	6,186 (79.7)	36 (23–57; 135)	12	8
Emilia-Romagna	9,734	6,440 (66.2)	28 (20–46; 126)	32	23
Friuli-Venezia Giulia	2,767	2,178 (78.7)	27 (19–37; 88)	4	3
Lazio	10,327	5,656 (54.8)	24 (16–40; 133)	59	36
Liguria	4,163	3,583 (86.1)	35 (23–46; 86)	2	2
Lombardia	16,530	15,556 (94.1)	26 (18–38; 130)	–	–
Marche	2,934	2,272 (77.4)	37 (23–57; 123)	5	4
Molise	470	313 (66.6)	51 (35–66; 107)	2	1
Piemonte	8,497	7,430 (87.4)	26 (18–37; 139)	3	2
Puglia	5,959	3,846 (64.5)	25 (18–32; 90)	28	15
Sardegna	2,872	1,669 (58.1)	58 (38–73; 173)	18	9
Sicilia	8,216	4,866 (59.2)	34 (23–48; 175)	41	25
Toscana	8,092	7,872 (97.3)	42 (27–64; 151)	–	–
Trentino	1,038	730 (70.4)	41 (31–50; 110)	2	2
Umbria	2,058	1,877 (91.2)	33 (22–46; 129)	–	–
Valle D’Aosta	257	255 (99.4)	47 (35–60; 121)	–	–
Veneto	9,381	7,458 (79.5)	26 (18–37; 106)	16	10

The current LOS is obtained from the mean SU LOS within the region. The ideal LOS corresponds to 3.65 days, which results in approximately 100 stroke patients per SU bed per year.

Abbreviations: LOS = length of stay; max = maximum; SU = stroke unit.

Figure 3 depicts the map of capacity-constrained SU coverage in Italy. The SU bed gap was calculated based on the current median LOS of each region, resulting in a national deficit of 255 beds. To obtain a comparison, we repeated the calculation considering an ideal LOS of 3.65 days, which resulted in a lower national gap of 158 SU beds. Detailed regional maps are available in Figures S22–S42.

**Figure 3:**
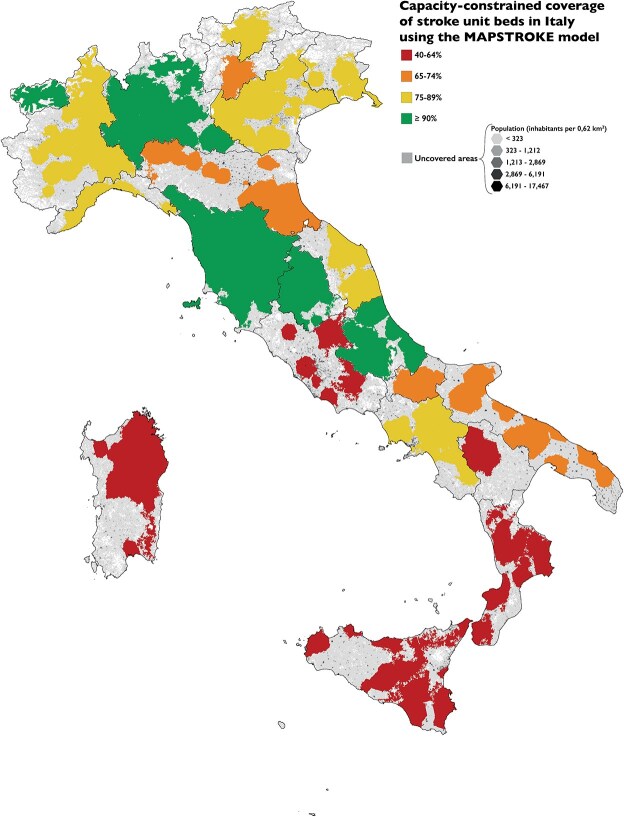
Capacity-constrained stroke unit (SU) coverage in Italy. The catchment areas of each hospital within a region were merged and coloured according to the coverage thresholds for the annual number of strokes. The hexagonal grid is painted according to the population density, and is visible in uncovered areas. Darker hexagons represent regions with a higher number of stroke cases.

## Discussion

In this study, we report the first application of the MAPSTROKE algorithm in a European country. Our data show that the Italian stroke network, overall, is sufficiently spread across the country to ensure access to a hospital providing reperfusion treatment within 45 min for more than 90% of the incident AIS. However, 7 regions did not achieve this optimal coverage, and disparities are evident between the areas with the lowest and highest rates (33.9% and 98.4%, respectively). As expected, changing the time threshold to 30 and 60 min reduced and increased coverage, respectively, resulting in less than 80% of the Italian population reaching a reperfusion-capable site within 30 min.

In contrast, the coverage for SU beds nationwide was 79.2% of the annual national stroke incidence. The 90% target was reached in only 5 of 21 regions, while Basilicata reported the lowest coverage, at 40.6%.

We applied the optimisation strategy for the 45-min access to hospitals providing reperfusion treatments to the 7 regions below the 90% coverage by converting 23 PASC into PSC, thereby achieving the target coverage within all regions except Basilicata. All the PASC included in our analysis were equipped with emergency department (ED), neuroimaging and laboratory services, but 23 of them did not provide 24-h service for CT or laboratory. We also included these last centres, predicting an increase in the availability if needed.

Our analysis shows a significant gap in SU beds across several regions, totalling 255 beds to reach the 90% coverage target. Italian regions differ in their health care systems, policies and financial resources, which ultimately increases the risk of inequity across the country.^22,23^

Stakeholders should also consider strategies to reduce the LOS in the SU, as this increases the turnover of SU beds. Timely SU care, together with early supported discharge, is cost-effective considering years of life saved.^24^ Other strategies to reduce LOS within the SU include transferring patients to general medical wards or to in-hospital early rehabilitation units. The latter model is currently implemented in several European countries; however, these approaches must be carefully evaluated to ensure that reducing SU LOS does not compromise access to dedicated stroke care and maintains alignment with the SAP-E goals.^25^ Future perspectives include the application of the MAPSTROKE algorithm to post-acute phases of stroke management, and in particular, to test the capacity of rehabilitation networks, which might also provide a more comprehensive perspective about the efficiency of stroke care services in Italy.

Although cumulative coverage for access to hospitals providing any reperfusion treatment is high, our model estimated that only 75.7% of ischemic stroke patients have access to a hospital performing EVT within 45 min. Furthermore, 13 out of 21 regions have EVT coverage within 45 min, below the national average. Two regions lack any CSCs, while others, such as Lazio, have most CSCs concentrated in urban areas. All candidate patients should have rapid access to this treatment, which improves the odds of good clinical outcomes.^1,26^

Since the Italian healthcare system is regionalised, providing care across regional borders can be challenging, although it could benefit underserved areas closer to centres in contiguous regions. A nationally coordinated stroke network might help address this issue. The Italian scenario appears comparable to the German federal regional model, with homogeneous access to reperfusion-capable hospitals but high regional disparities in SU beds per incident stroke case, and similar rates of stroke cases reaching a hospital within 30 min (95%).^27^ The Norwegian stroke network provides uniform access to SU beds and currently has an excess of beds, although projections anticipate increased demand.^28^ Comparisons across European networks should account for differences in geography, population density and socioeconomic constraints. Furthermore, although the ESO has defined European standards and certification criteria for SU,^29^ definitions and requirements still vary significantly across countries.

This study has limitations. The main concern is potential survey-collection bias; however, it remains the most reliable source of data on stroke network efficiency in Italy. As the study was based solely on road transport times, applicability is limited in regions with poor road infrastructure or non-contiguous terrain, such as mountainous or island areas. Stroke incidence data were available only at the regional level and not adjusted for intraregional variability, potentially leading to ecological bias. To address the lack of city-level data, we projected regional incidence onto a fine-scale demographic grid. We integrated a hexagonal grid model based on the demographic density grid from the Kontur Dataset, thereby improving the approximation of intra-regional variability.

Furthermore, patients transferred for EVT from a PSC to a CSC are likely to be hospitalised in the CSC SU, increasing the load on CSC beds and decreasing that of PSCs. Thus, SU bed coverage may be underestimated for PSCs and overestimated for CSCs. Due to the lack of accurate data on occupancy rates/overcrowding and referral flows, these factors were not included; however, in most Italian regions, patients transferred to CSCs may be returned to PSCs within 24 h of EVT, thereby mitigating overcrowding. Finally, the parameters used to calculate coverage and gaps, although primarily based on expert opinion, were extracted from the best available literature.

Our study also has notable strengths. The broad range of data sources provides a comprehensive overview of stroke treatment in Italy, and we used real-world data to develop the algorithm, increasing its relevance for decision-makers. Lastly, the algorithm can be continuously updated to reflect changes in stroke incidence and infrastructure, and future work should verify the cost-effectiveness of this implementation model for access to reperfusion-capable hospitals and SU beds, as well as extend it to other aspects of stroke care, such as rehabilitation.

## Conclusion

The MAPSTROKE project provided a national geospatial analysis of stroke care in Italy, revealing overall adequate access to hospitals providing reperfusion treatment but marked disparities in SU capacity. Strengthening SU resources through a nationally coordinated strategy, aligned with the Stroke Action Plan for Europe, is essential to ensure equitable and timely care across the country. MAPSTROKE can support data-driven planning and continuous monitoring of stroke care coverage.

## Supplementary Material

Supplementary_methods

Supplementary_Figures

Supplementary_Tables_Rev1

## Data Availability

Data used for this study are available from the authors upon reasonable request.
