# Supplementary material for: The MAPSTROKE analysis of the access to stroke reperfusion treatment and stroke units in Italy
Source: Eur Stroke J. 2026 Feb 9;11(2):aakaf030. doi: 10.1093/esj/aakaf030 (PMC12884559; doi:10.1093/esj/aakaf030)
Supplement: Supplementary_methods [file supplementary_methods.pdf]

Supplementary Methods

The MAPSTROKE analysis of the access to stroke reperfusion treatment and stroke units in Italy.

Contents

1 Supplementary Methods 1: Analysis 1 - Formulation 2

2 Supplementary Methods 2: Analysis 2 - Formulation and Methods 4

3 Supplementary Methods 3: Software Environment and Tools for Geospatial Analysis 8

1 Supplementary Methods 1: Analysis 1 - Formulation

We formulate the problem of designating candidate hospitals as stroke centers as a Partial Set Covering Location Problem (PSCLP) (Daskin and Owen, 1999). In contrast to classical set covering models, which require full coverage of all demand, the PSCLP aims to identify the minimum number of facilities required to cover at least a specified aggregate demand level T. This framework is appropriate for regional stroke planning, where achieving full geographic coverage is often infeasible due to resource constraints. A demand node i is considered covered by a hospital j when the travel time between them does not exceed a predefined maximum threshold. The resulting Integer Linear Programming (ILP) formulation is presented below.

Sets and Indices

- I: Set of demand nodes, indexed by i.
- J: Set of candidate and existing hospitals, indexed by j.
- $J_{\text{pot}} \subseteq J$: Set of potential (Type 1) hospitals.
- $J_{\text{asrh}} \subseteq J$: Set of ASRH (Type 2) hospitals.
- $J_{\text{sc}} \subseteq J$: Set of SC (Types 3 and 4) hospitals.
- $N_i \subseteq J$: Set of hospitals that can cover demand node i (i.e., those within the travel-time threshold).

Parameters

- d_i: Stroke incidence (demand) associated with demand node i.
- T: Minimum required aggregate demand coverage (target coverage).

Decision Variables

- $y_j \in \{0, 1\}$: Equals 1 if hospital j is selected as a stroke center, and 0 otherwise.
- $z_i \in \{0, 1\}$: Equals 1 if demand node i is covered by at least one selected hospital, and 0 otherwise.

Objective Function

The objective is to minimize the number of newly designated stroke centers, while existing centers remain fixed:

$$\min Z = \sum_{j \in J_{\text{pot}}} y_j.$$

Constraints

1. Mandatory Opening of Existing Stroke Centers Existing stroke centers (Comprehensive, Primary, and Acute-Stroke Ready Hospitals) must remain open:

$$y_j = 1, \quad \forall j \in J_{\text{sc}} \cup J_{\text{asrh}}.$$

2. Coverage Enforcement A demand node may be marked as covered only if at least one hospital capable of reaching it within the allowable travel time is open:

$$\sum_{j \in N_i} y_j \geq z_i, \quad \forall i \in I.$$

3. Demand Coverage Requirement The total demand associated with covered nodes must meet or exceed the regional coverage target:

$$\sum_{i \in I} d_i \, z_i \geq T.$$

4. Integrality Conditions

$$y_j, \, z_i \in \{0, 1\}, \quad \forall j \in J, \, \forall i \in I.$$

2 Supplementary Methods 2: Analysis 2 - Formulation and Methods

In Analysis 2, we formulate the definition of catchment areas for stroke centers as a Fixed-Center Districting Problem (Kalcsics and Ríos-Mercado, 2019) to define catchment areas of the stroke centers. Our model fixes the centers of each district on a stroke center location. The objective function comprises three components. The first is a classical p-median term (Ahmadi-Javid et al., 2017), designed to minimize the total weighted travel distance for patients. The second term imposes penalties on stroke centers that fail to achieve a target occupancy rate, calculated as the ratio of assigned patient demand to the hospital's capacity. Finally, the third component serves as a soft compactness penalty. It minimizes the number of *cut edges*, adjacency connections between demand nodes assigned to different districts, thereby promoting the formation of compact and contiguous districts (Validi and Buchanan, 2022). While ensuring strict geographic contiguity via flow-based formulations (Shirabe, 2009) is theoretically ideal, such methods prove computationally intractable at the scale of our instances. Thus, we rely on the cut-edge penalty to approximate contiguous solutions. The complete ILP formulation is provided below.

Sets

- I: Set of demand nodes, indexed by i.
- K: Set of districts (indexed by hospitals), indexed by k.
- $A \subseteq I \times I$: Set of directed adjacency arcs (i, j) between neighboring demand units.
- $F \subseteq I \times K$: Feasible assignment pairs (i, k) for which the travel time from i to k does not exceed the time limit.

Parameters

- d_i: Demand (incidence) associated with node i.
- c_k: Service capacity of district k.
- τ_i^k: Travel time from demand node i to district center k.
- λ: Penalty weight for unsatisfied saturation.
- β: Penalty weight for boundary disagreement.
- α: Saturation tolerance parameter; each district must serve at least $c_k(1 - \alpha)$.
- $\text{center}(k)$: Demand node designated as the center of district k.

Decision Variables

- $x_{ik} \in \{0,1\}$: Equals 1 if demand node i is assigned to district k.
- $s_k \geq 0$: Slack variable representing unsatisfied coverage of district k.
- $z_{ijk} \in [0,1]$: Soft-boundary variable approximating $|x_{ik} - x_{jk}|$ for adjacent nodes $(i,j) \in A$.

Objective Function

$$\min \underbrace{\sum_{(i,k)\in F} \tau_i^k \, d_i \, x_{ik}}_{\text{travel cost}} + \underbrace{\lambda \sum_{k \in K} s_k}_{\text{saturation penalty}} + \underbrace{\beta \sum_{(i,j)\in A} \sum_{k\in K} z_{ijk}}_{\text{boundary penalty}} . \tag{1}$$

Constraints

1. Assignment constraints (non-centers). Each non-center demand unit may be assigned to at most one feasible district:

$$\sum_{\substack{k \in K \\ (i,k)\in F}} x_{ik} \leq 1, \qquad \forall\, i \in I \setminus \{\text{center}(k) : k \in K\}. \tag{2}$$

2. Center assignment constraints. Each district center must be assigned to its own district:

$$x_{\text{center}(k),\,k} = 1, \qquad \forall k \in K, \tag{3}$$

$$x_{\text{center}(k),\,k'} = 0, \qquad \forall k \in K,\; k' \in K \setminus \{k\}. \tag{4}$$

3. Hard capacity constraints.

$$\sum_{\substack{i \in I \\ (i,k)\in F}} d_i \, x_{ik} \;\leq\; c_k, \qquad \forall k \in K. \tag{5}$$

4. Soft saturation constraints.

$$\sum_{\substack{i \in I \\ (i,k)\in F}} d_i \, x_{ik} \;+\; s_k \;\geq\; c_k(1-\alpha), \qquad \forall k \in K. \tag{6}$$

5. Soft contiguity. For each adjacency arc $(i,j) \in A$ and each district k:

$$z_{ijk} \geq x_{ik} - x_{jk}, \tag{7}$$

$$z_{ijk} \geq x_{jk} - x_{ik}. \tag{8}$$

6. Variable domains.

$$x_{ik} \in \{0,1\}, \quad s_k \geq 0, \quad 0 \leq z_{ijk} \leq 1.$$

Two-Stage Algorithm

While the ILP model yields well-balanced assignments with satisfactory occupancy levels, it may still produce districts containing disconnected components. To correct these artifacts, we adopt a two-stage solution procedure (Algorithm 1) that applies a destroy-and-repair style post-processing heuristic to enforce contiguity and improve occupancy levels. In the first stage, we solve the ILP formulation described in Section 2 to obtain an initial districting configuration. In the second stage, we apply a post-processing heuristic that removes disconnected fragments, enforces geographic contiguity, and improves occupancy utilization where possible. The refinement procedure is detailed in Algorithm 2.

Algorithm 1 Two-Stage Districting Procedure

Input: Instance data $(I, K, A, F, d_i, c_k, \tau_i^k)$
Output: Final district assignment $\tilde{x}$

Stage 1: Solve ILP
Compute an optimal solution $x^\star$ to the ILP formulation in Section 2.

Stage 2: Post-Processing Heuristic
Apply the refinement heuristic (Algorithm 2) to $x^\star$.
Let $\tilde{x}$ denote the refined assignment.

return $\tilde{x}$

Algorithm 2 Post-processing Refinement of ILP Districting Solution

```
Input: Initial ILP assignment x, capacities c_k, adjacency structure, district centers.
Output: Refined districting solution x̃.

Step 1: Initialize.
Start from the ILP solution and set x̃ ← x.

Step 2: Remove disconnected components.
for each district k do
    Identify all connected components of nodes assigned to k.
    Retain only the component containing the center of k.
    Reassign all disconnected components to an unassigned pool.
end for

Step 3: District expansion.
for each district k do
    while k is not saturated and expansion is feasible do
        Add neighboring feasible nodes that do not violate capacity.
        Mark district as blocked if no feasible expansion remains.
    end while
end for

Step 4: Redistribution from under-occupied districts.
for each district k with remaining capacity do
    for each neighboring district h do
        Identify candidate nodes i on the boundary of h.
        if assigning i to k does not exceed c_k and preserves contiguity of h then
            Reassign node i from h to k.
        end if
    end for
end for

Step 5: Final expansion.
for each district k do
    while k not at full capacity and expansion possible do
        Expand into feasible adjacency nodes as before.
    end while
end for

return x̃
```

3 Supplementary Methods 3: Software Environment and Tools for Geospatial Analysis

All data processing, geospatial analysis, and optimization modeling were conducted using Python (version 3.10.16). We utilized the `GeoPandas` library for vector-based spatial operations, including spatial indexing and geometric intersections. Network-based travel times and isochrone polygons were retrieved via the OpenRouteService (ORS) API, which was employed to generate driving travel-time isochrones for each candidate facility.

ORS Travel Time Estimation

ORS is an open-source project offering various geospatial services through an API. The project started in 2008 (Neis and Zipf, 2008), and currently is maintained by the Heidelberg Institute for Geoinformation Technology*. Its primary focus is network analysis functions, including route planning, geocoding, isochrone calculations, and distance matrix computations. ORS computations are performed using the OpenStreetMap (OSM) database, the leading collaborative project for producing a freely accessible and editable global map.

ORS can compute travel times for driving, walking, cycling, and wheelchair profiles. For the driving profile, ORS determines the base speed of each OSM segment using a hierarchical procedure. When a segment includes an explicit speed limit in the maxspeed tag, ORS assigns 90% of that value as the base speed. If no speed limit is defined, the base speed is inferred from the segment's highway tag. Table 1 summarizes the default speeds associated with each highway category.

Table 1: Base Speed by Highway Tag

Highway Tag	Base Speed (km/h)
`motorway`	100
`motorway_link`	60
`motorroad`	90
`trunk`	85
`trunk_link`	60
`primary`	65
`primary_link`	50
`secondary`	60
`secondary_link`	50
`tertiary`	50
`tertiary_link`	40
`unclassified`	30
`residential`	30
`living_street`	10
`service`	20
`road`	20
`track`	15

*https://heigit.org/ (visited on 25/11/2025)

The base speeds might be further modified depending on other factors. These factors include the assignment of a based speed depending on the surface type of the way (based on the `surface` tag), speed reduction when driving in residential ways and roundabouts. A more detailed description can be found on the documentation.[†]

References

Ahmadi-Javid, A., Seyedi, P., and Syam, S. S. (2017). A survey of healthcare facility location. *Computers & Operations Research*, 79:223–263.

Daskin, M. S. and Owen, S. H. (1999). Two new location covering problems: The partial p-center problem and the partial set covering problem. *Geographical Analysis*, 31(3):217–235.

Kalcsics, J. and Ríos-Mercado, R. Z. (2019). *Districting Problems*, pages 705–743. Springer International Publishing, Cham.

Neis, P. and Zipf, A. (2008). Openrouteservice.org is three times "open": Combining open-source, openls and openstreetmaps. pages 248–251.

Shirabe, T. (2009). Districting modeling with exact contiguity constraints. *Environment and Planning B: Planning and Design*, 36(6):1053–1066.

Validi, H. and Buchanan, A. (2022). Political districting to minimize cut edges. *Mathematical Programming Computation*, 14(4):623–672.

[†]https://giscience.github.io/openrouteservice/technical-details/travel-speeds/ - Last visited at 25/11/2025
