# Supplementary material for: The MAPSTROKE analysis of the access to stroke reperfusion treatment and stroke units in Italy
Source: Eur Stroke J. 2026 Feb 9;11(2):aakaf030. doi: 10.1093/esj/aakaf030 (PMC12884559; doi:10.1093/esj/aakaf030)
Supplement: Supplementary_Figures [file supplementary_figures.pdf]

Supplementary Figures

The MAPSTROKE analysis of the access to stroke reperfusion treatment and stroke units in Italy

Contents

1 Supplementary Figures 1 to 21: regional coverage for reperfusion treatment in Italy. 2

2 Supplementary Figures 22 to 42: capacity-constrained coverage of stroke unit beds in Italy 23

1 Supplementary Figures 1 to 21: regional coverage for reperfusion treatment in Italy.

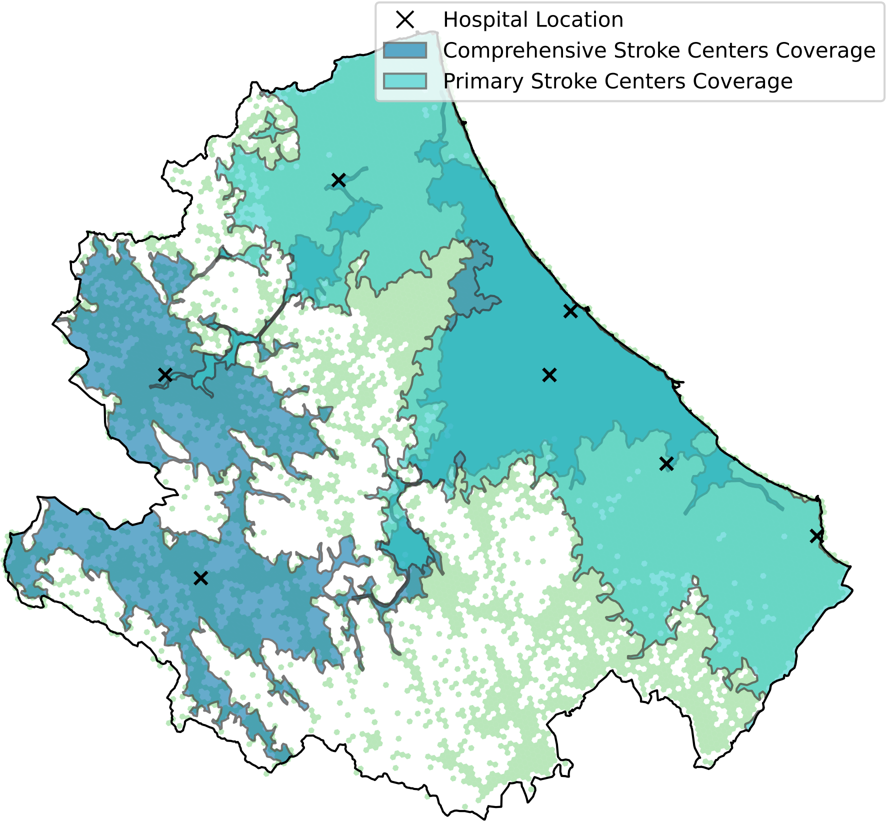

Supplementary Figure 1: Coverage of 94.3% for reperfusion treatment in Abruzzo, including primary (PSCs) and comprehensive stroke centers (CSCs). No optimization was performed, as the current coverage is $\geq$ 90%. Green hexagons in the background show populated regions.

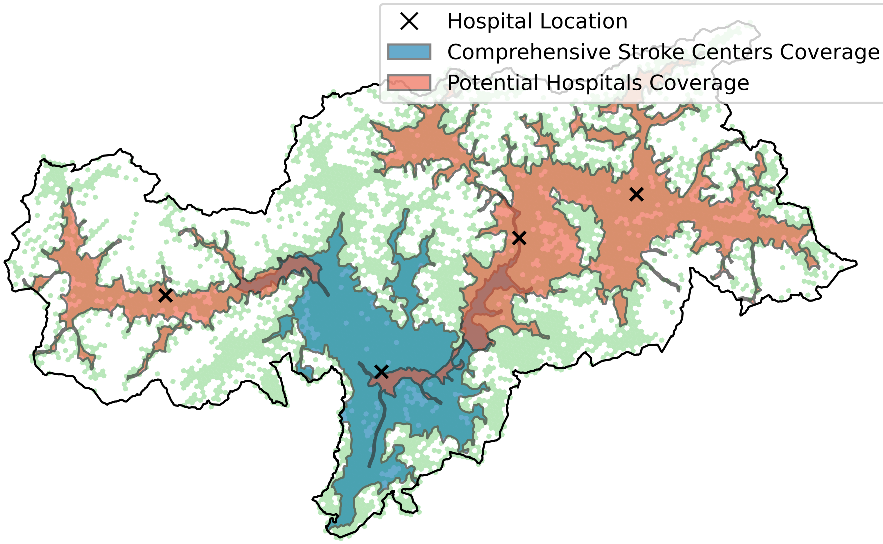

Supplementary Figure 2: Current coverage of 62.8% for reperfusion treatment in Alto-Adige (blue isochrone), that increased to 97.2% after the optimization included 3 potential acute stroke centers (PASCs). Green hexagons in the background show populated regions.

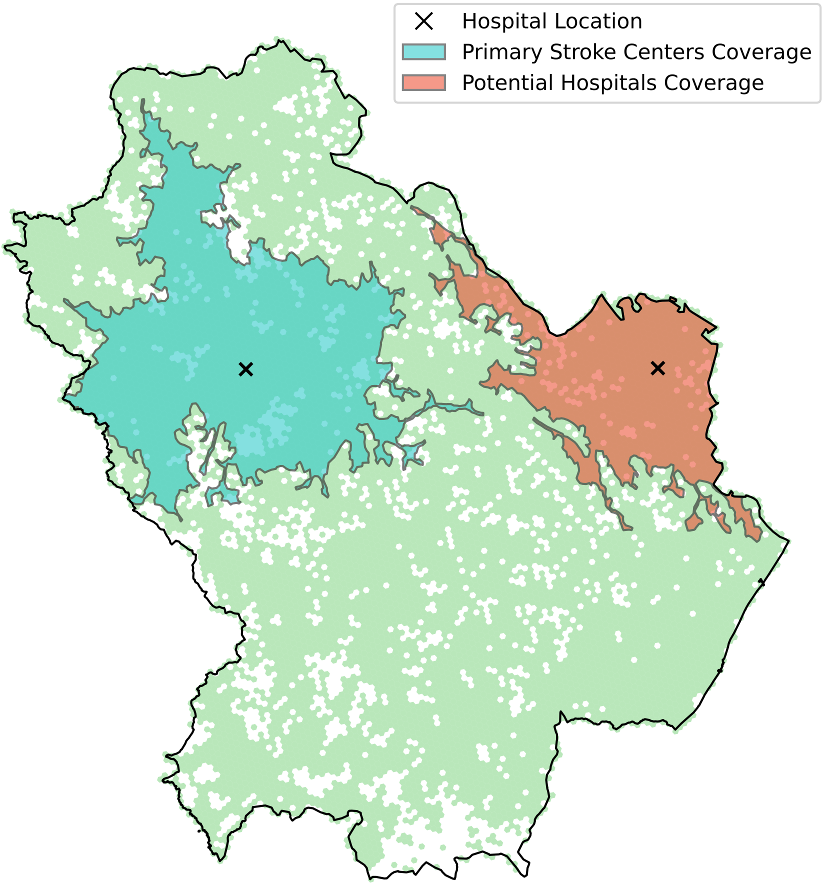

Supplementary Figure 3: Current coverage of 33.9% for reperfusion treatment in Basilicata (green isochrone), that increased to 51.8% after the optimization included 1 potential acute stroke center (PASC). Green hexagons in the background show populated regions.

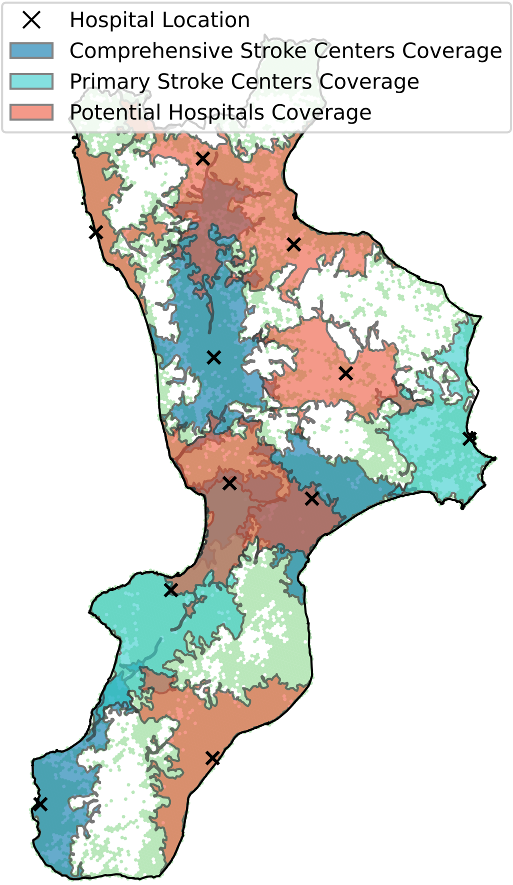

Supplementary Figure 4: Current coverage of 67.2% for reperfusion treatment in Calabria (green and blue isochrones), that increased to 94.5% after the optimization included 7 potential acute stroke centers (PASCs). Green hexagons in the background show populated regions.

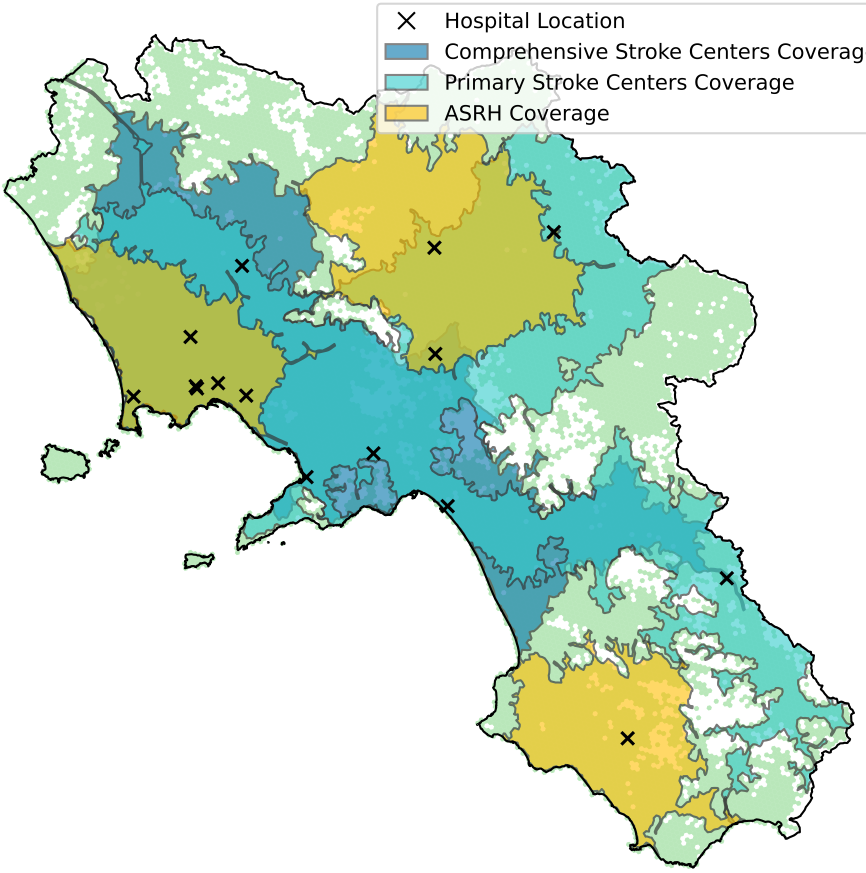

Supplementary Figure 5: Current coverage of 94.9% for reperfusion treatment in Campania, including acute stroke-ready hospitals (ASRHs), primary (PSCs), and comprehensive stroke centers (CSCs). No optimization was performed, as the current coverage is $\geq 90\%$. Green hexagons in the background show populated regions.

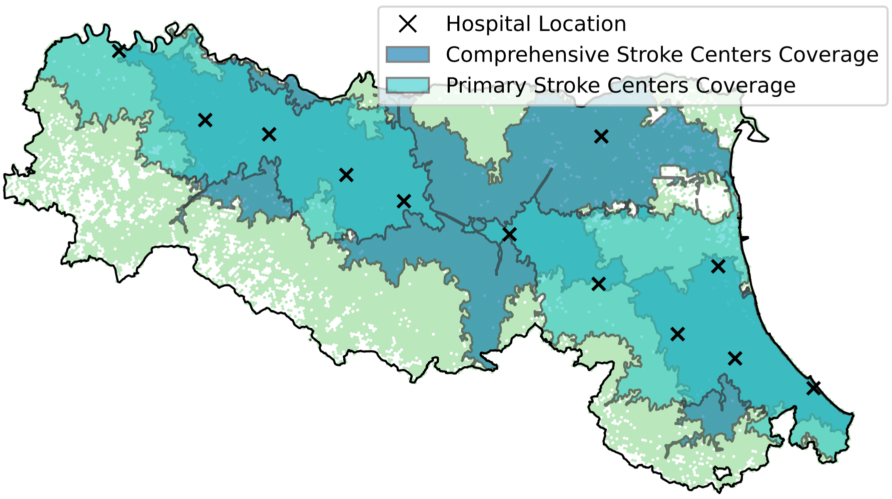

Supplementary Figure 6: Current coverage of 92.7% for reperfusion treatment in Emilia-Romagna, including primary (PSCs) and comprehensive stroke centers (CSCs). No optimization was performed, as the current coverage is $\geq$ 90%. Green hexagons in the background show populated regions.

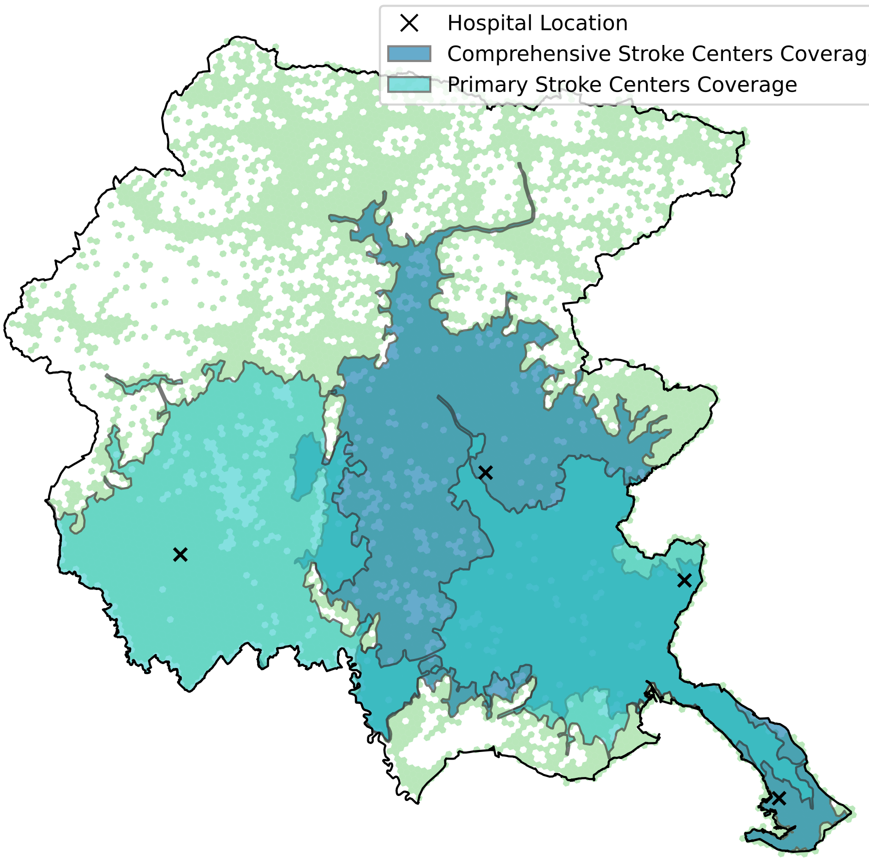

Supplementary Figure 7: Current coverage of 94.9% for reperfusion treatment in Friuli-Venezia Giulia, including primary (PSCs) and comprehensive stroke centers (CSCs). No optimization was performed, as the current coverage is $\geq$ 90%. Green hexagons in the background show populated regions.

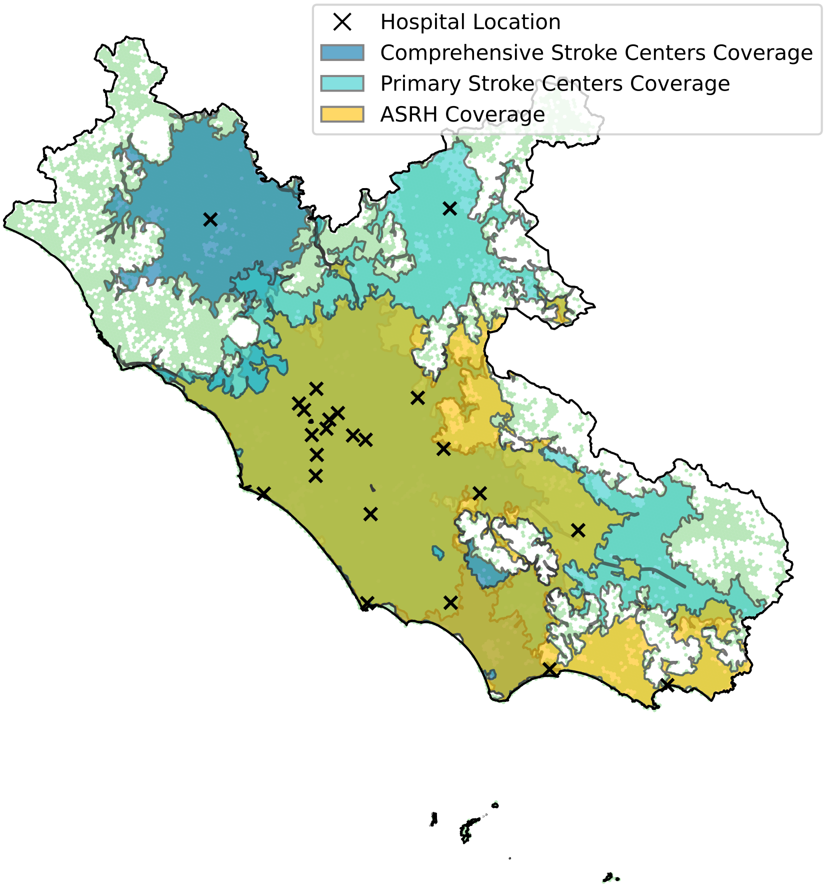

Supplementary Figure 8: Current coverage of 96.6% for reperfusion treatment in Lazio, including acute stroke-ready hospitals (ASRHs), primary (PSCs), and comprehensive stroke centers (CSCs). No optimization was performed, as the current coverage is $\geq 90\%$. Green hexagons in the background show populated regions.

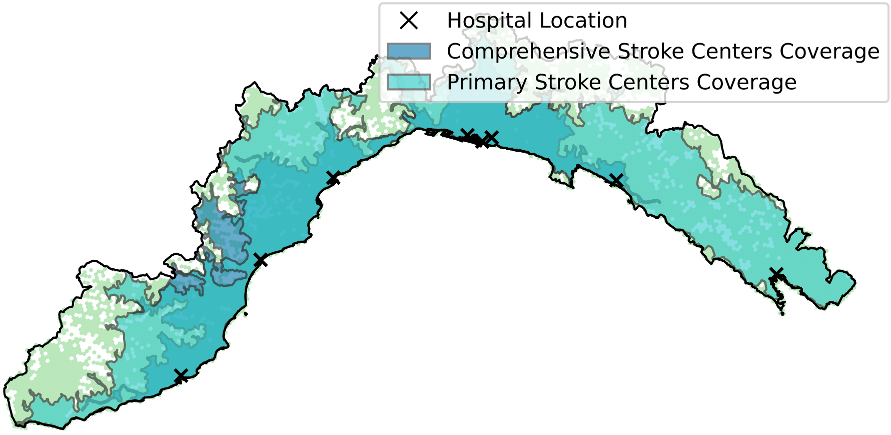

Supplementary Figure 9: Current coverage of 98.4% for reperfusion treatment in Liguria, including primary (PSCs) and comprehensive stroke centers (CSCs). No optimization was performed, as the current coverage is $\geq 90\%$. Green hexagons in the background show populated regions.

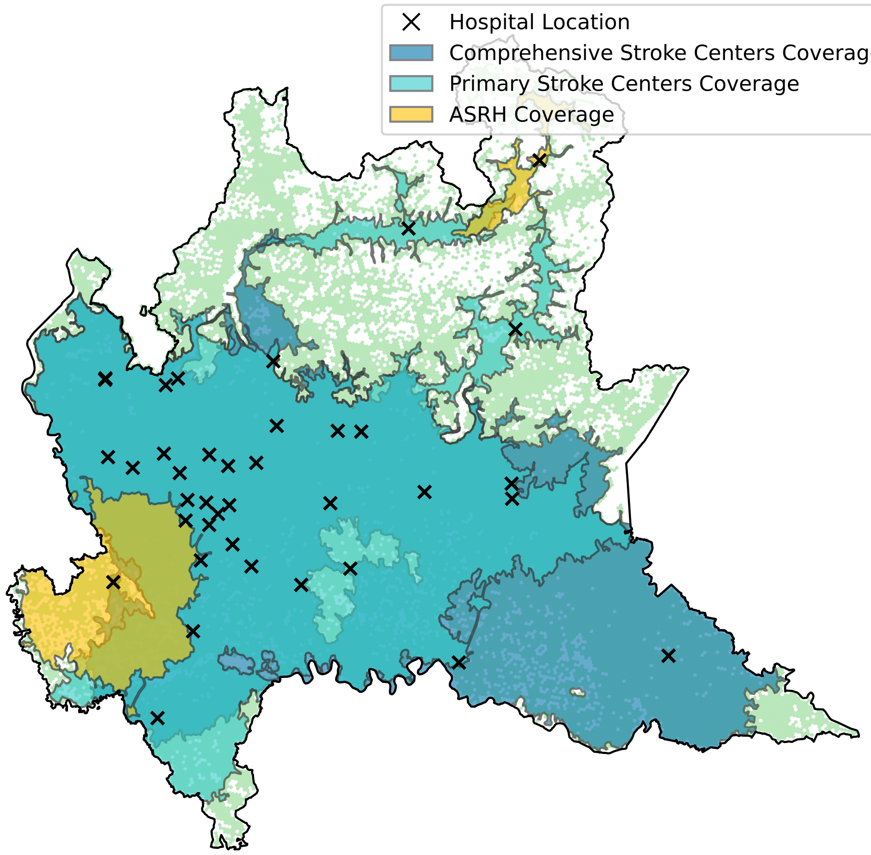

Supplementary Figure 10: Current coverage of 97.9% for reperfusion treatment in Lombardia, including acute stroke-ready hospitals (ASRHs), primary (PSCs), and comprehensive stroke centers (CSCs). No optimization was performed, as the current coverage is $\geq 90\%$. Green hexagons in the background show populated regions.

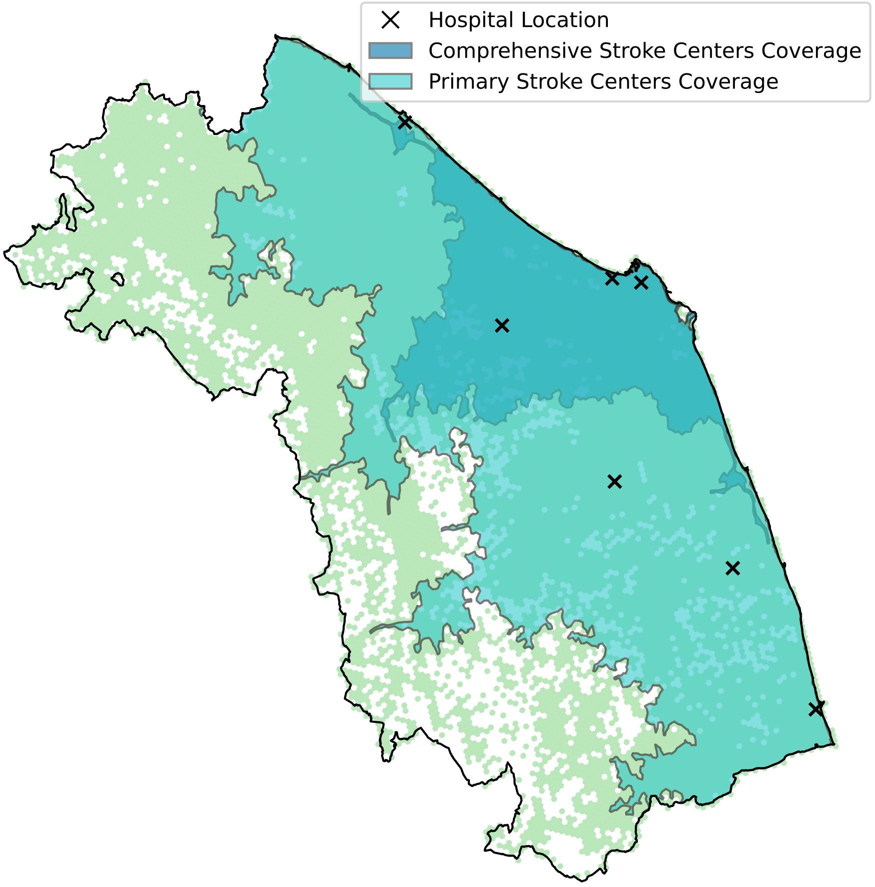

Supplementary Figure 11: Current coverage of 92.2% for reperfusion treatment in Marche, including primary (PSCs) and comprehensive stroke centers (CSCs). No optimization was performed, as the current coverage is $\geq$ 90%. Green hexagons in the background show populated regions.

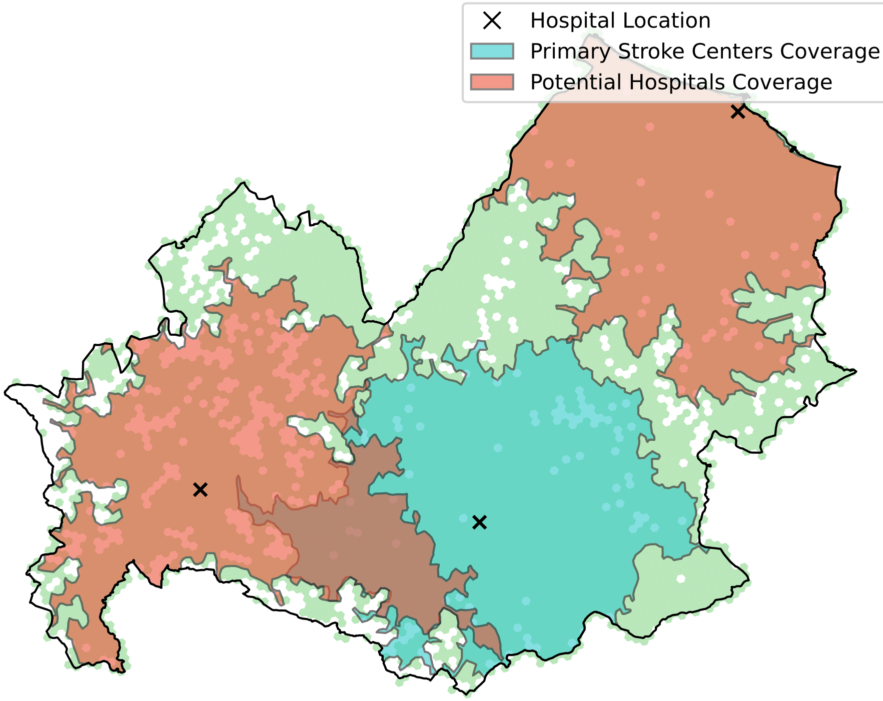

Supplementary Figure 12: Current coverage of 39.7% for reperfusion treatment in Molise (green isochrone), that increased to 92.4% after the optimization included 2 potential acute stroke centers (PASCs). Green hexagons in the background show populated regions.

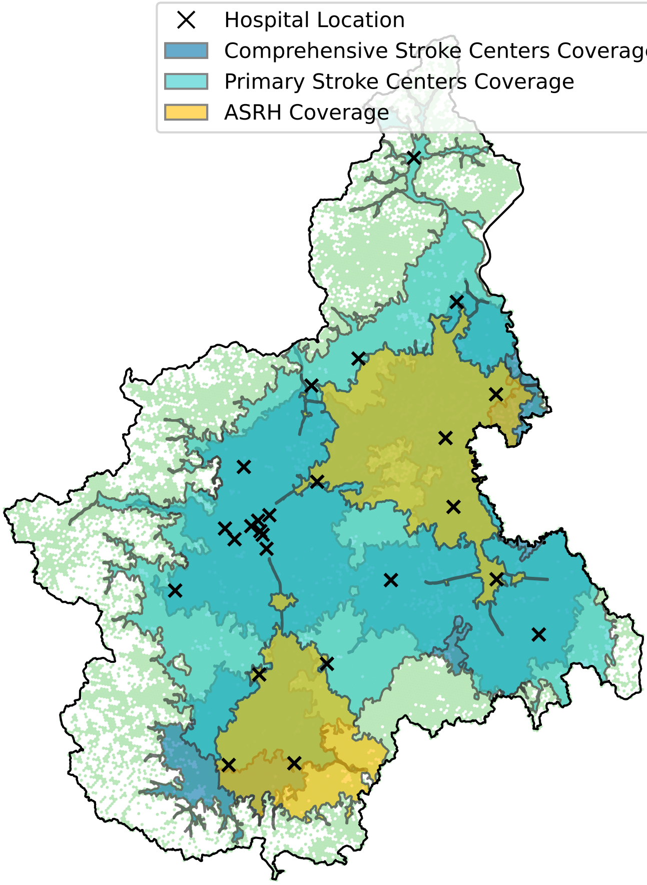

Supplementary Figure 13: Current coverage of 97.7% for reperfusion treatment in Piemonte, including acute stroke-ready hospitals, primary (PSCs), and comprehensive stroke centers (CSCs). No optimization was performed, as the current coverage is $\geq 90\%$. Green hexagons in the background show populated regions.

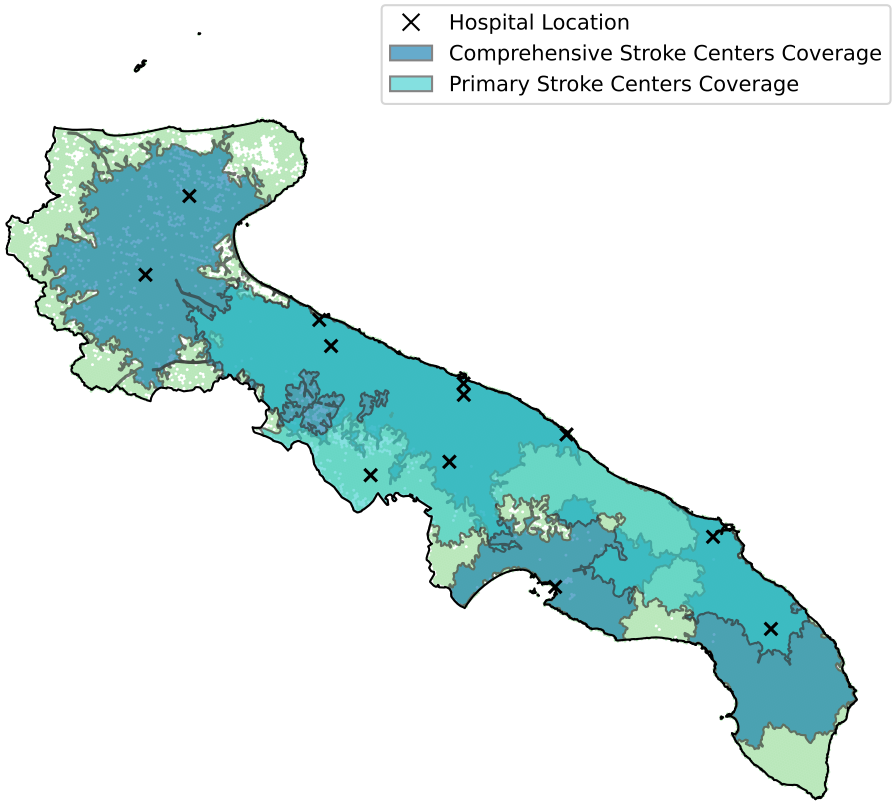

Supplementary Figure 14: Current coverage of 93.3% for reperfusion treatment in Puglia, including primary (PSCs) and comprehensive stroke centers (CSCs). No optimization was performed, as the current coverage is $\geq$ 90%. Green hexagons in the background show populated regions.

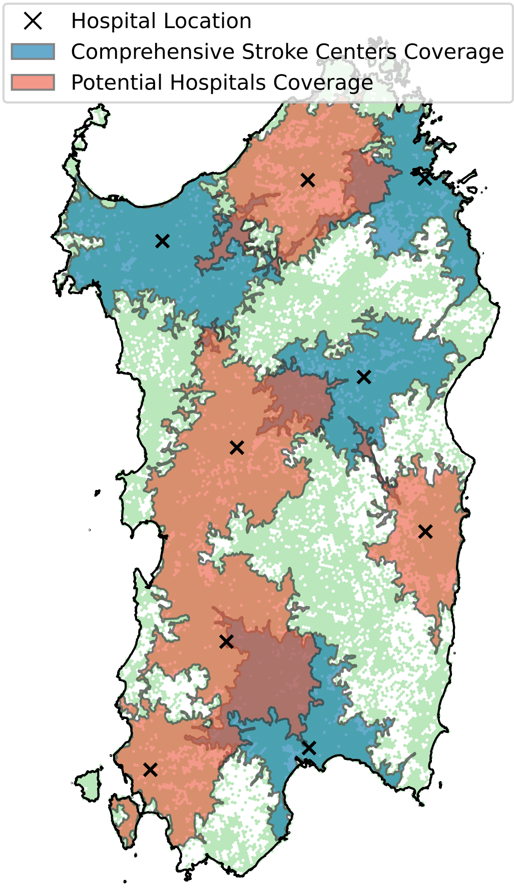

Supplementary Figure 15: Current coverage of 63.5% for reperfusion treatment in Sardegna (blue isochrones), that increased to 97.7% after the optimization included 5 potential acute stroke centers (PASCs). Green hexagons in the background show populated regions.

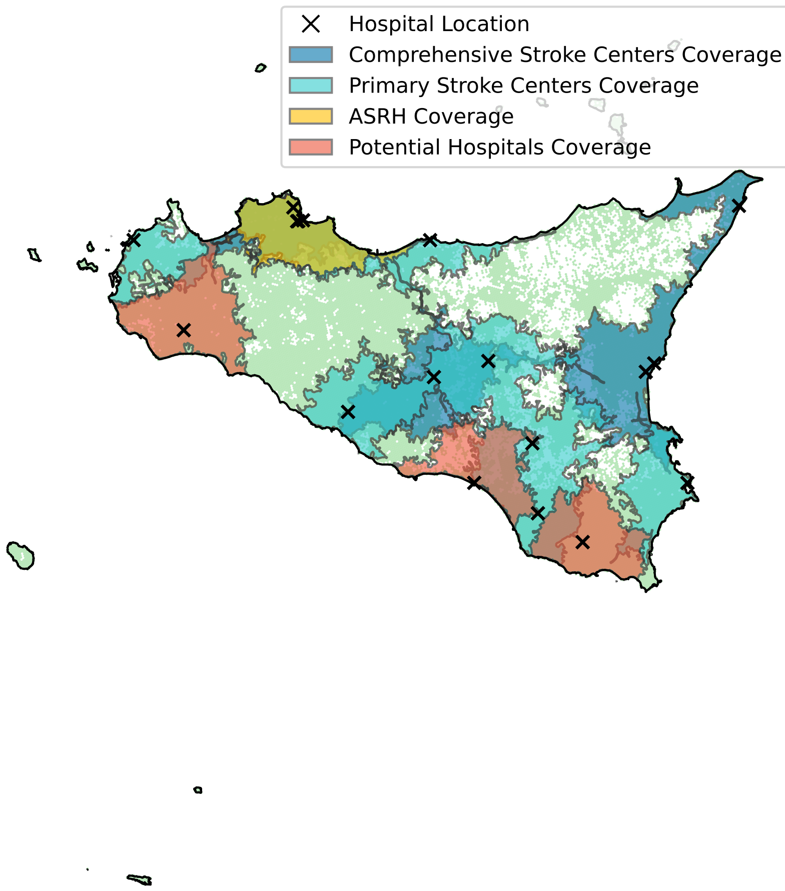

Supplementary Figure 16: Current coverage of 82.1% for reperfusion treatment in Sicilia (yellow, green and blue isochrones), that increased to 97.8% after the optimization included 3 potential acute stroke centers (PASCs). Green hexagons in the background show populated regions.

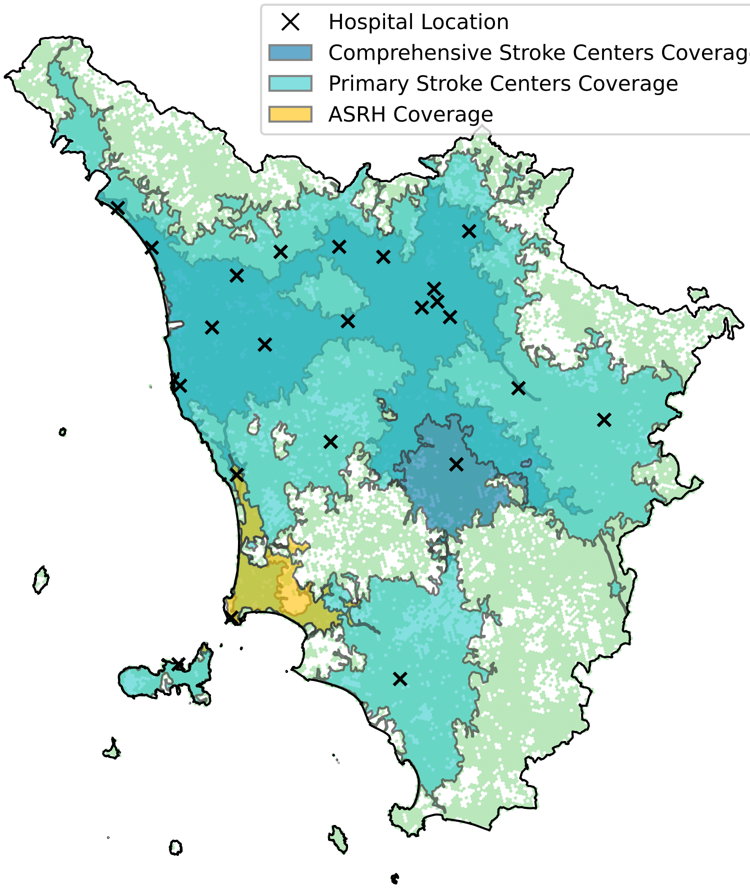

Supplementary Figure 17: Current coverage of 93.8% for reperfusion treatment in Toscana, including acute stroke-ready hospitals (ASRHs), primary (PSCs), and comprehensive stroke centers (CSCs). No optimization was performed, as the current coverage is $\geq 90\%$. Green hexagons in the background show populated regions.

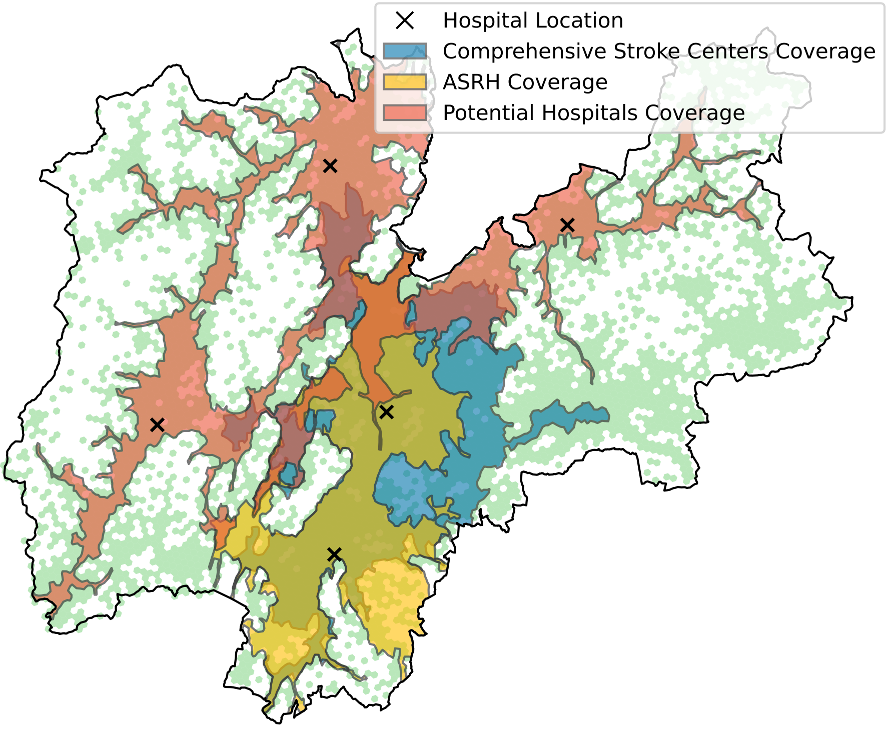

Supplementary Figure 18: Current coverage of 74.2% for reperfusion treatment in Trentino (yellow and blue isochrones), that increased to 97.1% after the optimization included 3 potential acute stroke centers (PASCs). Green hexagons in the background show populated regions.

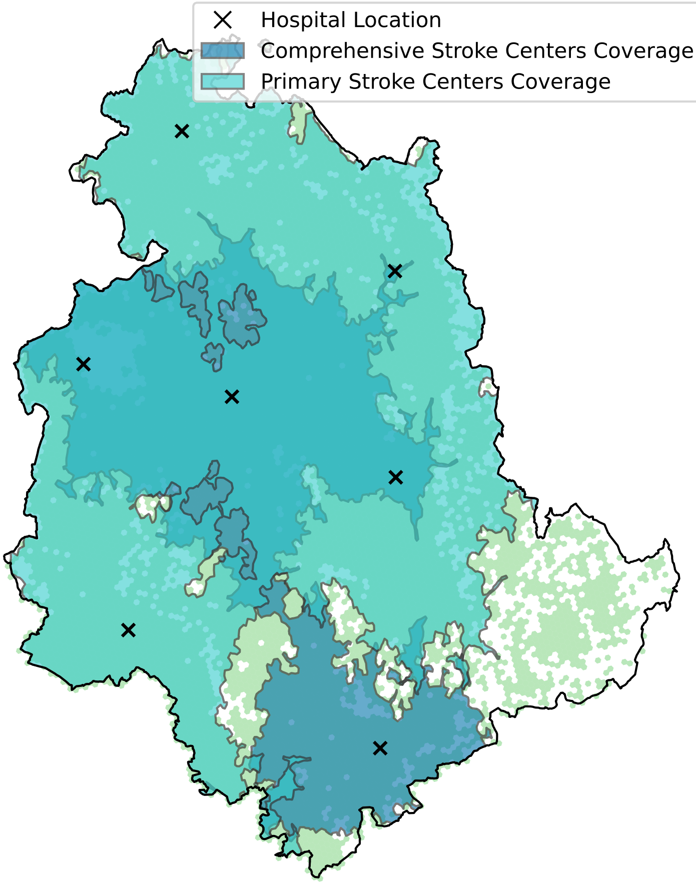

Supplementary Figure 19: Current coverage of 98.2% for reperfusion treatment in Umbria, including primary (PSCs) and comprehensive stroke centers (CSCs). No optimization was performed, as the current coverage is $\geq 90\%$. Green hexagons in the background show populated regions.

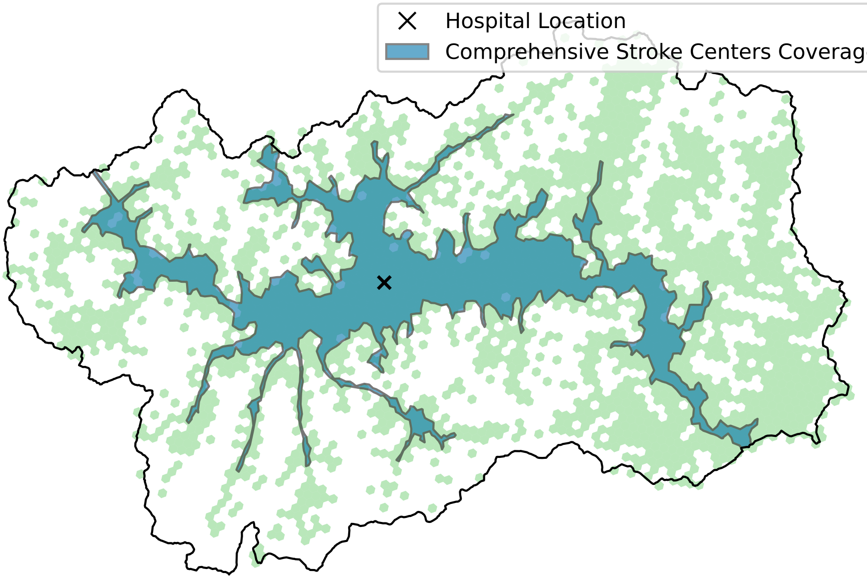

Supplementary Figure 20: Current coverage of 90.2% for reperfusion treatment in Valle D'Aosta, including a comprehensive stroke center (CSC). No optimization was performed, as the current coverage is $\geq$ 90%. Green hexagons in the background show populated regions.

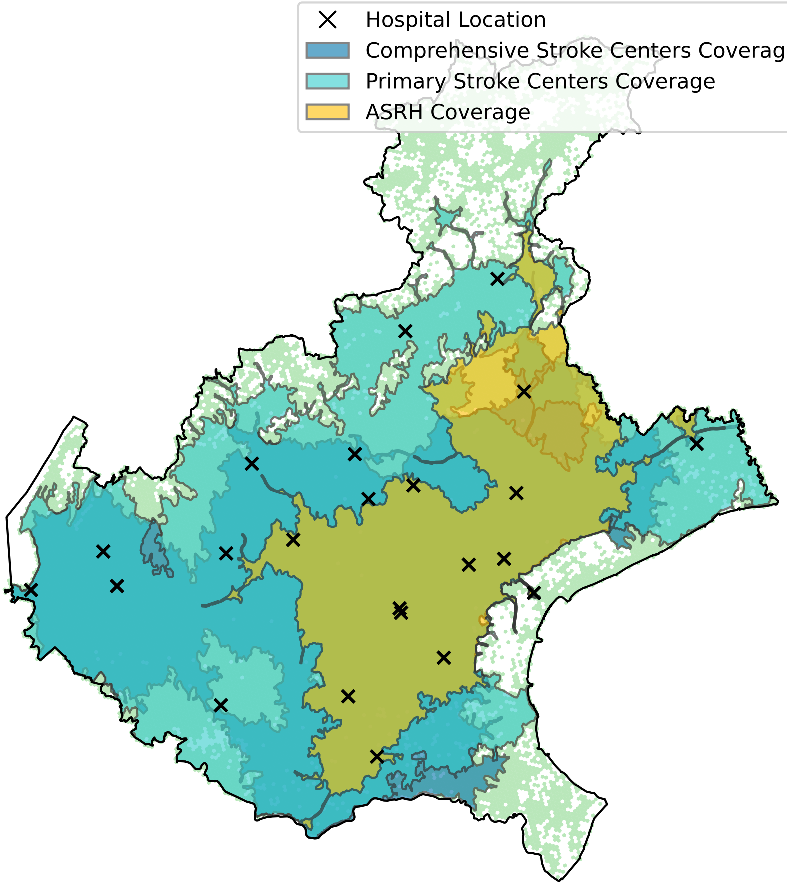

Supplementary Figure 21: Current coverage of 96.1% for reperfusion treatment in Veneto, including acute stroke-ready hospitals (ASRHs), primary (PSCs), and comprehensive stroke centers (CSCs). No optimization was performed, as the current coverage is $\geq$ 90%. Green hexagons in the background show populated regions.

2 Supplementary Figures 22 to 42: capacity-constrained coverage of stroke unit beds in Italy

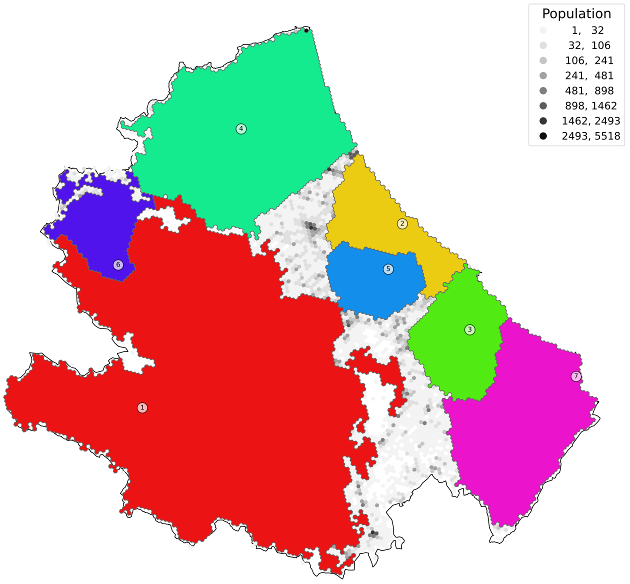

Supplementary Figure 22: Capacity-constrained coverage of stroke unit beds in Abruzzo. Each polygon shows the coverage area of a given hospital . Gray hexagons in the background show populated regions. The legend shows the number of inhabitants according to the shades of gray. 1 SS Filippo e Nicola, 2 Ospedale Civile Spirito Santo, 3 Ospedale F. Renzetti, 4 Ospedale Giuseppe Mazzini, 5 Policlinico SS Annunziata, 6 San Salvatore, 7 San Pio da Pietrelcina

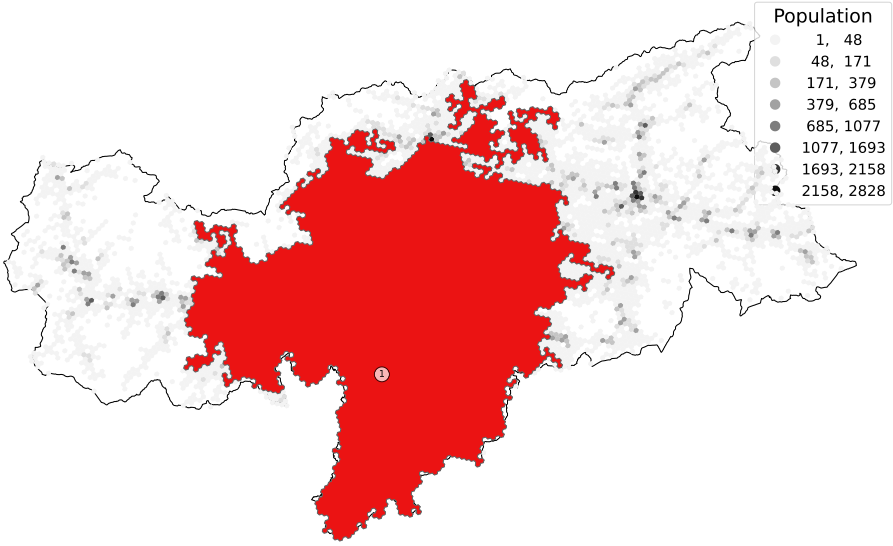

Supplementary Figure 23: Capacity-constrained coverage of stroke unit beds in Alto-Adige. Each polygon shows the coverage area of a given hospital . Gray hexagons in the background show populated regions. The legend shows the number of inhabitants according to the shades of gray. 1 Ospedale centrale di Bolzano

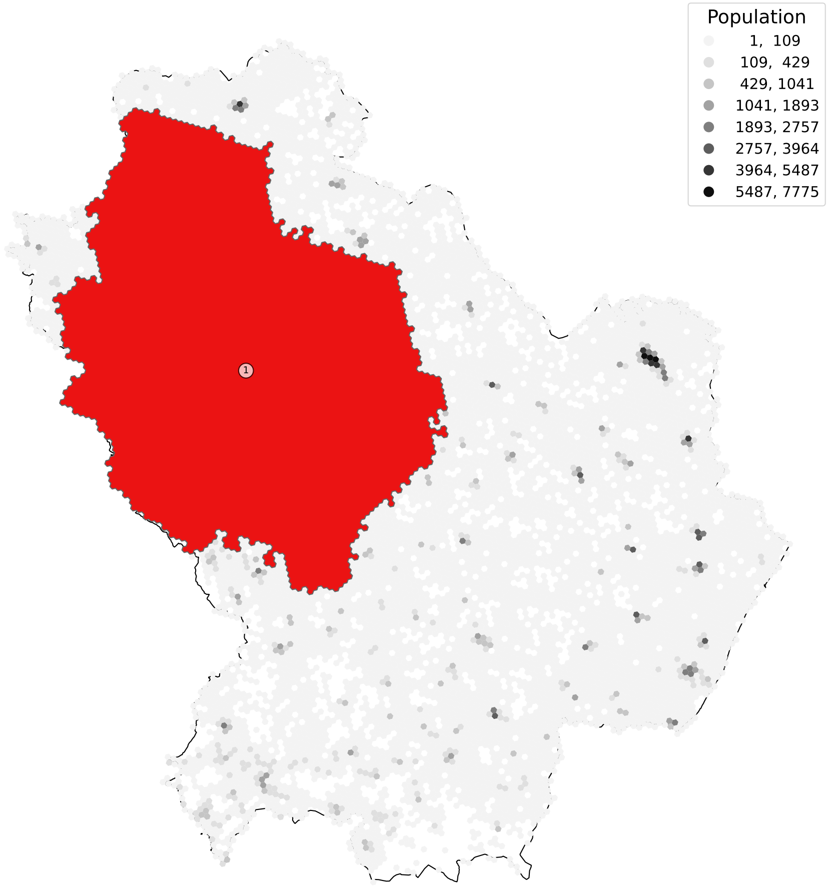

Supplementary Figure 24: Capacity-constrained coverage of stroke unit beds in Basilicata. Each polygon shows the coverage area of a given hospital . Gray hexagons in the background show populated regions. The legend shows the number of inhabitants according to the shades of gray. 1 AOR San Carlo

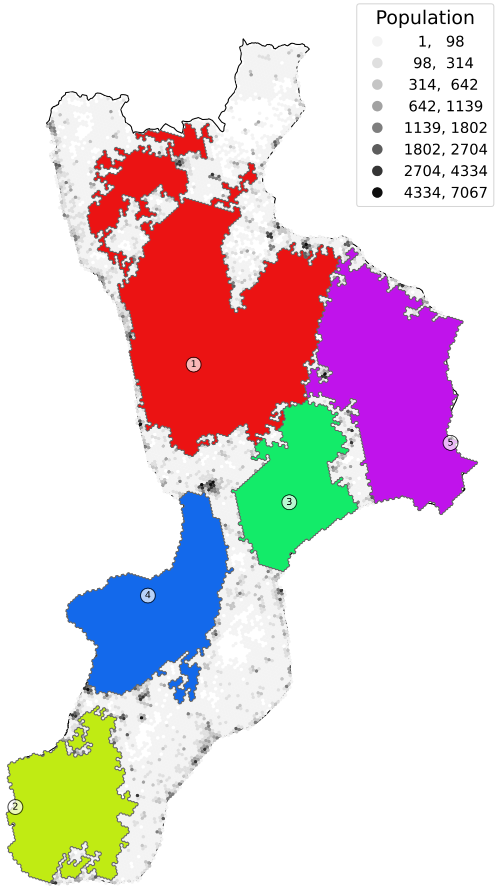

Supplementary Figure 25: Capacity-constrained coverage of stroke unit beds in Calabria. Each polygon shows the coverage area of a given hospital . Gray hexagons in the background show populated regions. The legend shows the number of inhabitants according to the shades of gray. 1 AO SS. Annunziata, 2 AOU Bianchi-Melacrino-Morelli, 3 AO Pugliese Ciaccio, 4 PO Jazzolino, 5 Ospedale civile San Giovanni di Dio

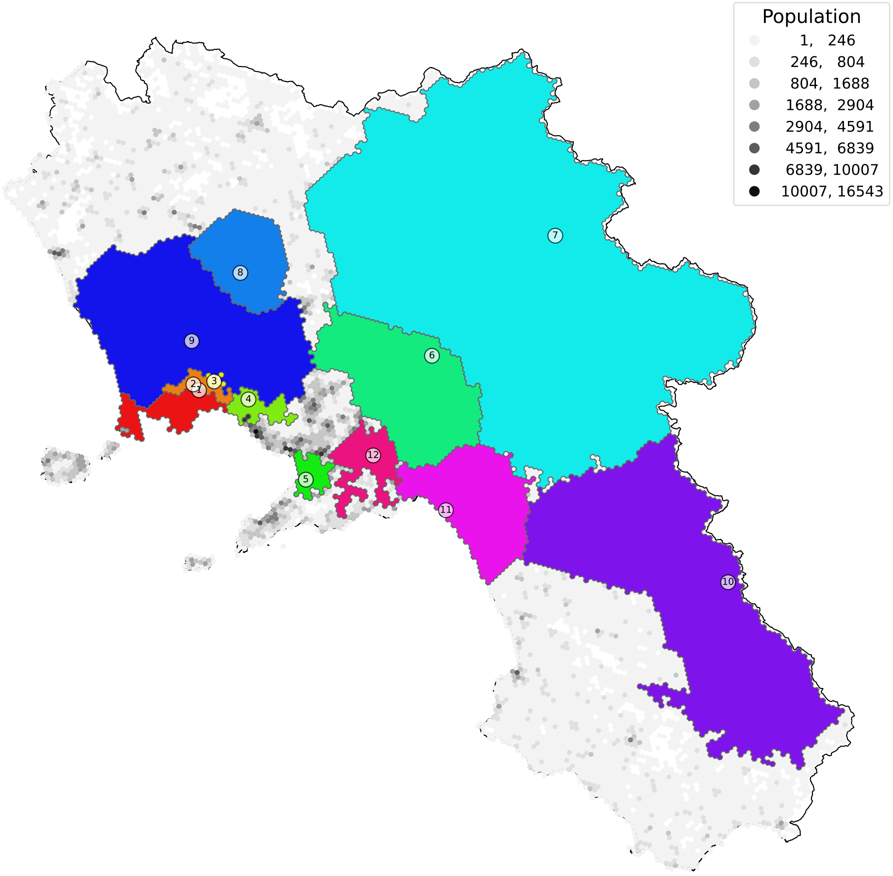

Supplementary Figure 26: Capacity-constrained coverage of stroke unit beds in Campania. Each polygon shows the coverage area of a given hospital . Gray hexagons in the background show populated regions. The legend shows the number of inhabitants according to the shades of gray. 1 AORN Cardarelli, 2 A.O.U. Federico II, 3 P.O. San Giovanni Bosco, 4 PO Ospedale del Mare, 5 PO San Leonardo, 6 AO S.G. Moscati, 7 PO Sant'Ottone Frangipane, 8 AO San Sebastiano, 9 PO San Giuseppe Moscati, 10 PO Luigi Curto, 11 AOU San Giovanni di Dio e Ruggi D'Aragona, 12 PO Umberto I

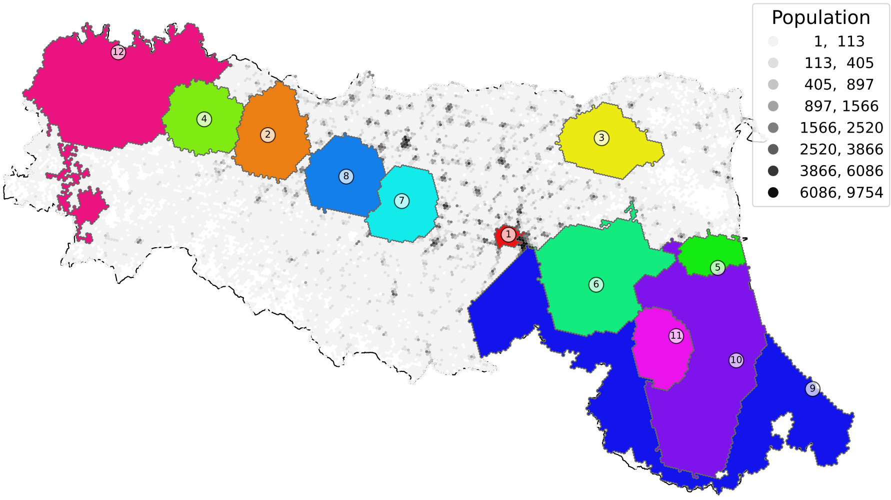

Supplementary Figure 27: Capacity-constrained coverage of stroke unit beds in Emilia-Romagna. Each polygon shows the coverage area of a given hospital . Gray hexagons in the background show populated regions. The legend shows the number of inhabitants according to the shades of gray. 1 Ospedale Maggiore, 2 Maggiore, 3 Ospedale sant'Anna, 4 Ospedale di Fidenza, 5 Santa Maria delle Croci, 6 Santa Maria della Scaletta, 7 Ospedale Civile di Baggiovara, AOU Modena, 8 Arcispedale Santa Maria Nuova, 9 Ospedale Infermi, 10 Ospedale Bufalini, 11 Morgagni-Pierantoni, 12 Ospedale Guglielmo da Saliceto

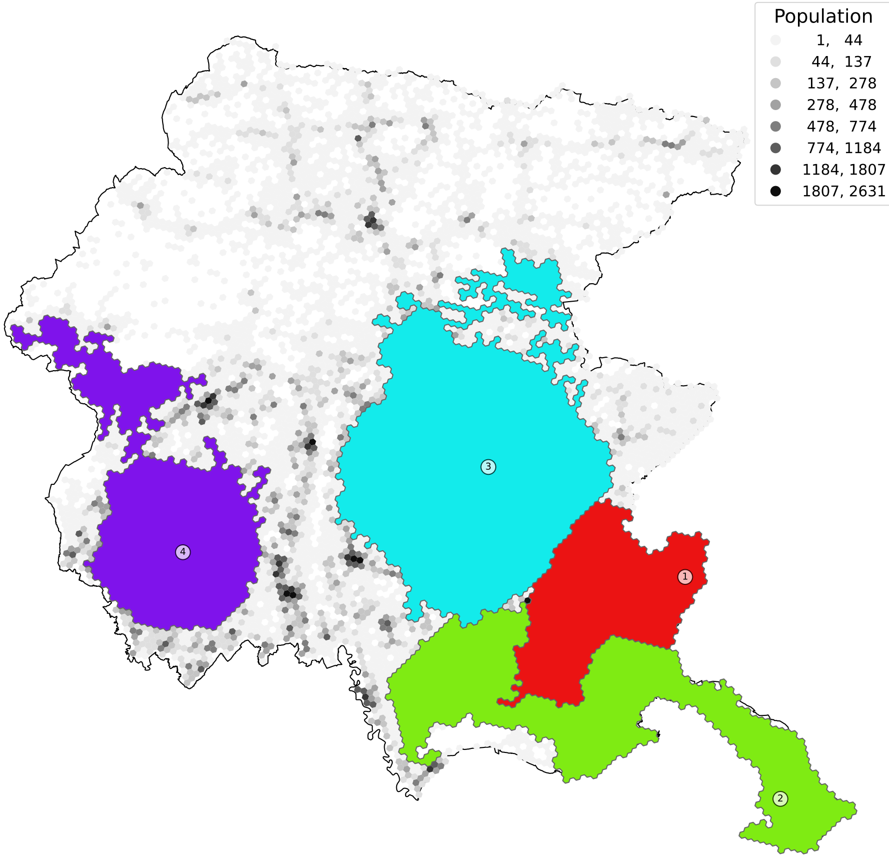

Supplementary Figure 28: Capacity-constrained coverage of stroke unit beds in Friuli-Venezia Giulia. Each polygon shows the coverage area of a given hospital . Gray hexagons in the background show populated regions. The legend shows the number of inhabitants according to the shades of gray. 1 PO Gorizia/Monfalcone, 2 Azienda sanitaria Universitaria giuliano Isontina, 3 Santa Maria della Misericordia, 4 santa Maria degli Angeli

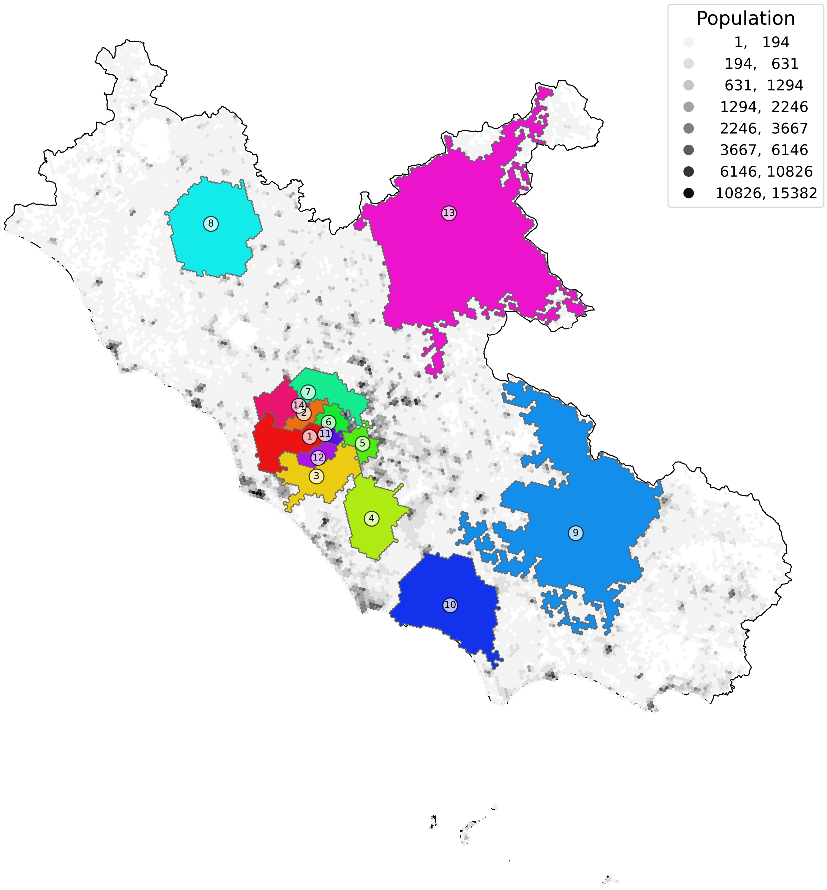

Supplementary Figure 29: Capacity-constrained coverage of stroke unit beds in Lazio. Each polygon shows the coverage area of a given hospital . Gray hexagons in the background show populated regions. The legend shows the number of inhabitants according to the shades of gray. 1 Azienda Ospedaliera San Camillo, 2 Fondazione Policlinico Universitario Agostino Gemelli, 3 Policlinico Campus Bio-Medico, 4 Ospedale dei Castelli, 5 Policlinico Tor Vergata, 6 Policlinico Umberto I, 7 AOU Sant'Andrea, 8 Ospedale Belcolle, 9 Ospedale Fabrizio Spaziani, 10 Ospedale Santa Maria Goretti, 11 Azienda Ospedaliera San Giovanni Addolorata, 12 Ospedale Sant'Eugenio, 13 Ospedale San Camillo de Lellis, 14 Ospedale San Filippo Neri

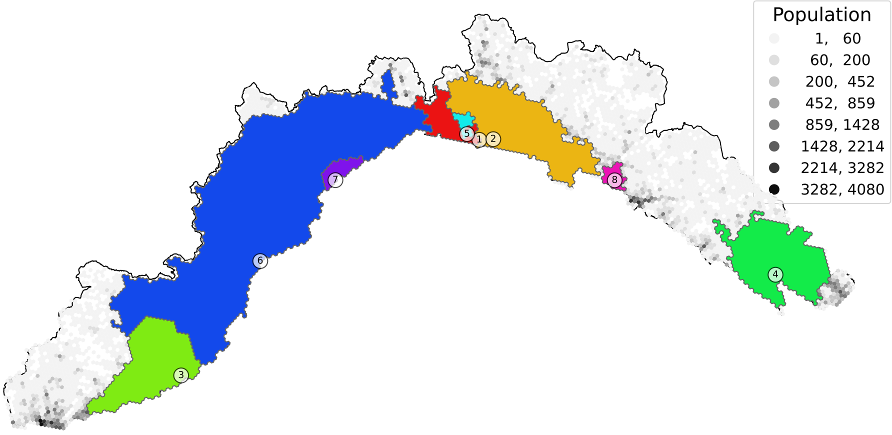

Supplementary Figure 30: Capacity-constrained coverage of stroke unit beds in Liguria. Each polygon shows the coverage area of a given hospital . Gray hexagons in the background show populated regions. The legend shows the number of inhabitants according to the shades of gray. 1 E.O. Ospedali Galliera, 2 University of Genova and San Martino Hospital, 3 Ospedale Imperia, 4 Presidio Ospedaliero Sant'Andrea La Spezia, 5 P.O.M Villa Scassi ASL3 Genova, 6 Ospedale Santa Corona, 7 San Paolo, 8 Ospedale di Lavagna

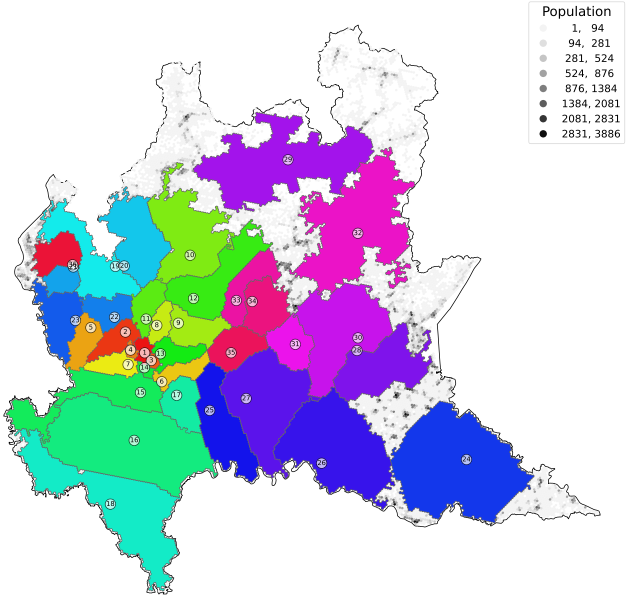

Supplementary Figure 31: Capacity-constrained coverage of stroke unit beds in Lombardia. Each polygon shows the coverage area of a given hospital . Gray hexagons in the background show populated regions. The legend shows the number of inhabitants according to the shades of gray. 1 ASST Grande Ospedale Metropolitano Niguarda, 2 OSPEDALE GUIDO SALVINI GARBAGNATE - ASST Rhodense, 3 ISTITUTO CLINICO CITTA STUDI, 4 Ospedale Sacco, 5 OSPEDALE CIVILE LEGNANO - ASST OVEST MILANESE, 6 IRCCS Policlinico San Donato, 7 OSPEDALE SAN CARLO BORROMEO - ASST Santi Paolo e Carlo, 8 OSPEDALE SAN GERARDO DEI TINTORI- ASST Monza, 9 ASST Brianza - Vimercate, 10 OSPEDALE ALESSANDRO MANZONI - ASST Lecco, 11 OSPEDALE DI DESIO, 12 OSPEDALE SAN LEOPOLDO MANDIC Merate, 13 IRCCS OSPEDALE SAN RAFFAELE, 14 FONDAZIONE IRCCS CA GRANDA OSPEDALE MAGGIORE POLICLINICO, 15 Istituto Clinico Humanitas, 16 IRCCS POLICLINICO SAN MATTEO-MONDINO, 17 OSPEDALE DI VIZZOLO PREDABISSI - ASST Melegnano - Martesana, 18 Ospedale Civile di Voghera - ASST Pavia, 19 Ospedale Sant Anna di Como- ASST Lariana, 20 OSPEDALE VALDUCE - ASST LARIANA, 21 OSPEDALE MORIGGIA - PELASCINI - ASST Sette Laghi, 22 Ospedale di Circolo di Saronno - ASST della Valle Olona, 23 Gallarate- ASST Valle Olona, 24 OSPEDALE CARLO POMA - ASST Mantova, 25 Ospedale Maggiore di Lodi - ASST Lodi, 26 OSPEDALE DI CREMONA - ASST Cremona, 27 OSPEDALE MAGGIORE DI CREMA-ASST Crema, 28 Fondazione Poliambulanza Brescia, 29 Ospedale di Sondrio - ASST Valtellina e dell'Alto Lario, 30 ASST SPEDALI CIVILI DI BRESCIA, 31 PRESIDIO OSPEDALIERO DI CHIARI - ASST Franciacorta, 32 Esine ASST Valcamonica, 33 ASST Papa Giovanni XXIII BERGAMO, 34 OSPEDALE BOLOGNINI - ASST BERGAMO EST, 35 Ospedale Treviglio - Caravaggio ASST Bergamo Ovest, 36 Ospedale di Varese Fondazione Macchi

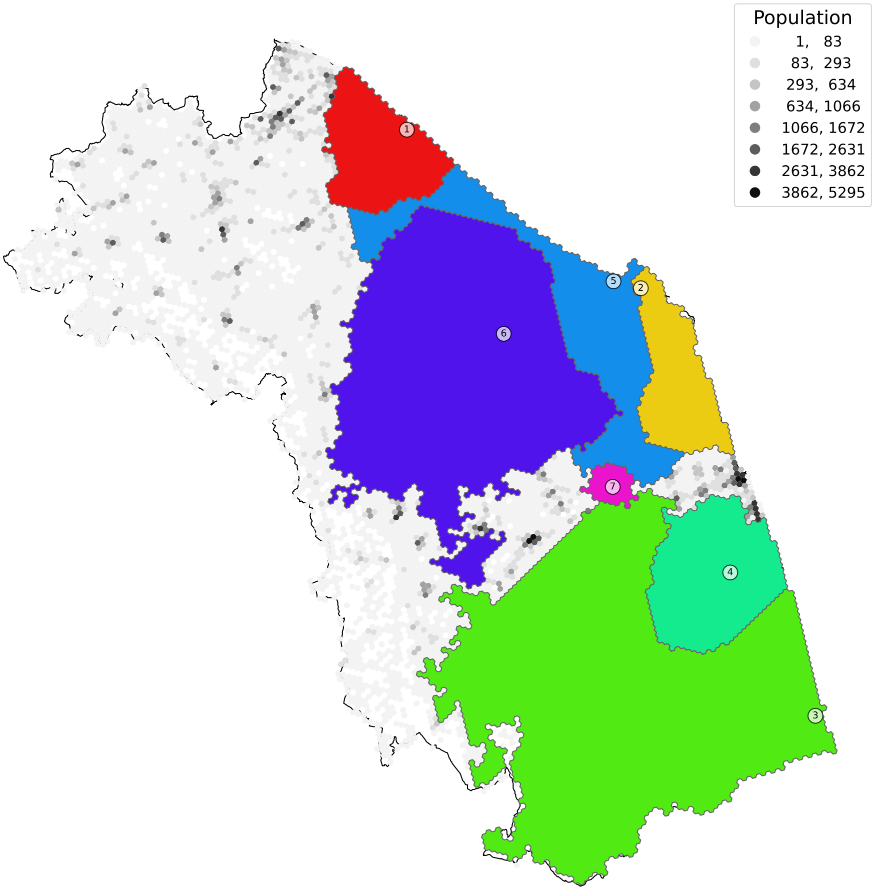

Supplementary Figure 32: Capacity-constrained coverage of stroke unit beds in Marche. Each polygon shows the coverage area of a given hospital . Gray hexagons in the background show populated regions. The legend shows the number of inhabitants according to the shades of gray. 1 Ospedale Santa Croce, 2 INRCA, 3 Madonna del Soccorso, 4 A. Murri, 5 ospedali Riuniti Ancona, 6 Carlo Urbani, 7 Ospedale Provinciale Macerata

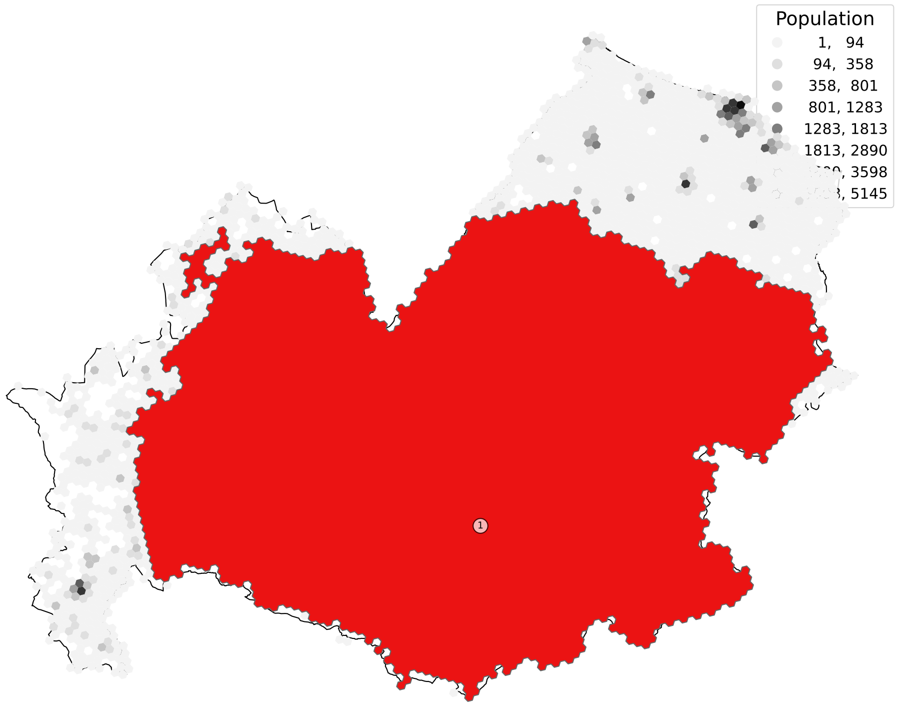

Supplementary Figure 33: Capacity-constrained coverage of stroke unit beds in Molise. Each polygon shows the coverage area of a given hospital . Gray hexagons in the background show populated regions. The legend shows the number of inhabitants according to the shades of gray. 1 PO Cardarelli

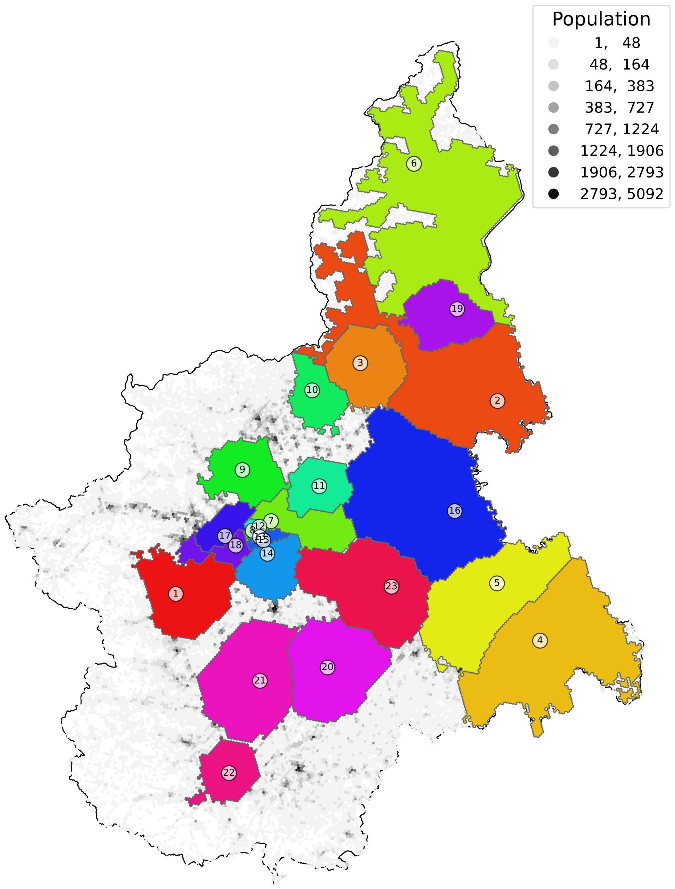

Supplementary Figure 34: Capacity-constrained coverage of stroke unit beds in Piemonte. Each polygon shows the coverage area of a given hospital . Gray hexagons in the background show populated regions. The legend shows the number of inhabitants according to the shades of gray. 1 Ospedale Edoardo Agnelli, 2 Maggiore della Carità Novara, 3 Ospedale degli Infermi, 4 Ospedale Novi Ligure, 5 Santi Antonio e Biagio, 6 San Biagio (Ospedale unico plurisede), 7 San Giovanni Bosco, 8 Ospedale Martini, 9 PO Ciriè, 10 PO Ivrea, 11 PO Chivasso, 12 PO Maria Vittoria, 13 AO Mauriziano, 14 PO Moncalieri, 15 Città della Salute (Molinette), 16 Ospedale Santo Spirito Casale, 17 PO Rivoli, 18 San Luigi, 19 PO Borgomanero, 20 Ospedale Michele e Pietro Ferrero, 21 PO Savigliano, 22 Santa Croce e Carle, 23 PO Asti

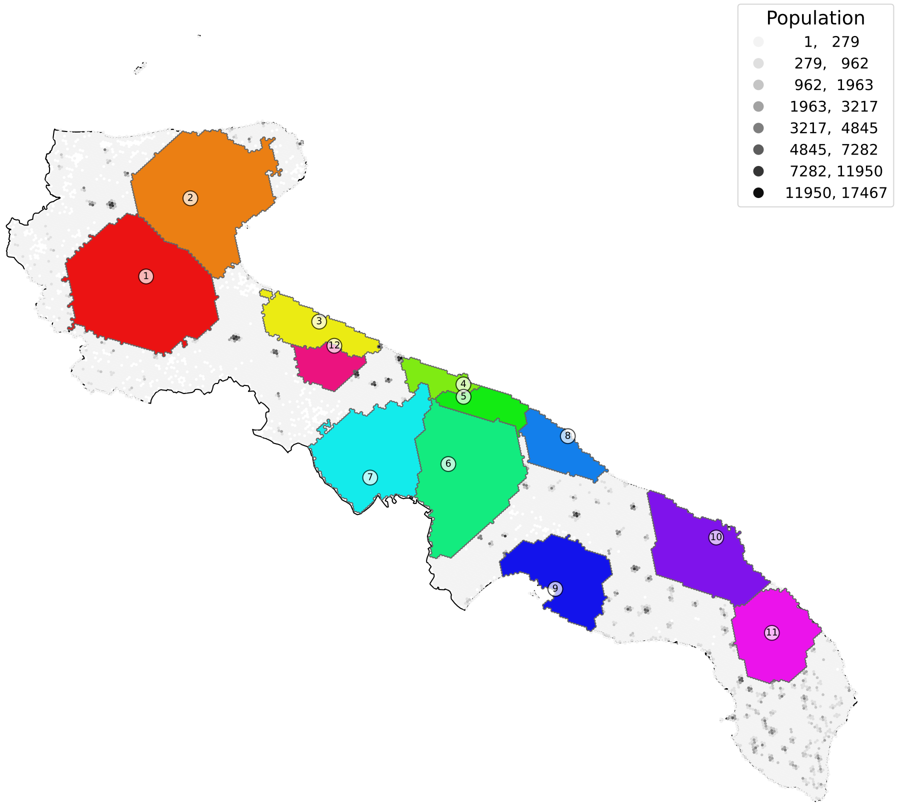

Supplementary Figure 35: Capacity-constrained coverage of stroke unit beds in Puglia. Each polygon shows the coverage area of a given hospital . Gray hexagons in the background show populated regions. The legend shows the number of inhabitants according to the shades of gray. 1 Ospedali Riuniti, 2 Casa Sollievo della Sofferenza, 3 Ospedale Dimiccoli, 4 AOU Policlinico, 5 Ospedale Di Venere, 6 Ospedale Miulli, 7 Ospedale della Murgia F. Perinei, 8 Ospedale San Giacomo, 9 Ospedale SS. Annunziata, 10 Ospedale Perrino, 11 Ospedale Vito Fazzi, 12 Ospedale Andria

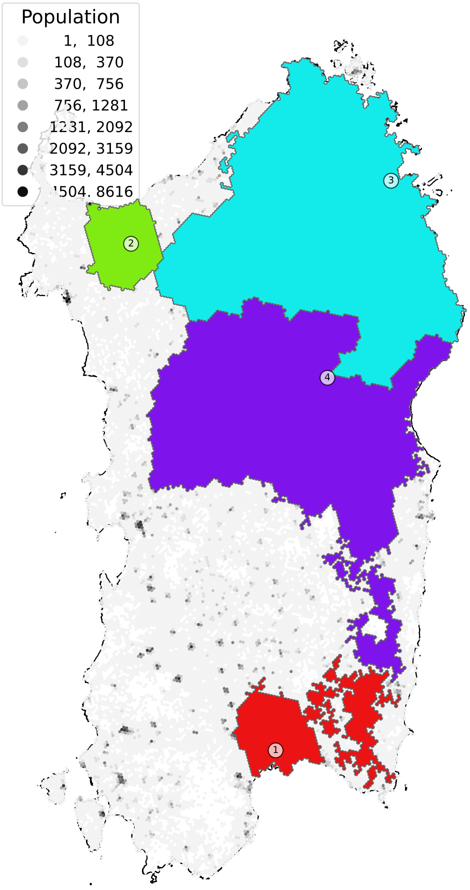

Supplementary Figure 36: Capacity-constrained coverage of stroke unit beds in Sardegna. Each polygon shows the coverage area of a given hospital . Gray hexagons in the background show populated regions. The legend shows the number of inhabitants according to the shades of gray. 1 G. Brotzu, 2 AOU Sassari, 3 Mater Olbia Hospital, 4 San Francesco

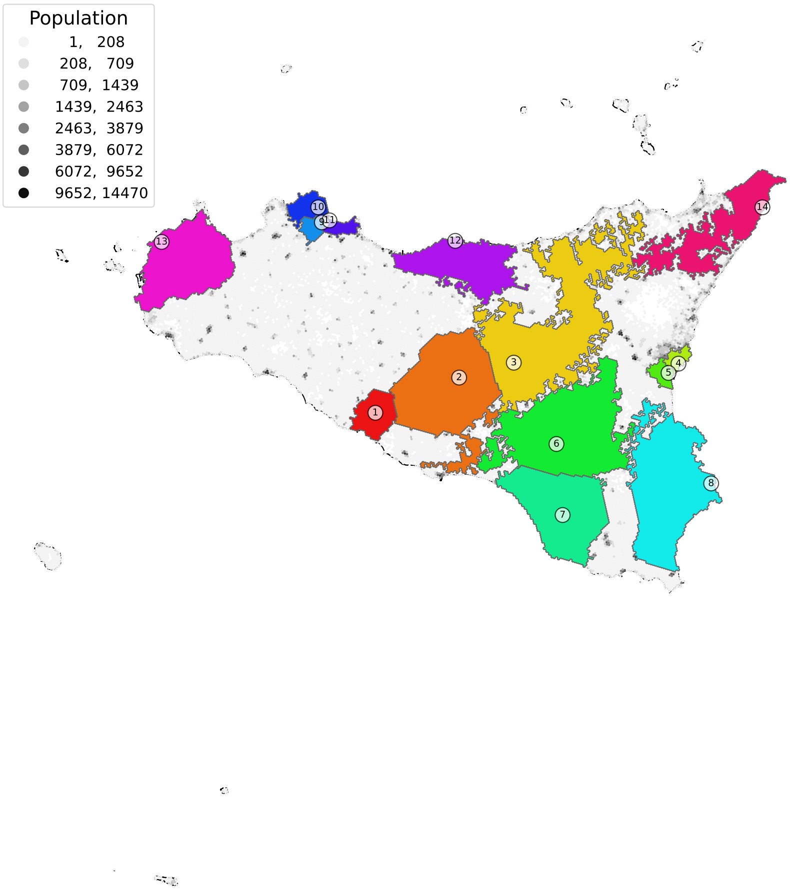

Supplementary Figure 37: Capacity-constrained coverage of stroke unit beds in Sicilia. Each polygon shows the coverage area of a given hospital . Gray hexagons in the background show populated regions. The legend shows the number of inhabitants according to the shades of gray. 1 S. Giovanni di Dio, 2 Sant'Elia, 3 Umberto I, 4 Cannizzaro, 5 Garibaldi Centro, 6 PO Gravina e San Pietro, 7 R. Guzzardi, 8 Umberto I, 9 Civico, 10 Villa Sofia, 11 Buccheri La Ferla, 12 Istituto Giglio, 13 S. Antonio Abate, 14 Policlinico G. martino

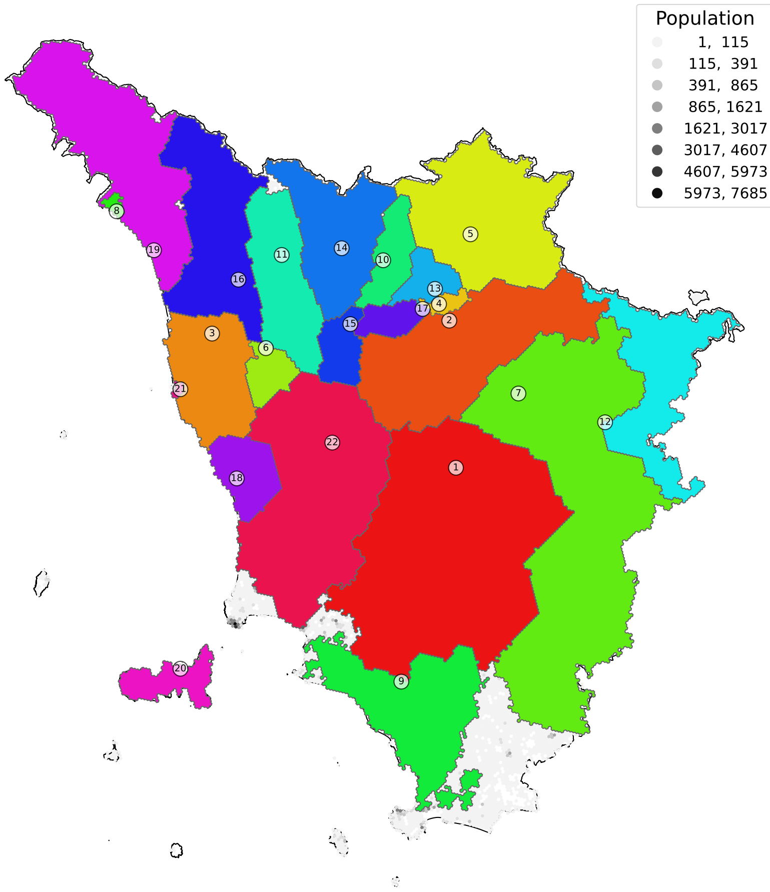

Supplementary Figure 38: Capacity-constrained coverage of stroke unit beds in Toscana. Each polygon shows the coverage area of a given hospital . Gray hexagons in the background show populated regions. The legend shows the number of inhabitants according to the shades of gray. 1 Azienda Ospedaliero Universitaria Senese, 2 Ospedale Santa Maria Annunziata, 3 Azienda Ospedaliero Universitaria Pisana, 4 Ospedale Santa Maria Nuova, 5 Ospedale del Mugello, 6 Ospedale Felice Lotti, 7 Ospedale Santa Maria alla Gruccia - Valdarno, 8 Ospedale Apuane, 9 Ospedale della Misericordia, 10 Ospedale Santo Stefano, 11 Ospedale SS Cosma e Damiano, 12 Ospedale San Donato, 13 Azienda Ospedaliero Universitaria Careggi, 14 Ospedale San Jacopo, 15 Ospedale San Giuseppe, 16 Ospedale San Luca, 17 Ospedale San Giovanni di Dio, 18 Ospedale della Bassa Val di Cecina, 19 Ospedale della Versilia, 20 Ospedale Civile Elbano Portoferraio, 21 Spedali Riuniti Livorno, 22 Ospedale Santa Maria Maddalena

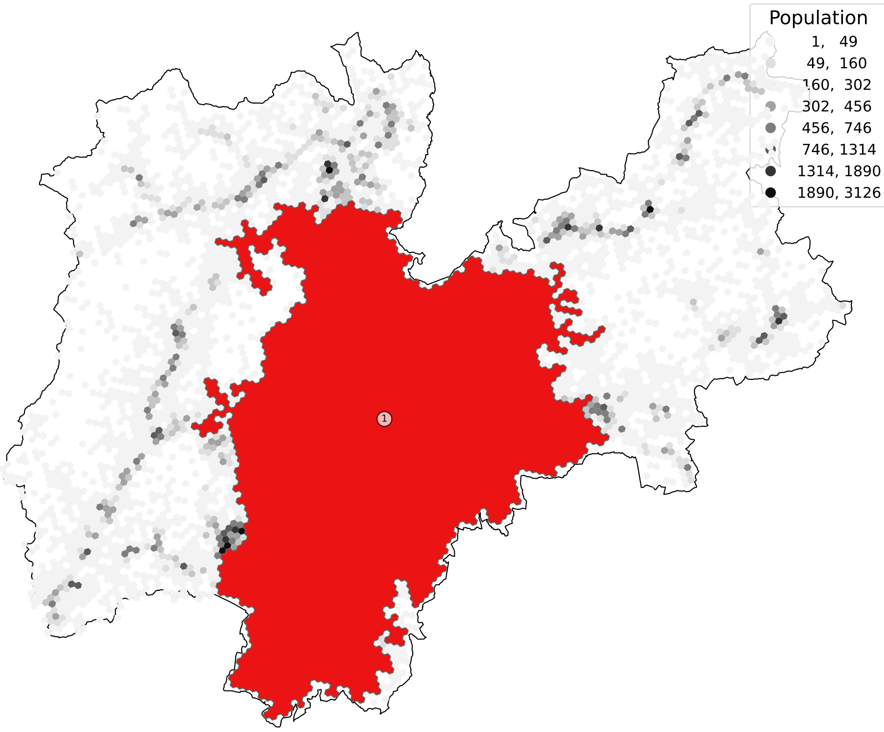

Supplementary Figure 39: Capacity-constrained coverage of stroke unit beds in Trentino. Each polygon shows the coverage area of a given hospital . Gray hexagons in the background show populated regions. The legend shows the number of inhabitants according to the shades of gray. 1 Ospedale Santa Chiara

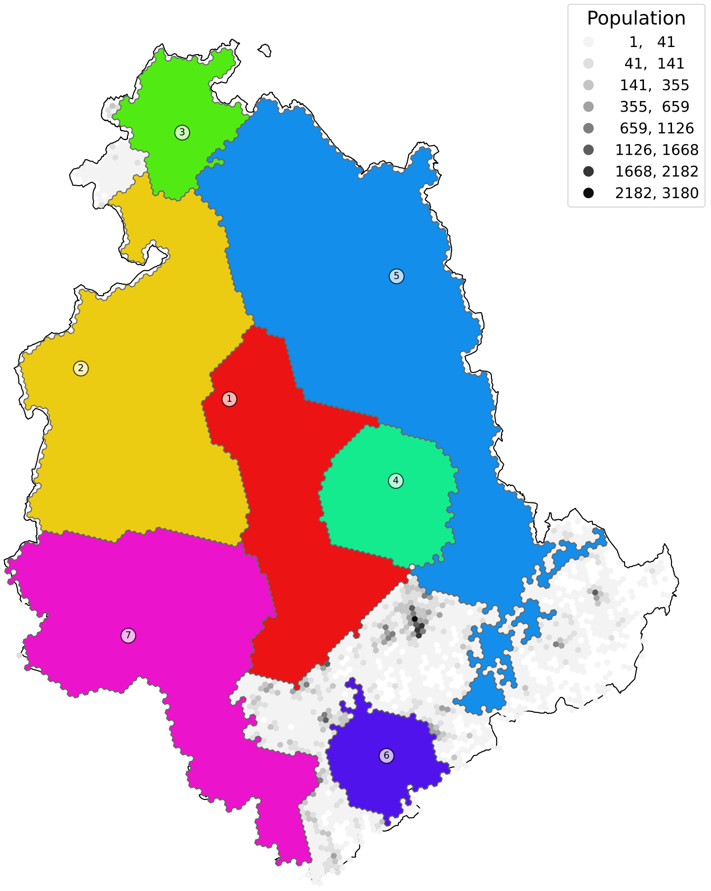

Supplementary Figure 40: Capacity-constrained coverage of stroke unit beds in Umbria. Each polygon shows the coverage area of a given hospital . Gray hexagons in the background show populated regions. The legend shows the number of inhabitants according to the shades of gray. 1 Ospedale Santa Maria della Misericordia, 2 Ospedale Castiglione del Lago, 3 Ospedale di Città di Castello, 4 Ospedale San Giovanni Battista, 5 Presidio Ospedaliero Gubbio e Gualdo Tadino, 6 Azienda Ospedaliera Santa Maria Terni, 7 Ospedale Santa Maria della Stella

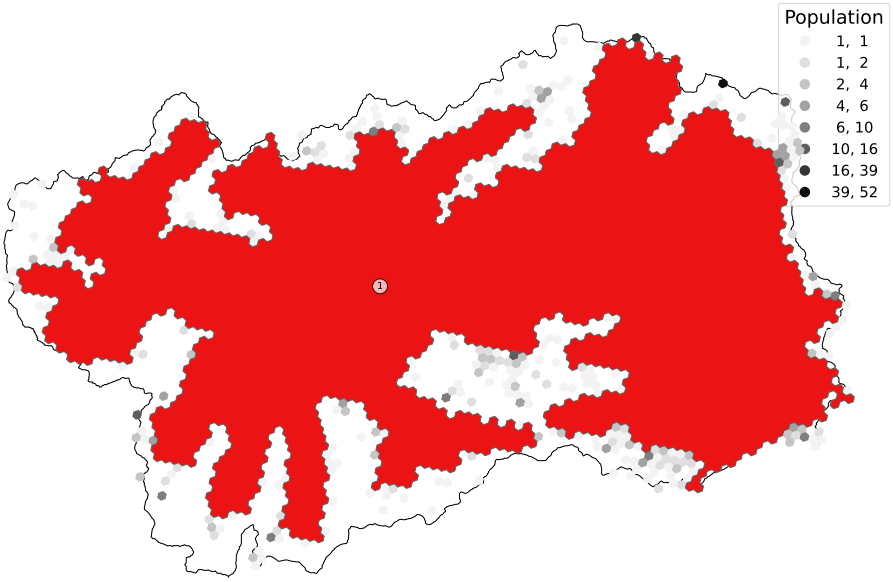

Supplementary Figure 41: Capacity-constrained coverage of stroke unit beds in Valle D'Aosta. Each polygon shows the coverage area of a given hospital . Gray hexagons in the background show populated regions. The legend shows the number of inhabitants according to the shades of gray. 1 Ospedale Umberto Parini

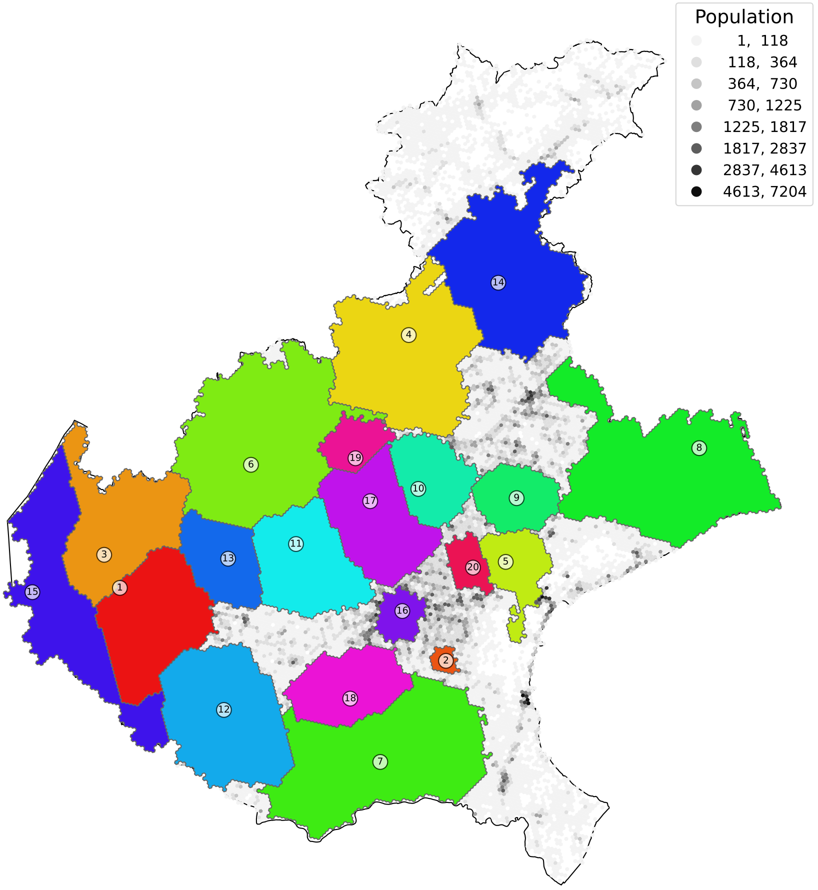

Supplementary Figure 42: Capacity-constrained coverage of stroke unit beds in Veneto. Each polygon shows the coverage area of a given hospital . Gray hexagons in the background show populated regions. The legend shows the number of inhabitants according to the shades of gray. 1 azienda Ospedaliera universitaria Integrata di Verona, 2 Piove di Sacco, 3 IRCSS sacro Cuore Don Calabria, 4 Ospedale di Feltre, 5 Ospedale dell'Angelo, 6 Ospedale di Santorso, 7 Santa Maria Misericordia, 8 San tommaso dei battuti, 9 Ca' Foncello, 10 San Giacomo, 11 San Bortolo, 12 Mater Salutis Legnago, 13 Ospedale Cazzavillan, 14 Ospedale san Martino, 15 Ospedale Pederzoli, 16 Azienda Ospedale Università Padova, 17 Ospedale Civile di Cittadella, 18 Ospedale Riuniti Padova Sud, 19 Ospedale San Bassiano, 20 Ospedale di Mirano ULSS3
