# Supplementary material for: The MAPSTROKE analysis of the access to stroke reperfusion treatment and stroke units in Italy
Source: Eur Stroke J. 2026 Feb 9;11(2):aakaf030. doi: 10.1093/esj/aakaf030 (PMC12884559; doi:10.1093/esj/aakaf030)
Supplement: Supplementary_Tables_Rev1 [file supplementary_tables_rev1.docx]

Supplementary Tables

**The MAPSTROKE analysis of the access to stroke reperfusion treatment and stroke units in Italy.**

**List of Tables:**

[**Supplementary Table 1: Summary of hospital characteristics, according to hospital classification 2**](#_3e6vokp58acy)

[**Supplementary Table 2: Summary of the national and regional 45-minute coverage of the distinct types of hospitals for ischemic stroke reperfusion treatment. 3**](#_eqv44pa8n8rs)

[**Supplementary Table 3: List of hospitals that contributed to the regional coverage for access to reperfusion treatment. 7**](#_ouv1be41l62y)

[**Supplementary Table 4: Sensitivity analysis of distinct thresholds (30, 45, and 60 minutes) for access to reperfusion treatment at the national and regional levels in Italy and their impact on the absolute and relative numbers of covered ischemic strokes. 12**](#_e86irbqjoqz4)

[**Supplementary Table 5: Coverage of stroke unit beds according to the capacity of each hospital per region. 1**](#_v3w6uj53j1df)

#

# Supplementary Table 1: Summary of hospital characteristics, according to hospital classification

| **Characteristic** | **PASC (n = 301)** | **%** | **ASRH (n=22)** | **%** | **PSC (n = 132)** | **%** | **CSC (n = 80)** | **%** |
| --- | --- | --- | --- | --- | --- | --- | --- | --- |
| Acute stroke protocol | 1 | 0.3 | 11 | 50 | 121 | 91.7 | 68 | 85 |
| Emergency Department 24/7 | 294 | 97.7 | 22 | 100 | 132 | 100 | 80 | 100 |
| Laboratory in the ED 24/7 | 294 | 97.7 | 22 | 100 | 132 | 100 | 80 | 100 |
| CT scan available for the ED 24/7 | 285 | 94.7 | 22 | 100 | 132 | 100 | 80 | 100 |
| MR scan available for the ED 24/7 | 0 | 0 | 1 | 4.5 | 14 | 10.6 | 42 | 52.5 |
| CTA or MRA scan available for the ED 24/7 | 100 | 33.2 | 21 | 95.5 | 128 | 97 | 80 | 100 |
| CTP or MRP available for the ED 24/7 | 0 | 0 | 1 | 4.5 | 36 | 27.3 | 59 | 73.8 |
| Neurology support 24/7* | 45 | 15 | 21 | 95.5 | 129 | 97.7 | 80 | 100 |
| Neurology in person 24/7 | 3 | 1 | 4 | 18.2 | 72 | 54.5 | 70 | 87.5 |
| Stroke neurologist support 24/7 | 0 | 0 | 1 | 4.5 | 13 | 9.8 | 16 | 20 |
| Radiology support 24/7 | 276 | 91.7 | 22 | 100 | 131 | 99.2 | 80 | 100 |
| Neurosurgery 24/7 | 8 | 2.7 | 5 | 22.7 | 18 | 13.6 | 75 | 93.8 |
| ICU 24/7 | 147 | 48.8 | 19 | 86.4 | 112 | 84.8 | 79 | 98.8 |
| NeuroICU 24/7 | 1 | 0.3 | 2 | 9.1 | 2 | 1.5 | 21 | 26.3 |
| Stroke Unit | 0 | 0 | 0 | 0 | 132 | 100 | 79 | 98.8 |
| Thrombolysis available | 38 | 12.6 | 22 | 100 | 132 | 100 | 80 | 100 |
| Thrombolysis 24/7 | 37 | 12.3 | 22 | 100 | 132 | 100 | 80 | 100 |
| Thrombolysis via telemedicine | 24 | 8 | 9 | 40.9 | 35 | 26.5 | 12 | 15 |
| Thrombectomy available | 0 | 0 | 0 | 0 | 5 | 3.8 | 80 | 100 |

* in person, on call or telemedicine

Potential Acute Stroke Center (PASC), hospitals with an emergency department (ED), neuroimaging, and laboratory services, but without established acute stroke care protocols.

Acute Stroke-Ready Hospital (ASRH): performs IVT and transfers the patient to other facilities for additional reperfusion treatment and/or hospitalization.

Primary Stroke Center (PSC): performs IVT and admits the patient for in-hospital post-acute care.

Comprehensive Stroke Center (CSC): performs both IVT and EVT, and admits patients.

#

#

# Supplementary Table 2: Summary of the national and regional 45-minute coverage of the distinct types of hospitals for ischemic stroke reperfusion treatment.

Hospital Types:

[1] Potential Acute Stroke Center (PASC), hospitals with an emergency department (ED), neuroimaging, and laboratory services, but without established acute stroke care protocols.

[2] Acute Stroke-Ready Hospital (ASRH): performs IVT and transfers the patient to other facilities for additional reperfusion treatment and/or hospitalization.

[3] Primary Stroke Center (PSC): performs IVT and admits the patient for in-hospital post-acute care.

[4] Comprehensive Stroke Center (CSC): performs both IVT and EVT, and admits patients.

*Note 1: The merged coverage area of hospital types (i.e., [3,4]) may result in overlapped icoshrones.*

*Note 2: [1,2,3,4] coverages are the sum of the coverages of all hospitals within the given type(s).*

| **Territory** | **Types** | **Population Coverage, N** | **Ischemic**  **Stroke Coverage (calculated, N)** | **Stroke coverage, %** |
| --- | --- | --- | --- | --- |
| National | [1] | 52157004 | 59726 | 87.64% |
|  | [2] | 13814519 | 14782 | 22.29% |
|  | [3] | 44814964 | 52164 | 76.79% |
|  | [4] | 44806991 | 51552 | 75.71% |
|  | [3, 4] | 53225299 | 61632 | 90.72% |
|  | [2, 3, 4] | 53843876 | 62267 | 91.69% |
|  | [1, 2, 3, 4] | 57917414 | 66800 | 98.35% |
| Abruzzo | [1] | 753935 | 923 | 59.45% |
|  | [2] | 0 | 0 | 0.00% |
|  | [3] | 991822 | 1215 | 78.21% |
|  | [4] | 828246 | 1014 | 65.31% |
|  | [3, 4] | 1195732 | 1464 | 94.29% |
|  | [2, 3, 4] | 1195732 | 1464 | 94.29% |
|  | [1, 2, 3, 4] | 1244954 | 1525 | 98.17% |
| Alto-Adige | [1] | 467425 | 626 | 91.03% |
|  | [2] | 0 | 0 | 0.00% |
|  | [3] | 0 | 0 | 0.00% |
|  | [4] | 322572 | 432 | 62.82% |
|  | [3, 4] | 322572 | 432 | 62.82% |
|  | [2, 3, 4] | 322572 | 432 | 62.82% |
|  | [1, 2, 3, 4] | 499178 | 669 | 97.22% |
| Basilicata | [1] | 102717 | 101 | 17.86% |
|  | [2] | 0 | 0 | 0.00% |
|  | [3] | 195021 | 191 | 33.90% |
|  | [4] | 0 | 0 | 0.00% |
|  | [3, 4] | 195021 | 191 | 33.90% |
|  | [2, 3, 4] | 195021 | 191 | 33.90% |
|  | [1, 2, 3, 4] | 297738 | 292 | 51.76% |
| Calabria | [1] | 1230917 | 1190 | 67.15% |
|  | [2] | 0 | 0 | 0.00% |
|  | [3] | 453469 | 438 | 24.74% |
|  | [4] | 923686 | 893 | 50.39% |
|  | [3, 4] | 1231010 | 1190 | 67.15% |
|  | [2, 3, 4] | 1231010 | 1190 | 67.15% |
|  | [1, 2, 3, 4] | 1732932 | 1675 | 94.53% |
| Campania | [1] | 5417781 | 4620 | 98.29% |
|  | [2] | 3129562 | 2669 | 56.78% |
|  | [3] | 4894510 | 4174 | 88.80% |
|  | [4] | 4571969 | 3899 | 82.95% |
|  | [3, 4] | 5033059 | 4292 | 91.31% |
|  | [2, 3, 4] | 5227747 | 4458 | 94.85% |
|  | [1, 2, 3, 4] | 5456547 | 4653 | 99.00% |
| Emilia-Romagna | [1] | 4322352 | 5958 | 97.36% |
|  | [2] | 0 | 0 | 0.00% |
|  | [3] | 3272253 | 4511 | 73.71% |
|  | [4] | 3650679 | 5032 | 82.23% |
|  | [3, 4] | 4114108 | 5671 | 92.67% |
|  | [2, 3, 4] | 4114108 | 5671 | 92.67% |
|  | [1, 2, 3, 4] | 4374074 | 6030 | 98.52% |
| Friuli-Venezia Giulia | [1] | 1022795 | 1443 | 86.30% |
|  | [2] | 0 | 0 | 0.00% |
|  | [3] | 697742 | 984 | 58.87% |
|  | [4] | 826010 | 1165 | 69.69% |
|  | [3, 4] | 1124262 | 1586 | 94.86% |
|  | [2, 3, 4] | 1124262 | 1586 | 94.86% |
|  | [1, 2, 3, 4] | 1174856 | 1657 | 99.13% |
| Lazio | [1] | 5387949 | 5688 | 96.68% |
|  | [2] | 4740505 | 5004 | 85.07% |
|  | [3] | 4818101 | 5086 | 86.46% |
|  | [4] | 4566516 | 4821 | 81.94% |
|  | [3, 4] | 5167430 | 5455 | 92.73% |
|  | [2, 3, 4] | 5380858 | 5680 | 96.56% |
|  | [1, 2, 3, 4] | 5558978 | 5869 | 99.75% |
| Liguria | [1] | 1381196 | 2536 | 92.34% |
|  | [2] | 0 | 0 | 0.00% |
|  | [3] | 1469247 | 2697 | 98.23% |
|  | [4] | 1085122 | 1992 | 72.55% |
|  | [3, 4] | 1471997 | 2702 | 98.41% |
|  | [2, 3, 4] | 1471997 | 2702 | 98.41% |
|  | [1, 2, 3, 4] | 1482950 | 2723 | 99.15% |
| Lombardia | [1] | 9565193 | 10630 | 98.45% |
|  | [2] | 890498 | 990 | 9.17% |
|  | [3] | 8725743 | 9698 | 89.81% |
|  | [4] | 9055012 | 10063 | 93.20% |
|  | [3, 4] | 9397364 | 10444 | 96.72% |
|  | [2, 3, 4] | 9508173 | 10567 | 97.86% |
|  | [1, 2, 3, 4] | 9684593 | 10763 | 99.68% |
| Marche | [1] | 1472275 | 1636 | 97.03% |
|  | [2] | 0 | 0 | 0.00% |
|  | [3] | 1398761 | 1554 | 92.18% |
|  | [4] | 534128 | 593 | 35.20% |
|  | [3, 4] | 1398846 | 1554 | 92.19% |
|  | [2, 3, 4] | 1398846 | 1554 | 92.19% |
|  | [1, 2, 3, 4] | 1498717 | 1665 | 98.77% |
| Molise | [1] | 189095 | 211 | 61.79% |
|  | [2] | 0 | 0 | 0.00% |
|  | [3] | 121615 | 136 | 39.74% |
|  | [4] | 0 | 0 | 0.00% |
|  | [3, 4] | 121615 | 136 | 39.74% |
|  | [2, 3, 4] | 121615 | 136 | 39.74% |
|  | [1, 2, 3, 4] | 282879 | 316 | 92.44% |
| Piemonte | [1] | 3731609 | 4751 | 87.04% |
|  | [2] | 983602 | 1252 | 22.94% |
|  | [3] | 4028414 | 5129 | 93.96% |
|  | [4] | 3434101 | 4373 | 80.10% |
|  | [3, 4] | 4168229 | 5307 | 97.22% |
|  | [2, 3, 4] | 4186443 | 5330 | 97.65% |
|  | [1, 2, 3, 4] | 4260276 | 5424 | 99.37% |
| Puglia | [1] | 3939291 | 3619 | 96.37% |
|  | [2] | 0 | 0 | 0.00% |
|  | [3] | 2554528 | 2347 | 62.49% |
|  | [4] | 3473711 | 3191 | 84.98% |
|  | [3, 4] | 3813985 | 3504 | 93.30% |
|  | [2, 3, 4] | 3813985 | 3504 | 93.30% |
|  | [1, 2, 3, 4] | 4033616 | 3705 | 98.68% |
| Sardegna | [1] | 1386812 | 1551 | 85.62% |
|  | [2] | 0 | 0 | 0.00% |
|  | [3] | 0 | 0 | 0.00% |
|  | [4] | 1029189 | 1151 | 63.54% |
|  | [3, 4] | 1029189 | 1151 | 63.54% |
|  | [2, 3, 4] | 1029189 | 1151 | 63.54% |
|  | [1, 2, 3, 4] | 1582591 | 1769 | 97.71% |
| Sicilia | [1] | 4341040 | 4741 | 88.47% |
|  | [2] | 1053420 | 1151 | 21.47% |
|  | [3] | 2617819 | 2859 | 53.35% |
|  | [4] | 2880934 | 3147 | 58.71% |
|  | [3, 4] | 4026772 | 4398 | 82.07% |
|  | [2, 3, 4] | 4027301 | 4399 | 82.08% |
|  | [1, 2, 3, 4] | 4798422 | 5241 | 97.79% |
| Toscana | [1] | 1357689 | 1813 | 37.51% |
|  | [2] | 119985 | 160 | 3.32% |
|  | [3] | 3297806 | 4405 | 91.12% |
|  | [4] | 2422359 | 3235 | 66.93% |
|  | [3, 4] | 3385232 | 4522 | 93.54% |
|  | [2, 3, 4] | 3395332 | 4535 | 93.82% |
|  | [1, 2, 3, 4] | 3599000 | 4807 | 99.44% |
| Trentino | [1] | 521346 | 564 | 95.84% |
|  | [2] | 336664 | 364 | 61.89% |
|  | [3] | 0 | 0 | 0.00% |
|  | [4] | 374751 | 405 | 68.89% |
|  | [3, 4] | 374751 | 405 | 68.89% |
|  | [2, 3, 4] | 403642 | 436 | 74.20% |
|  | [1, 2, 3, 4] | 528357 | 571 | 97.13% |
| Umbria | [1] | 827126 | 1216 | 92.66% |
|  | [2] | 0 | 0 | 0.00% |
|  | [3] | 712634 | 1047 | 79.83% |
|  | [4] | 641078 | 942 | 71.82% |
|  | [3, 4] | 876743 | 1289 | 98.22% |
|  | [2, 3, 4] | 876743 | 1289 | 98.22% |
|  | [1, 2, 3, 4] | 892328 | 1312 | 99.96% |
| Valle D’Aosta | [1] | 0 | 0 | 0.00% |
|  | [2] | 0 | 0 | 0.00% |
|  | [3] | 0 | 0 | 0.00% |
|  | [4] | 118728 | 130 | 90.19% |
|  | [3, 4] | 118728 | 130 | 90.19% |
|  | [2, 3, 4] | 118728 | 130 | 90.19% |
|  | [1, 2, 3, 4] | 118728 | 130 | 90.19% |
| Veneto | [1] | 4738461 | 5909 | 96.89% |
|  | [2] | 2560283 | 3192 | 52.35% |
|  | [3] | 4565479 | 5693 | 93.36% |
|  | [4] | 4068200 | 5073 | 83.19% |
|  | [3, 4] | 4658654 | 5809 | 95.26% |
|  | [2, 3, 4] | 4700572 | 5861 | 96.12% |
|  | [1, 2, 3, 4] | 4815700 | 6005 | 98.47% |

# Supplementary Table 3: List of hospitals that contributed to the regional coverage for access to reperfusion treatment.

Hospital names are as provided by the respondents in the survey. In regions with current coverage below 90%, the Maximum Coverage Location Problem was run until the 90% threshold was reached. Hospitals Type 1 (PASCs) added after the optimization are highlighted in the table.

Hospital Types:

[1] Potential Acute Stroke Center (PASC), hospitals with an emergency department (ED), neuroimaging, and laboratory services, but without established acute stroke care protocols.

[2] Acute Stroke-Ready Hospital (ASRH): performs IVT and transfers the patient to other facilities for additional reperfusion treatment and/or hospitalization.

[3] Primary Stroke Center (PSC): performs IVT and admits the patient for in-hospital post-acute care.

[4] Comprehensive Stroke Center (CSC): performs both IVT and EVT, and admits patients.

| **Region (coverage %)** | **Hospital Name** | **Hospital Type** |
| --- | --- | --- |
| Abruzzo (94.29%) | SS Filippo e Nicola | 4 |
|  | Ospedale Civile Spirito Santo | 4 |
|  | San Salvatore | 4 |
|  | Ospedale F. Renzetti | 3 |
|  | Ospedale Giuseppe Mazzini | 3 |
|  | Policlinico SS Annunziata | 3 |
|  | San Pio da Pietrelcina | 3 |
| Alto-Adige (92.95%) | Ospedale centrale di Bolzano | 4 |
|  | Silandro H. | 1 |
|  | Bressanone H. | 1 |
|  | Brunico H. | 1 |
| Basilicata (51.76%) | AOR San Carlo | 3 |
|  | PO Madonna delle Grazie | 1 |
| Calabria (91.05%) | AO SS. Annunziata | 4 |
|  | AOU Bianchi-Melacrino-Morelli | 4 |
|  | AO Pugliese Ciaccio | 4 |
|  | Ospedale civile San Giovanni di Dio | 3 |
|  | PO Jazzolino | 3 |
|  | Ospedale Civile San Giovanni in Fiore | 1 |
|  | Tirrenia hospital | 1 |
|  | P.O.Lamezia Terme | 1 |
|  | Ospedale Civile Locri | 1 |
|  | Ospedale Civile | 1 |
|  | Ospedale di Corigliano | 1 |
| Campania (94.85%) | AORN Cardarelli | 4 |
|  | A.O.U. Federico II | 4 |
|  | P.O. San Giovanni Bosco | 4 |
|  | PO Ospedale del Mare | 4 |
|  | PO Umberto I | 4 |
|  | AOU San Giovanni di Dio e Ruggi D'Aragona | 4 |
|  | AO San Sebastiano | 4 |
|  | PO Luigi Curto | 3 |
|  | AO S.G. Moscati | 3 |
|  | PO San Leonardo | 3 |
|  | PO Sant'Ottone Frangipane | 3 |
|  | PO San Giuseppe Moscati | 3 |
|  | PO S. Maria delle Grazie | 2 |
|  | PO San Luca | 2 |
|  | AO San Pio | 2 |
| Emilia-Romagna (92.67%) | Ospedale Maggiore | 4 |
|  | Maggiore | 4 |
|  | Ospedale sant'Anna | 4 |
|  | Ospedale Civile di Baggiovara, AOU Modena | 4 |
|  | Ospedale Bufalini | 4 |
|  | Ospedale di Fidenza | 3 |
|  | Santa Maria della Scaletta | 3 |
|  | Santa Maria delle Croci | 3 |
|  | Arcispedale Santa Maria Nuova | 3 |
|  | Ospedale Infermi | 3 |
|  | Morgagni-Pierantoni | 3 |
|  | Ospedale Guglielmo da Saliceto | 3 |
| Friuli-Venezia Giulia (94.86%) | Azienda sanitaria Universitaria giuliano Isontina | 4 |
|  | Santa Maria della Misericordia | 4 |
|  | PO Gorizia/Monfalcone | 3 |
|  | Santa Maria degli Angeli | 3 |
| Lazio (96.56%) | Azienda Ospedaliera San Camillo | 4 |
|  | Fondazione Policlinico Universitario Agostino Gemelli | 4 |
|  | Policlinico Tor Vergata | 4 |
|  | Policlinico Umberto I | 4 |
|  | Ospedale Belcolle | 4 |
|  | Ospedale Santa Maria Goretti | 4 |
|  | Policlinico Campus Bio-Medico | 3 |
|  | AOU Sant'Andrea | 3 |
|  | Ospedale dei Castelli | 3 |
|  | Ospedale Fabrizio Spaziani | 3 |
|  | Azienda Ospedaliera San Giovanni Addolorata | 3 |
|  | Ospedale Sant’Eugenio | 3 |
|  | Ospedale San Camillo de Lellis | 3 |
|  | Ospedale San Filippo Neri | 3 |
|  | Ospedale Sandro Pertini | 2 |
|  | Policlinico Casilino | 2 |
|  | Ospedale G. B. Grassi | 2 |
|  | Ospedale Tivoli | 2 |
|  | Ospedale Palestrina | 2 |
|  | Ospedale Colleferro | 2 |
|  | Ospedale Anzio / Nettuno | 2 |
|  | Ospedale Fiorini | 2 |
|  | Ospedale Dono Svizzero | 2 |
| Liguria (98.41%) | University of Genova and San Martino Hospital | 4 |
|  | Ospedale Santa Corona | 4 |
|  | Ospedale Imperia | 3 |
|  | E.O. Ospedali Galliera | 3 |
|  | Presidio Ospedaliero Sant'Andrea La Spezia | 3 |
|  | P.O.M Villa Scassi ASL3 Genova | 3 |
|  | San Paolo | 3 |
|  | Ospedale di Lavagna | 3 |
| Lombardia (97.86%) | ASST Grande Ospedale Metropolitano Niguarda | 4 |
|  | OSPEDALE CIVILE LEGNANO - ASST OVEST MILANESE | 4 |
|  | FONDAZIONE IRCCS CA GRANDA OSPEDALE MAGGIORE POLICLINICO | 4 |
|  | OSPEDALE ALESSANDRO MANZONI - ASST Lecco | 4 |
|  | OSPEDALE SAN GERARDO DEI TINTORI- ASST Monza | 4 |
|  | OSPEDALE SAN CARLO BORROMEO - ASST Santi Paolo e Carlo | 4 |
|  | ASST SPEDALI CIVILI DI BRESCIA | 4 |
|  | Fondazione Poliambulanza Brescia | 4 |
|  | Ospedale di Varese Fondazione Macchi | 4 |
|  | ASST Papa Giovanni XXIII BERGAMO | 4 |
|  | IRCCS OSPEDALE SAN RAFFAELE | 4 |
|  | Ospedale Sant Anna di Como- ASST Lariana | 4 |
|  | IRCCS POLICLINICO SAN MATTEO-MONDINO | 4 |
|  | Istituto Clinico Humanitas | 4 |
|  | OSPEDALE DI CREMONA - ASST Cremona | 4 |
|  | OSPEDALE CARLO POMA - ASST Mantova | 4 |
|  | ISTITUTO CLINICO CITTA STUDI | 3 |
|  | OSPEDALE GUIDO SALVINI GARBAGNATE - ASST Rhodense | 3 |
|  | IRCCS Policlinico San Donato | 3 |
|  | Ospedale Sacco | 3 |
|  | OSPEDALE DI DESIO | 3 |
|  | OSPEDALE SAN LEOPOLDO MANDIC Merate | 3 |
|  | Esine ASST Valcamonica | 3 |
|  | OSPEDALE MORIGGIA - PELASCINI - ASST Sette Laghi | 3 |
|  | OSPEDALE VALDUCE - ASST LARIANA | 3 |
|  | Ospedale Civile di Voghera - ASST Pavia | 3 |
|  | OSPEDALE DI VIZZOLO PREDABISSI - ASST Melegnano - Martesana | 3 |
|  | ASST Brianza - Vimercate | 3 |
|  | Ospedale Maggiore di Lodi - ASST Lodi | 3 |
|  | Ospedale di Circolo di Saronno - ASST della Valle Olona | 3 |
|  | OSPEDALE BOLOGNINI - ASST BERGAMO EST | 3 |
|  | OSPEDALE MAGGIORE DI CREMA- ASST Crema | 3 |
|  | Gallarate- ASST Valle Olona | 3 |
|  | Ospedale di Sondrio - ASST Valtellina e dell’Alto Lario | 3 |
|  | Ospedale Treviglio - Caravaggio ASST Bergamo Ovest | 3 |
|  | PRESIDIO OSPEDALIERO DI CHIARI - ASST Franciacorta | 3 |
|  | Ospedale Civile di Vigevano | 2 |
|  | Presidio Ospedaliero di Sondalo | 2 |
| Marche (92.19%) | ospedali Riuniti Ancona | 4 |
|  | INRCA | 3 |
|  | Ospedale Santa Croce | 3 |
|  | Madonna del Soccorso | 3 |
|  | A. Murri | 3 |
|  | Carlo Urbani | 3 |
|  | Ospedale Provinciale Macerata | 3 |
| Molise (92.44%) | PO Cardarelli | 3 |
|  | San Timoteo | 1 |
|  | Ferdinando Veneziale | 1 |
| Piemonte (97.65%) | Maggiore della Carità Novara | 4 |
|  | San Giovanni Bosco | 4 |
|  | Santi Antonio e Biagio | 4 |
|  | Santa Croce e Carle | 4 |
|  | Città della Salute (Molinette) | 4 |
|  | San Biagio (Ospedale unico plurisede) | 3 |
|  | Ospedale Novi Ligure | 3 |
|  | Ospedale Edoardo Agnelli | 3 |
|  | Ospedale degli Infermi | 3 |
|  | Ospedale Santo Spirito Casale | 3 |
|  | Ospedale Martini | 3 |
|  | PO Chivasso | 3 |
|  | PO Ciriè | 3 |
|  | PO Maria Vittoria | 3 |
|  | AO Mauriziano | 3 |
|  | PO Moncalieri | 3 |
|  | PO Ivrea | 3 |
|  | PO Savigliano | 3 |
|  | PO Rivoli | 3 |
|  | PO Borgomanero | 3 |
|  | San Luigi | 3 |
|  | PO Asti | 3 |
|  | Ospedale Michele e Pietro Ferrero | 3 |
|  | Regina Montis Regalis - ASL CN1 | 2 |
|  | PO Vercelli | 2 |
| Puglia (93.30%) | Ospedali Riuniti | 4 |
|  | Casa Sollievo della Sofferenza | 4 |
|  | AOU Policlinico | 4 |
|  | Ospedale Vito Fazzi | 4 |
|  | Ospedale SS. Annunziata | 4 |
|  | Ospedale Andria | 4 |
|  | Ospedale Dimiccoli | 3 |
|  | Ospedale Di Venere | 3 |
|  | Ospedale San Giacomo | 3 |
|  | Ospedale della Murgia F. Perinei | 3 |
|  | Ospedale Miulli | 3 |
|  | Ospedale Perrino | 3 |
| Sardegna (90.38%) | G. Brotzu | 4 |
|  | AOU Sassari | 4 |
|  | Mater Olbia Hospital | 4 |
|  | San Francesco | 4 |
|  | P.O. Paolo Dettori | 1 |
|  | P.O. GP Delogu | 1 |
|  | P.O NSB Bonaria | 1 |
|  | P.O. Sirai | 1 |
|  | P.O. Nostra Signora della Mercede | 1 |
| Sicilia (90.17%) | Cannizzaro | 4 |
|  | Sant'Elia | 4 |
|  | Villa Sofia | 4 |
|  | Policlinico G. martino | 4 |
|  | Garibaldi Centro | 4 |
|  | Civico | 4 |
|  | S. Antonio Abate | 3 |
|  | S. Giovanni di Dio | 3 |
|  | PO Gravina e San Pietro | 3 |
|  | Umberto I | 3 |
|  | Istituto Giglio | 3 |
|  | Umberto I | 3 |
|  | R. Guzzardi | 3 |
|  | Buccheri La Ferla | 3 |
|  | Policlinico Giaccone | 2 |
|  | P.O. Vittorio Emanuele | 1 |
|  | Ospedale Maggiore | 1 |
|  | P.O. Vittorio Emanuele II | 1 |
| Toscana (93.82%) | Azienda Ospedaliero Universitaria Senese | 4 |
|  | Azienda Ospedaliero Universitaria Pisana | 4 |
|  | Azienda Ospedaliero Universitaria Careggi | 4 |
|  | Ospedale Santa Maria Nuova | 3 |
|  | Ospedale del Mugello | 3 |
|  | Ospedale Felice Lotti | 3 |
|  | Ospedale Santa Maria Annunziata | 3 |
|  | Ospedale Santa Maria alla Gruccia - Valdarno | 3 |
|  | Ospedale Apuane | 3 |
|  | Ospedale Santo Stefano | 3 |
|  | Ospedale della Misericordia | 3 |
|  | Ospedale SS Cosma e Damiano | 3 |
|  | Ospedale San Donato | 3 |
|  | Ospedale San Jacopo | 3 |
|  | Ospedale San Giuseppe | 3 |
|  | Ospedale San Luca | 3 |
|  | Ospedale San Giovanni di Dio | 3 |
|  | Ospedale della Bassa Val di Cecina | 3 |
|  | Ospedale della Versilia | 3 |
|  | Spedali Riuniti Livorno | 3 |
|  | Ospedale Civile Elbano Portoferraio | 3 |
|  | Ospedale Santa Maria Maddalena | 3 |
|  | Ospedale Villamarina | 2 |
| Trentino (93.84%) | Ospedale Santa Chiara | 4 |
|  | Ospedale di Rovereto | 2 |
|  | P.O. di Cles | 1 |
|  | P.O. di Cavalese | 1 |
|  | P.O. di Tione di Trento | 1 |
| Umbria (98.22%) | Ospedale Santa Maria della Misericordia | 4 |
|  | Azienda Ospedaliera Santa Maria Terni | 4 |
|  | Ospedale Castiglione del Lago | 3 |
|  | Ospedale di Città di Castello | 3 |
|  | Ospedale San Giovanni Battista | 3 |
|  | Presidio Ospedaliero Gubbio e Gualdo Tadino | 3 |
|  | Ospedale Santa Maria della Stella | 3 |
| Valle D’Aosta (90.19%) | Ospedale Umberto Parini | 4 |
| Veneto (96.12%) | azienda Ospedaliera universitaria Integrata di Verona | 4 |
|  | Ospedale dell'Angelo | 4 |
|  | Santa Maria Misericordia | 4 |
|  | Azienda Ospedale Università Padova | 4 |
|  | San Bortolo | 4 |
|  | Ca' Foncello | 4 |
|  | Piove di Sacco | 3 |
|  | Ospedale San Bassiano | 3 |
|  | Ospedale di Santorso | 3 |
|  | IRCSS sacro Cuore Don Calabria | 3 |
|  | Ospedale di Feltre | 3 |
|  | Mater Salutis Legnago | 3 |
|  | San Giacomo | 3 |
|  | Ospedale Cazzavillan | 3 |
|  | San tommaso dei battuti | 3 |
|  | Ospedale di Mirano ULSS3 | 3 |
|  | Ospedale san Martino | 3 |
|  | Ospedale Pederzoli | 3 |
|  | Ospedale Civile di Cittadella | 3 |
|  | Ospedale Riuniti Padova Sud | 3 |
|  | Ospedale di Conegliano e Vittorio Veneto | 2 |
|  | Ospedale Civile SS Giovanni e Paolo | 2 |
|  | Ospedale Sant'Antonio | 2 |

# Supplementary Table 4: Sensitivity analysis of distinct thresholds (30, 45, and 60 minutes) for access to reperfusion treatment at the national and regional levels in Italy and their impact on the absolute and relative numbers of covered ischemic strokes.

Hospital Types:

[1] Potential Acute Stroke Center (PASC), hospitals with an emergency department (ED), neuroimaging, and laboratory services, but without established acute stroke care protocols.

[2] Acute Stroke-Ready Hospital (ASRH): performs IVT and transfers the patient to other facilities for additional reperfusion treatment and/or hospitalization.

[3] Primary Stroke Center (PSC): performs IVT and admits the patient for in-hospital post-acute care.

[4] Comprehensive Stroke Center (CSC): performs both IVT and EVT, and admits patients.

*Note 1: The merged coverage area of hospital types (i.e., [3,4]) may result in overlapped icoshrones.*

*Note 2: [1,2,3,4] coverages are the sum of the coverages of all hospitals within the given type(s).*

| **Territory** | **Types** | **Ischemic stroke coverage in 30 minutes (absolute)** | **Ischemic stroke coverage in 30 minutes (%)** | **Ischemic stroke coverage in 45 minutes (absolute)** | **Ischemic stroke coverage in 45 minutes (%)** | **Ischemic stroke coverage in 60 minutes (absolute)** | **Ischemic stroke coverage in 60 minutes (%)** |
| --- | --- | --- | --- | --- | --- | --- | --- |
| National | [1] | 49738 | 73.27% | 59726 | 87.64% | 64403 | 94.87% |
|  | [2] | 9207 | 13.56% | 14782 | 22.29% | 22732 | 33.49% |
|  | [3] | 40856 | 60.19% | 52164 | 76.79% | 57986 | 85.42% |
|  | [4] | 37300 | 54.95% | 51552 | 75.71% | 59092 | 87.05% |
|  | [3, 4] | 52660 | 77.57% | 61632 | 90.72% | 64889 | 95.59% |
|  | [2, 3, 4] | 54040 | 79.61% | 62267 | 91.69% | 65187 | 96.03% |
|  | [1, 2, 3, 4] | 63723 | 93.87% | 66800 | 98.35% | 67459 | 99.37% |
| Abruzzo | [1] | 444 | 28.58% | 923 | 59.45% | 1216 | 78.28% |
|  | [2] | 0 | 0.00% | 0 | 0.00% | 0 | 0.00% |
|  | [3] | 904 | 58.24% | 1215 | 78.21% | 1375 | 88.55% |
|  | [4] | 674 | 43.38% | 1014 | 65.31% | 1410 | 90.80% |
|  | [3, 4] | 1157 | 74.50% | 1464 | 94.29% | 1532 | 98.65% |
|  | [2, 3, 4] | 1157 | 74.50% | 1464 | 94.29% | 1532 | 98.65% |
|  | [1, 2, 3, 4] | 1252 | 80.63% | 1525 | 98.17% | 1552 | 99.96% |
| Alto-Adige | [1] | 480 | 69.76% | 626 | 91.03% | 681 | 99.01% |
|  | [2] | 0 | 0.00% | 0 | 0.00% | 0 | 0.00% |
|  | [3] | 0 | 0.00% | 0 | 0.00% | 0 | 0.00% |
|  | [4] | 333 | 48.46% | 432 | 62.82% | 522 | 75.90% |
|  | [3, 4] | 333 | 48.46% | 432 | 62.82% | 522 | 75.90% |
|  | [2, 3, 4] | 333 | 48.46% | 432 | 62.82% | 522 | 75.90% |
|  | [1, 2, 3, 4] | 580 | 84.28% | 669 | 97.22% | 685 | 99.62% |
| Basilicata | [1] | 72 | 12.69% | 101 | 17.86% | 133 | 23.54% |
|  | [2] | 0 | 0.00% | 0 | 0.00% | 0 | 0.00% |
|  | [3] | 109 | 19.25% | 191 | 33.90% | 268 | 47.61% |
|  | [4] | 0 | 0.00% | 0 | 0.00% | 0 | 0.00% |
|  | [3, 4] | 109 | 19.25% | 191 | 33.90% | 268 | 47.61% |
|  | [2, 3, 4] | 109 | 19.25% | 191 | 33.90% | 268 | 47.61% |
|  | [1, 2, 3, 4] | 180 | 31.94% | 292 | 51.76% | 390 | 69.23% |
| Calabria | [1] | 672 | 37.92% | 1190 | 67.15% | 1515 | 85.51% |
|  | [2] | 0 | 0.00% | 0 | 0.00% | 0 | 0.00% |
|  | [3] | 200 | 11.31% | 438 | 24.74% | 711 | 40.14% |
|  | [4] | 554 | 31.28% | 893 | 50.39% | 1205 | 68.02% |
|  | [3, 4] | 754 | 42.58% | 1190 | 67.15% | 1433 | 80.89% |
|  | [2, 3, 4] | 754 | 42.58% | 1190 | 67.15% | 1433 | 80.89% |
|  | [1, 2, 3, 4] | 1357 | 76.61% | 1675 | 94.53% | 1766 | 99.64% |
| Campania | [1] | 4375 | 93.09% | 4620 | 98.29% | 4676 | 99.49% |
|  | [2] | 1344 | 28.60% | 2669 | 56.78% | 3765 | 80.11% |
|  | [3] | 3371 | 71.73% | 4174 | 88.80% | 4418 | 94.01% |
|  | [4] | 3493 | 74.32% | 3899 | 82.95% | 4199 | 89.33% |
|  | [3, 4] | 3846 | 81.83% | 4292 | 91.31% | 4509 | 95.94% |
|  | [2, 3, 4] | 4020 | 85.54% | 4458 | 94.85% | 4607 | 98.03% |
|  | [1, 2, 3, 4] | 4532 | 96.42% | 4653 | 99.00% | 4680 | 99.58% |
| Emilia-Romagna | [1] | 5224 | 85.36% | 5958 | 97.36% | 6061 | 99.03% |
|  | [2] | 0 | 0.00% | 0 | 0.00% | 0 | 0.00% |
|  | [3] | 2462 | 40.22% | 4511 | 73.71% | 5318 | 86.89% |
|  | [4] | 2972 | 48.56% | 5032 | 82.23% | 5883 | 96.12% |
|  | [3, 4] | 4688 | 76.59% | 5671 | 92.67% | 6002 | 98.07% |
|  | [2, 3, 4] | 4688 | 76.59% | 5671 | 92.67% | 6002 | 98.07% |
|  | [1, 2, 3, 4] | 5828 | 95.23% | 6030 | 98.52% | 6092 | 99.54% |
| Friuli-Venezia Giulia | [1] | 1040 | 62.20% | 1443 | 86.30% | 1661 | 99.35% |
|  | [2] | 0 | 0.00% | 0 | 0.00% | 0 | 0.00% |
|  | [3] | 566 | 33.85% | 984 | 58.87% | 1550 | 92.72% |
|  | [4] | 789 | 47.18% | 1165 | 69.69% | 1428 | 85.41% |
|  | [3, 4] | 1311 | 78.43% | 1586 | 94.86% | 1640 | 98.11% |
|  | [2, 3, 4] | 1311 | 78.43% | 1586 | 94.86% | 1640 | 98.11% |
|  | [1, 2, 3, 4] | 1611 | 96.35% | 1657 | 99.13% | 1668 | 99.78% |
| Lazio | [1] | 5014 | 85.23% | 5688 | 96.68% | 5797 | 98.55% |
|  | [2] | 4213 | 71.61% | 5004 | 85.07% | 5424 | 92.19% |
|  | [3] | 4071 | 69.20% | 5086 | 86.46% | 5488 | 93.29% |
|  | [4] | 3570 | 60.69% | 4821 | 81.94% | 5295 | 90.01% |
|  | [3, 4] | 4559 | 77.49% | 5455 | 92.73% | 5702 | 96.92% |
|  | [2, 3, 4] | 5115 | 86.95% | 5680 | 96.56% | 5849 | 99.43% |
|  | [1, 2, 3, 4] | 5705 | 96.97% | 5869 | 99.75% | 5875 | 99.86% |
| Liguria | [1] | 2158 | 78.59% | 2536 | 92.34% | 2713 | 98.80% |
|  | [2] | 0 | 0.00% | 0 | 0.00% | 0 | 0.00% |
|  | [3] | 2289 | 83.37% | 2697 | 98.23% | 2737 | 99.68% |
|  | [4] | 1466 | 53.38% | 1992 | 72.55% | 2303 | 83.85% |
|  | [3, 4] | 2382 | 86.75% | 2702 | 98.41% | 2737 | 99.68% |
|  | [2, 3, 4] | 2382 | 86.75% | 2702 | 98.41% | 2737 | 99.68% |
|  | [1, 2, 3, 4] | 2583 | 94.05% | 2723 | 99.15% | 2741 | 99.84% |
| Lombardia | [1] | 10015 | 92.75% | 10630 | 98.45% | 10722 | 99.29% |
|  | [2] | 300 | 2.78% | 990 | 9.17% | 3289 | 30.46% |
|  | [3] | 8349 | 77.32% | 9698 | 89.81% | 10226 | 94.70% |
|  | [4] | 8613 | 79.76% | 10063 | 93.20% | 10458 | 96.85% |
|  | [3, 4] | 9650 | 89.37% | 10444 | 96.72% | 10675 | 98.86% |
|  | [2, 3, 4] | 9822 | 90.96% | 10567 | 97.86% | 10703 | 99.12% |
|  | [1, 2, 3, 4] | 10608 | 98.24% | 10763 | 99.68% | 10782 | 99.85% |
| Marche | [1] | 1167 | 69.23% | 1636 | 97.03% | 1685 | 99.97% |
|  | [2] | 0 | 0.00% | 0 | 0.00% | 0 | 0.00% |
|  | [3] | 1337 | 79.30% | 1554 | 92.18% | 1649 | 97.83% |
|  | [4] | 275 | 16.33% | 593 | 35.20% | 1039 | 61.60% |
|  | [3, 4] | 1338 | 79.36% | 1554 | 92.19% | 1649 | 97.83% |
|  | [2, 3, 4] | 1338 | 79.36% | 1554 | 92.19% | 1649 | 97.83% |
|  | [1, 2, 3, 4] | 1558 | 92.41% | 1665 | 98.77% | 1685 | 99.97% |
| Molise | [1] | 159 | 46.60% | 211 | 61.79% | 303 | 88.71% |
|  | [2] | 0 | 0.00% | 0 | 0.00% | 0 | 0.00% |
|  | [3] | 101 | 29.49% | 136 | 39.74% | 184 | 53.69% |
|  | [4] | 0 | 0.00% | 0 | 0.00% | 0 | 0.00% |
|  | [3, 4] | 101 | 29.49% | 136 | 39.74% | 184 | 53.69% |
|  | [2, 3, 4] | 101 | 29.49% | 136 | 39.74% | 184 | 53.69% |
|  | [1, 2, 3, 4] | 260 | 75.90% | 316 | 92.44% | 341 | 99.85% |
| Piemonte | [1] | 3553 | 65.09% | 4751 | 87.04% | 5371 | 98.40% |
|  | [2] | 376 | 6.88% | 1252 | 22.94% | 3735 | 68.43% |
|  | [3] | 4386 | 80.34% | 5129 | 93.96% | 5379 | 98.54% |
|  | [4] | 3072 | 56.28% | 4373 | 80.10% | 5073 | 92.93% |
|  | [3, 4] | 4859 | 89.00% | 5307 | 97.22% | 5399 | 98.89% |
|  | [2, 3, 4] | 4947 | 90.63% | 5330 | 97.65% | 5411 | 99.11% |
|  | [1, 2, 3, 4] | 5296 | 97.01% | 5424 | 99.37% | 5441 | 99.67% |
| Puglia | [1] | 3201 | 85.23% | 3619 | 96.37% | 3727 | 99.24% |
|  | [2] | 0 | 0.00% | 0 | 0.00% | 0 | 0.00% |
|  | [3] | 1753 | 46.69% | 2347 | 62.49% | 2986 | 79.52% |
|  | [4] | 2225 | 59.24% | 3191 | 84.98% | 3699 | 98.52% |
|  | [3, 4] | 2815 | 74.96% | 3504 | 93.30% | 3704 | 98.64% |
|  | [2, 3, 4] | 2815 | 74.96% | 3504 | 93.30% | 3704 | 98.64% |
|  | [1, 2, 3, 4] | 3619 | 96.38% | 3705 | 98.68% | 3743 | 99.67% |
| Sardegna | [1] | 1255 | 69.32% | 1551 | 85.62% | 1713 | 94.59% |
|  | [2] | 0 | 0.00% | 0 | 0.00% | 0 | 0.00% |
|  | [3] | 0 | 0.00% | 0 | 0.00% | 0 | 0.00% |
|  | [4] | 879 | 48.53% | 1151 | 63.54% | 1417 | 78.26% |
|  | [3, 4] | 879 | 48.53% | 1151 | 63.54% | 1417 | 78.26% |
|  | [2, 3, 4] | 879 | 48.53% | 1151 | 63.54% | 1417 | 78.26% |
|  | [1, 2, 3, 4] | 1530 | 84.47% | 1769 | 97.71% | 1801 | 99.44% |
| Sicilia | [1] | 4084 | 76.21% | 4741 | 88.47% | 5143 | 95.96% |
|  | [2] | 994 | 18.55% | 1151 | 21.47% | 1285 | 23.98% |
|  | [3] | 1909 | 35.62% | 2859 | 53.35% | 3800 | 70.90% |
|  | [4] | 2255 | 42.09% | 3147 | 58.71% | 3732 | 69.64% |
|  | [3, 4] | 3270 | 61.01% | 4398 | 82.07% | 4923 | 91.86% |
|  | [2, 3, 4] | 3279 | 61.18% | 4399 | 82.08% | 4923 | 91.87% |
|  | [1, 2, 3, 4] | 4757 | 88.76% | 5241 | 97.79% | 5336 | 99.57% |
| Toscana | [1] | 769 | 15.91% | 1813 | 37.51% | 3408 | 70.50% |
|  | [2] | 75 | 1.56% | 160 | 3.32% | 280 | 5.78% |
|  | [3] | 3904 | 80.76% | 4405 | 91.12% | 4697 | 97.17% |
|  | [4] | 1761 | 36.43% | 3235 | 66.93% | 3894 | 80.56% |
|  | [3, 4] | 4065 | 84.10% | 4522 | 93.54% | 4724 | 97.72% |
|  | [2, 3, 4] | 4128 | 85.40% | 4535 | 93.82% | 4724 | 97.73% |
|  | [1, 2, 3, 4] | 4620 | 95.58% | 4807 | 99.44% | 4830 | 99.91% |
| Trentino | [1] | 279 | 47.43% | 564 | 95.84% | 579 | 98.38% |
|  | [2] | 224 | 38.12% | 364 | 61.89% | 442 | 75.24% |
|  | [3] | 0 | 0.00% | 0 | 0.00% | 0 | 0.00% |
|  | [4] | 306 | 51.96% | 405 | 68.89% | 485 | 82.53% |
|  | [3, 4] | 306 | 51.96% | 405 | 68.89% | 485 | 82.53% |
|  | [2, 3, 4] | 337 | 57.28% | 436 | 74.20% | 491 | 83.46% |
|  | [1, 2, 3, 4] | 533 | 90.69% | 571 | 97.13% | 579 | 98.42% |
| Umbria | [1] | 983 | 74.93% | 1216 | 92.66% | 1301 | 99.15% |
|  | [2] | 0 | 0.00% | 0 | 0.00% | 0 | 0.00% |
|  | [3] | 675 | 51.44% | 1047 | 79.83% | 1261 | 96.13% |
|  | [4] | 638 | 48.63% | 942 | 71.82% | 1199 | 91.41% |
|  | [3, 4] | 1118 | 85.18% | 1289 | 98.22% | 1299 | 99.01% |
|  | [2, 3, 4] | 1118 | 85.18% | 1289 | 98.22% | 1299 | 99.01% |
|  | [1, 2, 3, 4] | 1290 | 98.29% | 1312 | 99.96% | 1312 | 100.00% |
| Valle D’Aosta | [1] | 0 | 0.00% | 0 | 0.00% | 0 | 0.00% |
|  | [2] | 0 | 0.00% | 0 | 0.00% | 0 | 0.00% |
|  | [3] | 0 | 0.00% | 0 | 0.00% | 0 | 0.00% |
|  | [4] | 103 | 71.58% | 130 | 90.19% | 138 | 95.83% |
|  | [3, 4] | 103 | 71.58% | 130 | 90.19% | 138 | 95.83% |
|  | [2, 3, 4] | 103 | 71.58% | 130 | 90.19% | 138 | 95.83% |
|  | [1, 2, 3, 4] | 103 | 71.58% | 130 | 90.19% | 138 | 95.83% |
| Veneto | [1] | 4793 | 78.60% | 5909 | 96.89% | 5999 | 98.38% |
|  | [2] | 1680 | 27.55% | 3192 | 52.35% | 4511 | 73.98% |
|  | [3] | 4470 | 73.31% | 5693 | 93.36% | 5936 | 97.35% |
|  | [4] | 3322 | 54.48% | 5073 | 83.19% | 5713 | 93.68% |
|  | [3, 4] | 5018 | 82.30% | 5809 | 95.26% | 5945 | 97.49% |
|  | [2, 3, 4] | 5304 | 86.97% | 5861 | 96.12% | 5952 | 97.60% |
|  | [1, 2, 3, 4] | 5921 | 97.10% | 6005 | 98.47% | 6021 | 98.74% |

#

# Supplementary Table 5: Coverage of stroke unit beds according to the capacity of each hospital per region.

The coverage range of a given hospital increases until its capacity is saturated. The relative capacity is directly proportional to the number of available beds and to restrictions on increasing the coverage area (i.e., blocking by another hospital's catchment area)

| **Region** | **Hospital Name** | **Yearly absolute capacity in the stroke unit (in N of strokes)** | **Yearly relative capacity in the stroke unit (in N of strokes), limited to the coverage area** | **Yearly relative capacity in the stroke unit vs. the no. of strokes in the region (in%)** | **Bed Occupancy Rate** | **Distance to SU bed (median in minutes) [IQR; max]** |
| --- | --- | --- | --- | --- | --- | --- |
| Abruzzo | SS Filippo e Nicola | 548 | 547 | 20.31% | 99.999680% | 52 (38-69; 155) |
|  | Ospedale Civile Spirito Santo | 626 | 626 | 23.21% | 99.999000% | 27 (19-34; 49) |
|  | Ospedale F. Renzetti | 243 | 243 | 9.03% | 99.999780% | 22 (16-27; 36) |
|  | Ospedale Giuseppe Mazzini | 548 | 547 | 20.31% | 99.999680% | 32 (24-40; 99) |
|  | Policlinico SS Annunziata | 209 | 209 | 7.74% | 99.999340% | 20 (15-24; 34) |
|  | San Salvatore | 73 | 73 | 2.71% | 99.998900% | 32 (21-40; 75) |
|  | San Pio da Pietrelcina | 243 | 243 | 9.03% | 99.999780% | 38 (26-50; 82) |
| Alto-Adige | Ospedale centrale di Bolzano | 730 | 730 | 74.53% | 99.999930% | 53 (40-66; 104) |
| Basilicata | AOR San Carlo | 417 | 417 | 40.62% | 99.999920% | 42 (33-51; 93) |
| Calabria | AO SS. Annunziata | 487 | 487 | 16.24% | 99.999800% | 47 (32-66; 139) |
|  | AOU Bianchi-Melacrino-Morelli | 365 | 365 | 12.18% | 99.999580% | 43 (27-67; 136) |
|  | AO Pugliese Ciaccio | 243 | 243 | 8.12% | 99.999800% | 33 (24-45; 114) |
|  | PO Jazzolino | 313 | 313 | 10.44% | 99.999580% | 33 (24-40; 87) |
|  | Ospedale civile San Giovanni di Dio | 209 | 209 | 6.96% | 99.999580% | 46 (30-69; 129) |
| Campania | AORN Cardarelli | 730 | 730 | 9.40% | 99.954690% | 19 (14-25; 40) |
|  | A.O.U. Federico II | 261 | 259 | 3.34% | 99.386630% | 10 (6-15; 23) |
|  | P.O. San Giovanni Bosco | 104 | 99 | 1.28% | 95.031190% | 5 (4-6; 9) |
|  | PO Ospedale del Mare | 304 | 304 | 3.92% | 99.999770% | 11 (7-23; 38) |
|  | PO San Leonardo | 209 | 209 | 2.69% | 99.999330% | 7 (5-9; 50) |
|  | AO S.G. Moscati | 487 | 487 | 6.27% | 99.999720% | 32 (21-40; 82) |
|  | PO Sant'Ottone Frangipane | 626 | 626 | 8.06% | 99.999780% | 55 (37-70; 135) |
|  | AO San Sebastiano | 365 | 365 | 4.70% | 99.999620% | 22 (17-27; 46) |
|  | PO San Giuseppe Moscati | 2,190 | 2,190 | 28.23% | 99.999940% | 24 (17-29; 48) |
|  | PO Luigi Curto | 209 | 209 | 2.69% | 99.999330% | 34 (26-45; 95) |
|  | AOU San Giovanni di Dio e Ruggi D'Aragona | 417 | 417 | 5.38% | 99.999670% | 21 (16-26; 47) |
|  | PO Umberto I | 292 | 292 | 3.76% | 99.999720% | 12 (9-33; 58) |
| Emilia-Romagna | Ospedale Maggiore | 487 | 487 | 5.00% | 99.993020% | 10 (8-12; 17) |
|  | Maggiore | 584 | 584 | 6.00% | 99.999700% | 18 (13-22; 33) |
|  | Ospedale sant'Anna | 313 | 313 | 3.21% | 99.999480% | 18 (14-22; 34) |
|  | Ospedale di Fidenza | 209 | 209 | 2.14% | 99.999480% | 21 (17-25; 35) |
|  | Santa Maria delle Croci | 243 | 243 | 2.50% | 99.999780% | 20 (16-23; 32) |
|  | Santa Maria della Scaletta | 548 | 547 | 5.62% | 99.999880% | 28 (22-34; 61) |
|  | Ospedale Civile di Baggiovara, AOU Modena | 730 | 730 | 7.50% | 99.999480% | 19 (13-23; 39) |
|  | Arcispedale Santa Maria Nuova | 626 | 626 | 6.43% | 99.999830% | 22 (17-26; 40) |
|  | Ospedale Infermi | 1,095 | 1,095 | 11.25% | 99.999880% | 80 (36-94; 123) |
|  | Ospedale Bufalini | 730 | 730 | 7.50% | 99.999780% | 36 (26-47; 87) |
|  | Morgagni-Pierantoni | 292 | 292 | 3.00% | 99.999330% | 22 (15-28; 52) |
|  | Ospedale Guglielmo da Saliceto | 584 | 584 | 6.00% | 99.999700% | 34 (25-44; 126) |
| Friuli-Venezia Giulia | PO Gorizia/Monfalcone | 292 | 292 | 10.55% | 99.999340% | 26 (18-32; 56) |
|  | Azienda sanitaria Universitaria giuliano Isontina | 730 | 730 | 26.38% | 99.999820% | 50 (26-62; 88) |
|  | Santa Maria della Misericordia | 730 | 730 | 26.38% | 99.999820% | 26 (20-31; 78) |
|  | santa Maria degli Angeli | 426 | 426 | 15.39% | 99.999860% | 20 (15-26; 82) |
| Lazio | Azienda Ospedaliera San Camillo | 730 | 730 | 7.07% | 99.999630% | 21 (11-27; 47) |
|  | Fondazione Policlinico Universitario Agostino Gemelli | 487 | 446 | 4.32% | 91.696670% | 12 (9-14; 26) |
|  | Policlinico Campus Bio-Medico | 438 | 438 | 4.24% | 99.999890% | 16 (13-19; 49) |
|  | Ospedale dei Castelli | 365 | 365 | 3.53% | 99.999630% | 14 (11-17; 36) |
|  | Policlinico Tor Vergata | 487 | 487 | 4.71% | 99.995440% | 11 (9-13; 18) |
|  | Policlinico Umberto I | 730 | 729 | 7.06% | 99.867630% | 9 (6-11; 17) |
|  | AOU Sant'Andrea | 438 | 438 | 4.24% | 99.999890% | 13 (10-15; 27) |
|  | Ospedale Belcolle | 209 | 209 | 2.02% | 99.999890% | 20 (15-24; 36) |
|  | Ospedale Fabrizio Spaziani | 438 | 438 | 4.24% | 99.999890% | 29 (22-49; 133) |
|  | Ospedale Santa Maria Goretti | 313 | 313 | 3.03% | 99.999890% | 17 (13-20; 44) |
|  | Azienda Ospedaliera San Giovanni Addolorata | 365 | 364 | 3.52% | 99.635600% | 7 (5-10; 13) |
|  | Ospedale Sant’Eugenio | 313 | 283 | 2.74% | 90.325380% | 9 (6-11; 19) |
|  | Ospedale San Camillo de Lellis | 209 | 209 | 2.02% | 99.999890% | 37 (26-49; 95) |
|  | Ospedale San Filippo Neri | 209 | 209 | 2.02% | 99.999000% | 21 (15-28; 38) |
| Liguria | E.O. Ospedali Galliera | 365 | 365 | 8.77% | 99.999610% | 28 (18-38; 60) |
|  | University of Genova and San Martino Hospital | 1,095 | 1,095 | 26.30% | 99.999860% | 35 (28-40; 63) |
|  | Ospedale Imperia | 365 | 365 | 8.77% | 99.999610% | 24 (16-31; 49) |
|  | Presidio Ospedaliero Sant'Andrea La Spezia | 487 | 487 | 11.69% | 99.999610% | 20 (15-26; 55) |
|  | P.O.M Villa Scassi ASL3 Genova | 243 | 232 | 5.56% | 95.147530% | 16 (11-19; 32) |
|  | Ospedale Santa Corona | 730 | 730 | 17.54% | 99.999990% | 42 (30-52; 86) |
|  | San Paolo | 209 | 188 | 4.51% | 90.005100% | 9 (7-13; 20) |
|  | Ospedale di Lavagna | 122 | 122 | 2.92% | 99.997320% | 7 (5-8; 13) |
| Lombardia | ASST Grande Ospedale Metropolitano Niguarda | 730 | 664 | 4.02% | 90.992570% | 11 (9-13; 17) |
|  | OSPEDALE GUIDO SALVINI GARBAGNATE - ASST Rhodense | 639 | 576 | 3.49% | 90.225080% | 15 (10-23; 36) |
|  | ISTITUTO CLINICO CITTA STUDI | 417 | 330 | 2.00% | 79.222880% | 7 (6-10; 12) |
|  | Ospedale Sacco | 183 | 168 | 1.02% | 92.004980% | 12 (10-14; 19) |
|  | OSPEDALE CIVILE LEGNANO - ASST OVEST MILANESE | 521 | 470 | 2.85% | 90.215650% | 14 (10-21; 47) |
|  | IRCCS Policlinico San Donato | 243 | 224 | 1.36% | 92.187000% | 15 (9-22; 33) |
|  | OSPEDALE SAN CARLO BORROMEO - ASST Santi Paolo e Carlo | 730 | 660 | 3.99% | 90.359120% | 16 (11-22; 35) |
|  | OSPEDALE SAN GERARDO DEI TINTORI- ASST Monza | 626 | 563 | 3.41% | 90.002700% | 11 (8-14; 25) |
|  | ASST Brianza - Vimercate | 584 | 526 | 3.18% | 90.038660% | 18 (12-25; 35) |
|  | OSPEDALE ALESSANDRO MANZONI - ASST Lecco | 365 | 365 | 2.21% | 99.999820% | 34 (19-53; 108) |
|  | OSPEDALE DI DESIO | 730 | 659 | 3.98% | 90.213230% | 15 (11-20; 29) |
|  | OSPEDALE SAN LEOPOLDO MANDIC Merate | 548 | 547 | 3.31% | 99.999820% | 28 (18-62; 99) |
|  | IRCCS OSPEDALE SAN RAFFAELE | 584 | 524 | 3.17% | 89.654700% | 19 (14-26; 38) |
|  | FONDAZIONE IRCCS CA GRANDA OSPEDALE MAGGIORE POLICLINICO | 487 | 438 | 2.65% | 90.005920% | 9 (7-10; 14) |
|  | Istituto Clinico Humanitas | 626 | 589 | 3.56% | 94.116020% | 34 (20-69; 90) |
|  | IRCCS POLICLINICO SAN MATTEO-MONDINO | 730 | 719 | 4.35% | 98.488900% | 31 (22-41; 65) |
|  | OSPEDALE DI VIZZOLO PREDABISSI - ASST Melegnano - Martesana | 261 | 235 | 1.42% | 90.278300% | 15 (11-19; 34) |
|  | Ospedale Civile di Voghera - ASST Pavia | 209 | 209 | 1.26% | 99.999700% | 34 (21-47; 77) |
|  | Ospedale Sant Anna di Como- ASST Lariana | 657 | 657 | 3.97% | 99.999970% | 28 (18-61; 85) |
|  | OSPEDALE VALDUCE - ASST LARIANA | 313 | 313 | 1.89% | 99.999970% | 37 (23-53; 87) |
|  | OSPEDALE MORIGGIA - PELASCINI - ASST Sette Laghi | 146 | 146 | 0.88% | 99.999590% | 15 (11-18; 28) |
|  | Ospedale di Circolo di Saronno - ASST della Valle Olona | 438 | 395 | 2.39% | 90.229770% | 16 (12-20; 33) |
|  | Gallarate- ASST Valle Olona | 548 | 547 | 3.31% | 99.997640% | 18 (13-22; 36) |
|  | OSPEDALE CARLO POMA - ASST Mantova | 456 | 456 | 2.76% | 99.999630% | 28 (21-33; 51) |
|  | Ospedale Maggiore di Lodi - ASST Lodi | 313 | 300 | 1.82% | 95.901870% | 19 (13-25; 45) |
|  | OSPEDALE DI CREMONA - ASST Cremona | 365 | 365 | 2.21% | 99.999820% | 25 (19-31; 53) |
|  | OSPEDALE MAGGIORE DI CREMA- ASST Crema | 365 | 360 | 2.18% | 98.562780% | 25 (17-31; 52) |
|  | Fondazione Poliambulanza Brescia | 487 | 487 | 2.94% | 99.999940% | 24 (20-27; 47) |
|  | Ospedale di Sondrio - ASST Valtellina e dell’Alto Lario | 209 | 209 | 1.26% | 99.999700% | 46 (34-57; 130) |
|  | ASST SPEDALI CIVILI DI BRESCIA | 913 | 912 | 5.52% | 99.999820% | 31 (23-38; 76) |
|  | PRESIDIO OSPEDALIERO DI CHIARI - ASST Franciacorta | 243 | 243 | 1.47% | 99.835980% | 14 (11-18; 28) |
|  | Esine ASST Valcamonica | 243 | 243 | 1.47% | 99.999590% | 40 (29-53; 119) |
|  | ASST Papa Giovanni XXIII BERGAMO | 438 | 438 | 2.65% | 99.999970% | 27 (14-45; 76) |
|  | OSPEDALE BOLOGNINI - ASST BERGAMO EST | 487 | 487 | 2.94% | 99.999940% | 18 (13-22; 38) |
|  | Ospedale Treviglio - Caravaggio ASST Bergamo Ovest | 313 | 288 | 1.74% | 91.965830% | 15 (12-19; 33) |
|  | Ospedale di Varese Fondazione Macchi | 243 | 243 | 1.47% | 99.999590% | 23 (16-27; 35) |
| Marche | Ospedale Santa Croce | 313 | 313 | 10.66% | 99.999360% | 17 (13-21; 30) |
|  | INRCA | 292 | 292 | 9.95% | 99.996450% | 30 (22-38; 47) |
|  | Madonna del Soccorso | 548 | 547 | 18.66% | 99.999980% | 58 (37-73; 123) |
|  | A. Murri | 243 | 243 | 8.29% | 99.999630% | 19 (14-24; 37) |
|  | ospedali Riuniti Ancona | 365 | 365 | 12.44% | 99.998570% | 36 (23-54; 72) |
|  | Carlo Urbani | 438 | 438 | 14.93% | 99.999980% | 35 (25-44; 87) |
|  | Ospedale Provinciale Macerata | 73 | 73 | 2.49% | 99.999100% | 8 (6-10; 14) |
| Molise | PO Cardarelli | 313 | 313 | 66.57% | 99.999530% | 51 (35-66; 107) |
| Piemonte | Ospedale Edoardo Agnelli | 292 | 292 | 3.44% | 99.999640% | 20 (16-24; 69) |
|  | Maggiore della Carità Novara | 548 | 538 | 6.33% | 98.297240% | 34 (23-63; 139) |
|  | Ospedale degli Infermi | 292 | 292 | 3.44% | 99.999640% | 18 (13-22; 51) |
|  | Ospedale Novi Ligure | 313 | 313 | 3.68% | 99.999640% | 32 (21-43; 87) |
|  | Santi Antonio e Biagio | 365 | 365 | 4.30% | 99.999640% | 25 (18-33; 58) |
|  | San Biagio (Ospedale unico plurisede) | 487 | 487 | 5.73% | 99.999640% | 49 (34-65; 122) |
|  | San Giovanni Bosco | 730 | 730 | 8.59% | 99.997470% | 31 (16-43; 63) |
|  | Ospedale Martini | 122 | 110 | 1.29% | 90.136510% | 6 (3-9; 11) |
|  | PO Ciriè | 292 | 292 | 3.44% | 99.999640% | 17 (12-21; 74) |
|  | PO Ivrea | 146 | 146 | 1.72% | 99.999640% | 17 (13-21; 57) |
|  | PO Chivasso | 183 | 182 | 2.15% | 99.999640% | 15 (12-18; 24) |
|  | PO Maria Vittoria | 209 | 188 | 2.21% | 90.145060% | 5 (4-8; 12) |
|  | AO Mauriziano | 417 | 375 | 4.42% | 90.004430% | 7 (5-9; 10) |
|  | PO Moncalieri | 456 | 456 | 5.37% | 99.999860% | 20 (15-24; 34) |
|  | Città della Salute (Molinette) | 417 | 377 | 4.44% | 90.441050% | 12 (7-15; 26) |
|  | Ospedale Santo Spirito Casale | 365 | 365 | 4.30% | 99.999640% | 30 (22-38; 57) |
|  | PO Rivoli | 365 | 365 | 4.30% | 99.999640% | 16 (12-19; 35) |
|  | San Luigi | 209 | 209 | 2.45% | 99.999640% | 15 (11-29; 40) |
|  | PO Borgomanero | 209 | 209 | 2.45% | 99.999640% | 19 (13-27; 46) |
|  | Ospedale Michele e Pietro Ferrero | 292 | 292 | 3.44% | 99.999640% | 27 (22-33; 46) |
|  | PO Savigliano | 274 | 274 | 3.22% | 99.999280% | 21 (17-26; 44) |
|  | Santa Croce e Carle | 209 | 209 | 2.45% | 99.999640% | 15 (11-18; 38) |
|  | PO Asti | 365 | 365 | 4.30% | 99.999640% | 24 (19-29; 43) |
| Puglia | Ospedali Riuniti | 313 | 313 | 5.25% | 99.999920% | 28 (21-33; 53) |
|  | Casa Sollievo della Sofferenza | 209 | 209 | 3.50% | 99.999680% | 34 (25-44; 90) |
|  | Ospedale Dimiccoli | 313 | 313 | 5.25% | 99.999920% | 19 (14-23; 39) |
|  | AOU Policlinico | 417 | 417 | 7.00% | 99.999330% | 22 (15-26; 33) |
|  | Ospedale Di Venere | 313 | 313 | 5.25% | 99.993390% | 18 (12-24; 37) |
|  | Ospedale Miulli | 365 | 365 | 6.13% | 99.999780% | 30 (22-39; 56) |
|  | Ospedale della Murgia F. Perinei | 292 | 292 | 4.90% | 99.999580% | 27 (19-35; 50) |
|  | Ospedale San Giacomo | 209 | 209 | 3.50% | 99.998280% | 15 (12-19; 23) |
|  | Ospedale SS. Annunziata | 521 | 521 | 8.75% | 99.999820% | 24 (18-28; 40) |
|  | Ospedale Perrino | 313 | 313 | 5.25% | 99.999920% | 23 (18-27; 39) |
|  | Ospedale Vito Fazzi | 426 | 426 | 7.15% | 99.999210% | 18 (14-22; 38) |
|  | Ospedale Andria | 156 | 156 | 2.63% | 99.999920% | 16 (12-19; 29) |
| Sardegna | G. Brotzu | 678 | 678 | 23.60% | 99.999980% | 25 (18-58; 99) |
|  | AOU Sassari | 313 | 313 | 10.89% | 99.999630% | 18 (14-22; 40) |
|  | Mater Olbia Hospital | 365 | 365 | 12.71% | 99.999800% | 64 (45-76; 117) |
|  | San Francesco | 313 | 313 | 10.89% | 99.999630% | 57 (42-72; 173) |
| Sicilia | S. Giovanni di Dio | 209 | 209 | 2.54% | 99.999270% | 19 (15-22; 30) |
|  | Sant'Elia | 313 | 313 | 3.81% | 99.999810% | 32 (24-41; 77) |
|  | Umberto I | 104 | 104 | 1.27% | 99.999270% | 49 (31-109; 175) |
|  | Cannizzaro | 365 | 365 | 4.44% | 99.959820% | 13 (10-15; 22) |
|  | Garibaldi Centro | 417 | 417 | 5.08% | 99.998470% | 12 (9-14; 20) |
|  | PO Gravina e San Pietro | 209 | 209 | 2.54% | 99.999270% | 34 (25-46; 81) |
|  | R. Guzzardi | 487 | 487 | 5.92% | 99.999960% | 27 (18-35; 59) |
|  | Umberto I | 584 | 584 | 7.11% | 99.999840% | 41 (30-50; 90) |
|  | Civico | 417 | 417 | 5.08% | 99.999670% | 17 (11-22; 41) |
|  | Villa Sofia | 584 | 584 | 7.11% | 99.999840% | 16 (9-23; 44) |
|  | Buccheri La Ferla | 292 | 292 | 3.55% | 99.996980% | 12 (8-15; 30) |
|  | Istituto Giglio | 104 | 104 | 1.27% | 99.999270% | 34 (26-49; 124) |
|  | S. Antonio Abate | 365 | 365 | 4.44% | 99.999730% | 33 (23-41; 68) |
|  | Policlinico G. martino | 417 | 417 | 5.08% | 99.999670% | 50 (27-69; 103) |
| Toscana | Azienda Ospedaliero Universitaria Senese | 417 | 417 | 5.15% | 99.999560% | 60 (38-79; 112) |
|  | Ospedale Santa Maria Annunziata | 730 | 586 | 7.24% | 80.211050% | 41 (29-52; 94) |
|  | Azienda Ospedaliero Universitaria Pisana | 584 | 522 | 6.45% | 89.358550% | 29 (21-40; 62) |
|  | Ospedale Santa Maria Nuova | 487 | 438 | 5.42% | 90.070200% | 13 (10-17; 34) |
|  | Ospedale del Mugello | 313 | 177 | 2.19% | 56.642850% | 38 (25-49; 93) |
|  | Ospedale Felice Lotti | 183 | 169 | 2.09% | 92.794450% | 18 (12-22; 34) |
|  | Ospedale Santa Maria alla Gruccia - Valdarno | 730 | 730 | 9.02% | 99.999790% | 56 (37-78; 151) |
|  | Ospedale Apuane | 104 | 97 | 1.19% | 92.581890% | 9 (6-12; 22) |
|  | Ospedale della Misericordia | 209 | 209 | 2.58% | 99.999030% | 31 (23-40; 74) |
|  | Ospedale Santo Stefano | 626 | 582 | 7.19% | 93.039990% | 22 (15-30; 72) |
|  | Ospedale SS Cosma e Damiano | 521 | 481 | 5.95% | 92.315170% | 30 (20-43; 69) |
|  | Ospedale San Donato | 209 | 181 | 2.24% | 86.887440% | 56 (38-66; 116) |
|  | Azienda Ospedaliero Universitaria Careggi | 548 | 517 | 6.39% | 94.436540% | 19 (14-25; 42) |
|  | Ospedale San Jacopo | 852 | 538 | 6.65% | 63.190370% | 28 (19-40; 76) |
|  | Ospedale San Giuseppe | 209 | 193 | 2.39% | 92.648350% | 18 (12-25; 45) |
|  | Ospedale San Luca | 548 | 516 | 6.37% | 94.209070% | 41 (25-68; 119) |
|  | Ospedale San Giovanni di Dio | 243 | 230 | 2.84% | 94.523420% | 21 (14-27; 39) |
|  | Ospedale della Bassa Val di Cecina | 156 | 141 | 1.74% | 90.040540% | 16 (12-20; 37) |
|  | Ospedale della Versilia | 730 | 661 | 8.17% | 90.510180% | 59 (41-73; 108) |
|  | Ospedale Civile Elbano Portoferraio | 292 | 70 | 0.86% | 23.809800% | 28 (19-34; 51) |
|  | Spedali Riuniti Livorno | 209 | 208 | 2.57% | 99.863960% | 5 (4-6; 8) |
|  | Ospedale Santa Maria Maddalena | 209 | 209 | 2.58% | 99.999030% | 48 (33-70; 97) |
| Trentino | Ospedale Santa Chiara | 730 | 730 | 70.36% | 99.999930% | 41 (31-50; 110) |
| Umbria | Ospedale Santa Maria della Misericordia | 521 | 521 | 25.34% | 99.999880% | 37 (25-51; 78) |
|  | Ospedale Castiglione del Lago | 313 | 313 | 15.20% | 99.998850% | 39 (29-51; 81) |
|  | Ospedale di Città di Castello | 104 | 104 | 5.07% | 99.998120% | 19 (14-24; 45) |
|  | Ospedale San Giovanni Battista | 209 | 209 | 10.13% | 99.999220% | 17 (12-22; 52) |
|  | Presidio Ospedaliero Gubbio e Gualdo Tadino | 261 | 261 | 12.67% | 99.999440% | 35 (25-52; 129) |
|  | Azienda Ospedaliera Santa Maria Terni | 261 | 261 | 12.67% | 99.999440% | 15 (11-19; 39) |
|  | Ospedale Santa Maria della Stella | 209 | 209 | 10.13% | 99.999220% | 34 (25-42; 62) |
| Valle D’Aosta | Ospedale Umberto Parini | 487 | 255 | 99.40% | 52.490910% | 47 (35-60; 121) |
| Veneto | azienda Ospedaliera universitaria Integrata di Verona | 730 | 730 | 7.78% | 99.999980% | 33 (26-39; 54) |
|  | Piove di Sacco | 52 | 52 | 0.56% | 99.968190% | 5 (4-7; 8) |
|  | IRCSS sacro Cuore Don Calabria | 365 | 342 | 3.65% | 93.829820% | 37 (22-56; 106) |
|  | Ospedale di Feltre | 243 | 243 | 2.59% | 99.999720% | 32 (22-41; 67) |
|  | Ospedale dell'Angelo | 487 | 487 | 5.19% | 99.999720% | 15 (13-18; 54) |
|  | Ospedale di Santorso | 548 | 545 | 5.81% | 99.487490% | 35 (24-49; 89) |
|  | Santa Maria Misericordia | 365 | 365 | 3.89% | 99.999720% | 28 (21-35; 64) |
|  | San tommaso dei battuti | 584 | 584 | 6.23% | 99.999780% | 32 (21-39; 50) |
|  | Ca' Foncello | 417 | 417 | 4.45% | 99.999460% | 17 (13-19; 29) |
|  | San Giacomo | 292 | 292 | 3.11% | 99.995510% | 18 (14-22; 29) |
|  | San Bortolo | 584 | 584 | 6.23% | 99.999780% | 22 (16-28; 46) |
|  | Mater Salutis Legnago | 313 | 313 | 3.33% | 99.999460% | 22 (17-26; 42) |
|  | Ospedale Cazzavillan | 243 | 243 | 2.59% | 99.998930% | 19 (14-24; 43) |
|  | Ospedale san Martino | 209 | 209 | 2.22% | 99.999460% | 36 (23-44; 80) |
|  | Ospedale Pederzoli | 487 | 393 | 4.19% | 80.822700% | 32 (22-45; 79) |
|  | Azienda Ospedale Università Padova | 487 | 487 | 5.19% | 99.998930% | 13 (10-15; 22) |
|  | Ospedale Civile di Cittadella | 511 | 511 | 5.45% | 99.996640% | 20 (14-25; 34) |
|  | Ospedale Riuniti Padova Sud | 243 | 243 | 2.59% | 99.998930% | 17 (13-21; 32) |
|  | Ospedale San Bassiano | 209 | 209 | 2.22% | 99.999460% | 16 (12-21; 43) |
|  | Ospedale di Mirano ULSS3 | 209 | 209 | 2.22% | 99.992100% | 11 (9-13; 20) |
